# *USP18* deubiquitinates and stabilizes SOX9 to promote the stemness and malignant progression of glioblastoma

**DOI:** 10.1038/s41420-025-02522-9

**Published:** 2025-05-15

**Authors:** Zhiyuan Liu, Kuo Yu, Kaile Chen, Yi Zhang, Kexiang Dai, Liang Zhao, Peng Zhao

**Affiliations:** 1 Department of Neurosurgery, The First Affiliated Hospital of Nanjing Medical University, Nanjing, 210000 China; 2 Department of Non-Communicable Disease Prevention, Nanjing Municipal Center for Disease Control and Prevention, Nanjing, 210000 China; 3 Department of Neurosugery, Emergency General Hospital, Beijing, 100028 China; 4 Department of Neurosurgery, The Affiliated Brain Hospital of Nanjing Medical University, Nanjing, 210000 China

**Keywords:** CNS cancer, Cancer stem cells, Oncogenes

## Abstract

Glioblastoma (GBM), the most common and aggressive primary brain tumour, is associated with poor prognosis, primarily due to its stem-like subpopulation, glioblastoma stem cells (GSCs). The deubiquitinase (DUB) family has attracted an increasing amount of attention due to its roles in GSC biology and tumour aggressiveness. In this study, we focused on ubiquitin-specific peptidase 18 (*USP18*), a member of the DUB family whose role in GBM is poorly understood. Through integrated bioinformatics analyses and experimental investigations using patient-derived samples, cell models, and animal models, we elucidated the role of *USP18* in enhancing GSC stemness and promoting malignant behaviours. Our findings revealed that *USP18* expression is significantly elevated in GBM and is correlated with a poor prognosis. Mechanistically, USP18 interacts with SRY-box transcription factor 9 (SOX9), stabilising its protein levels by cleaving K48-linked polyubiquitin chains. Additionally, we identified *YY1* as a transcriptional regulator of *USP18*, increasing its expression in GBM cells. These findings reveal that *USP18* is a potential therapeutic target and highlight the novel *YY1/USP18/SOX9* signalling axis implicated in GBM progression.

## Introduction

Glioblastoma (GBM) is the most common primary malignant tumour of the central nervous system [1]. Currently, the standard treatment for GBM involves a combination of surgery followed by adjuvant radiotherapy or chemotherapy. However, despite these treatments, the median survival of GBM patients is only ~14 months [2]. The highly proliferative and invasive nature of GBM makes complete surgical resection challenging, leading to high rates of postoperative recurrence and mortality [3]. Therefore, there is an urgent need for the identification of effective diagnostic biomarkers and therapeutic targets for this tumour.

GSCs are a distinct subset within glioma; they resemble neural precursor cells, possess self-renewal capacity and exhibit multilineage differentiation potential [4, 5]. Their involvement in the aggressive characteristics of glioma, such as enhanced proliferation, invasion, drug resistance, and recurrence [6], underscores the importance of understanding the molecular mechanisms governing GSCs. In recent years, DUBs have attracted an increasing amount of attention due to their role in maintaining cancer stemness through various mechanisms. DUBs play a pivotal role in maintaining substrate stability by removing ubiquitination modifications, thereby preventing substrate degradation by the proteasome [7]. Among these enzymes, ubiquitin-specific proteases (USPs) constitute the largest subfamily of DUBs [8]. *USP11* has been reported to deubiquitinate and stabilise *CD133*, thereby promoting the cancer cell stemness of small cell lung cancer [9]. Several members of the USP family, such as *USP10*, *USP16*, *USP22*, *USP37* and *USP44*, have been found to modulate key transcription factors of cancer stem cells, including *Nanog*, *Oct4* and *Sox2* [10]. *USP18*, which is also a member of the DUB family, is generally believed to promote the initiation and progression of various cancers. For example, *USP18* enhances melanoma cell resistance to vemurafenib by stabilising *cGAS* expression, thereby inducing autophagy [11]. *USP18* can also impede *NEDD4*-mediated degradation of *CSF1R*, which helps reprogramme tumour-associated macrophages [12]. However, the role of *USP18* in cancer stem cells, particularly GSCs, remains largely unexplored.

*SOX9* is a transcription factor that has been widely recognised for its association with cancer stem cells (CSCs); this factor has been extensively reported to be involved in maintaining the stemness of various cancers, including hepatocellular carcinoma, lung cancer, glioblastoma, gastric cancer, breast cancer, and oesophageal cancer [13]. *SOX9*-expressing hepatocellular carcinoma (HCC) cells exhibit CSC-like characteristics, including bipotent differentiation, self-renewal, high proliferation, colony and sphere formation and resistance to 5-fluorouracil [14]. Additionally, *SOX9* contributes to the maintenance of GSC-like properties in GBM by activating pyruvate dehydrogenase kinase 1 (*PDK1*) via the *PI3K*-*AKT* pathway, a downstream target of *SOX9* [15]. Moreover, it has been reported that *SOX9* can increase the transcription of *SOX2*, thereby promoting the invasion of GSCs [16].

In this study, we demonstrated that *USP18* is significantly upregulated in glioblastoma compared with normal brain tissue, particularly in GSCs, and is correlated with poor patient prognosis. Both in vitro and in vivo experiments confirmed that *USP18* promotes the stemness characteristics of GSCs and enhances malignant phenotypes such as proliferation, invasion, and migration in glioblastoma. Further mechanistic investigations revealed that *USP18* directly functions as a DUB by interacting with *SOX9*, thus facilitating deubiquitination at specific sites to stabilise the *SOX9* protein. Additionally, we identified potential upstream transcription factors regulating *USP18* expression. In summary, our findings suggest that *USP18*, along with its key molecular interactions, may serve as a novel therapeutic target for GBM.

## Results

### *USP18* is highly expressed in glioma cells and GSCs and is associated with poor outcomes in glioma patients

**Fig. 1:**
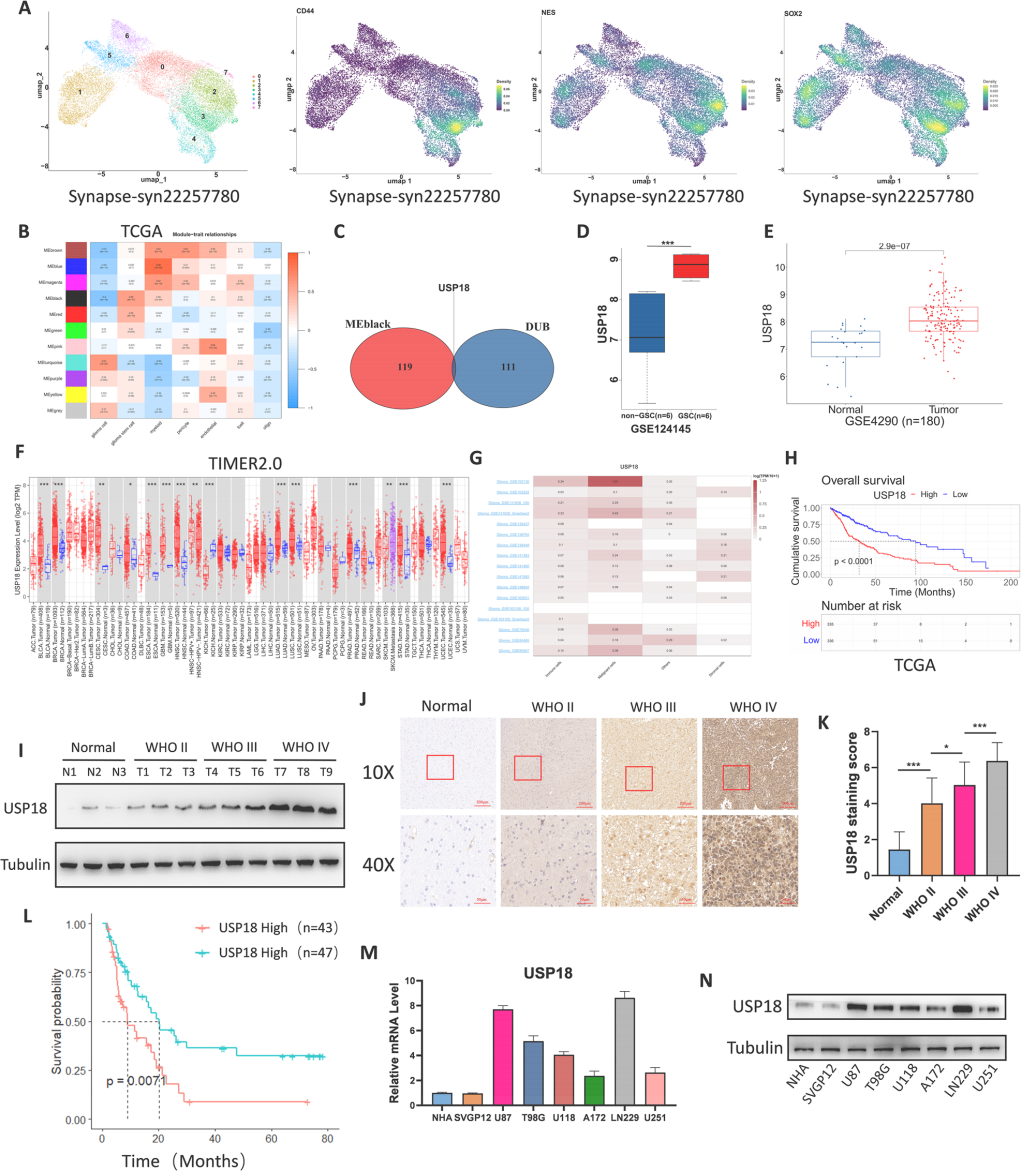
*USP18* is highly expressed in glioma cells, particularly in GSCs, and is associated with poor outcomes in glioma patients. **A** Single-cell RNA sequencing analysis of glioma subpopulations (Synapse dataset ID: syn22257780). Expression levels of stem cell markers (e.g., *CD44*, *Nestin* and *SOX2*) across clusters were visualised using the Seurat R package. **B** Correlations between the expression levels of different gene modules and the proportions of cell subpopulations in GBM tissues. **C** Overlap of the deubiquitinating enzyme family with the MEblack gene module. **D** The expression levels of *USP18* in glioma cells and glioma stem cells were analysed in GSE124145. **E** Comparison of the expression levels of *USP18* between glioma tissues and normal brain tissues in the GSE4290 dataset. **F** Pan-cancer analysis of *USP18* expression (TIMER2.0 database). Tumour vs. normal tissue comparisons were performed using the Wilcoxon rank-sum test. **G** Single-cell transcriptomic distribution of *USP18* across cell types in glioma (TISCH database; 15 datasets). **H** Kaplan–Meier survival curves for glioma patients stratified by *USP18* expression (TCGA cohort; log-rank test). **I** *USP18* protein expression in glioma tissues (WHO grades II–IV) and normal brain tissues was measured by Western blotting. **J**, **K** Representative IHC images of *USP18* in gliomas with different histological grades and NBTs, along with the corresponding statistical results. **L** Kaplan–Meier survival analysis of 90 collected glioma samples on the basis of the *USP18* IHC scores. **M**, **N** *USP18* mRNA (qPCR) and protein (Western blot) levels in glioma cell lines (U87, LN229, U251, T98, U118, and A172) versus normal astrocytes (NHA, SVGP12). All the results are presented as the means ± SDs (from three independent experiments). *P < 0.05, **P < 0.01, ***P < 0.001.

The stem-like characteristics of tumour cells are associated with malignant behaviours such as excessive proliferation, invasion, and migration and poor patient prognosis [17]. The deubiquitinating enzyme (DUB) family is believed to be involved in maintaining the stem-like properties of cancer stem cells [18, 19]. To investigate potential DUBs that could promote glioblastoma stem-like characteristics and malignant progression, we performed dimensionality reduction, clustering, and grouping analysis on glioma cells using the glioma single-cell dataset in the Synapse database. Notably, clusters 2 and 3 highly expressed marker genes characteristic of GSCs (Fig. 1A). Using these single-cell data as a reference, we subsequently predicted the relative proportions of various cell subpopulations in the TCGA-GBM dataset using the BayesPrism algorithm [20]. Furthermore, through weighted gene coexpression network analysis (WGCNA), we calculated the correlation between different gene modules and the proportions of cell subpopulations and found that the MEblack gene module had the highest correlation coefficient with the proportion of GSCs (Fig. 1B). The intersection of the MEblack gene module with the deubiquitinating enzyme family is the *USP18* gene (Fig. 1C). The complete gene list of the MEblack gene module and the DUB family has been uploaded as Supplementary Table S4. Additionally, the GSE124145 dataset also indicated that the expression level of *USP18* in GSCs was significantly greater than that in non-stem cells (Fig. 1D). Additionally, through analysis of multiple glioma GEO public sequencing datasets, we observed a significant increase in *USP18* expression in glioma tissues compared with normal brain tissues (Fig. 1E and Fig. S1A). Given that glioma grade is a significant prognostic factor, we further explored the expression of *USP18* across different WHO grades (II–IV). Box plots revealed a positive correlation between *USP18* transcript levels and increasing glioma grade (Fig. S1B). We further investigated *USP18* expression levels across different molecular subtypes of glioma using the TCGA and CGGA databases. In the TCGA database, we observed that *USP18* expression levels were significantly higher in IDH-wild-type gliomas than in IDH-mutant gliomas (Fig. S1C). Additionally, gliomas with 1p/19q codeletion presented significantly higher *USP18* expression levels than those without 1p/19q codeletion across all datasets (TCGA, CGGA693 and CGGA325) (Fig. S1D). Pan-cancer analysis of *USP18* expression using the TIMER2.0 database [21] revealed elevated expression in most tumour types compared with normal or adjacent cancer tissues (Fig. 1F). Single-cell transcriptomic sequencing provides a clearer understanding of gene expression distributions among different cells within tumour samples. We analysed glioma single-cell data from 15 project sources in the TISCH database [22] and found that *USP18* was expressed predominantly in cancer cells (Fig. 1G). Furthermore, Kaplan‒Meier survival analysis of both the TCGA and CGGA datasets revealed that patients with high *USP18* expression levels had a shorter median survival time than did those with low *USP18* expression levels (Fig. 1H and Fig. S1E, F). Next, we investigated the protein levels of USP18 using Western blot analysis in freshly collected glioma tissues and normal brain tissues (NBTs) obtained from decompressive craniectomy for nontumor indications. We found that USP18 expression was significantly higher in glioma tissues than in normal brain tissues, with levels further increasing with increasing WHO grade (Fig. 1I). The IHC results further validated these findings (Fig. 1J, K). Kaplan–Meier survival analysis of our patient cohort further validated the association between USP18 expression and patient prognosis (Fig. 1L). We also examined both the mRNA and protein expression levels of *USP18* in various glioma cell lines and two human astrocyte cell lines. We observed greater expression of *USP18* to varying degrees in glioma cell lines than in human astrocyte cells (Fig. 1M, N).

### *USP18* silencing inhibits the malignant phenotypes and stemness of glioma cells

**Fig. 2:**
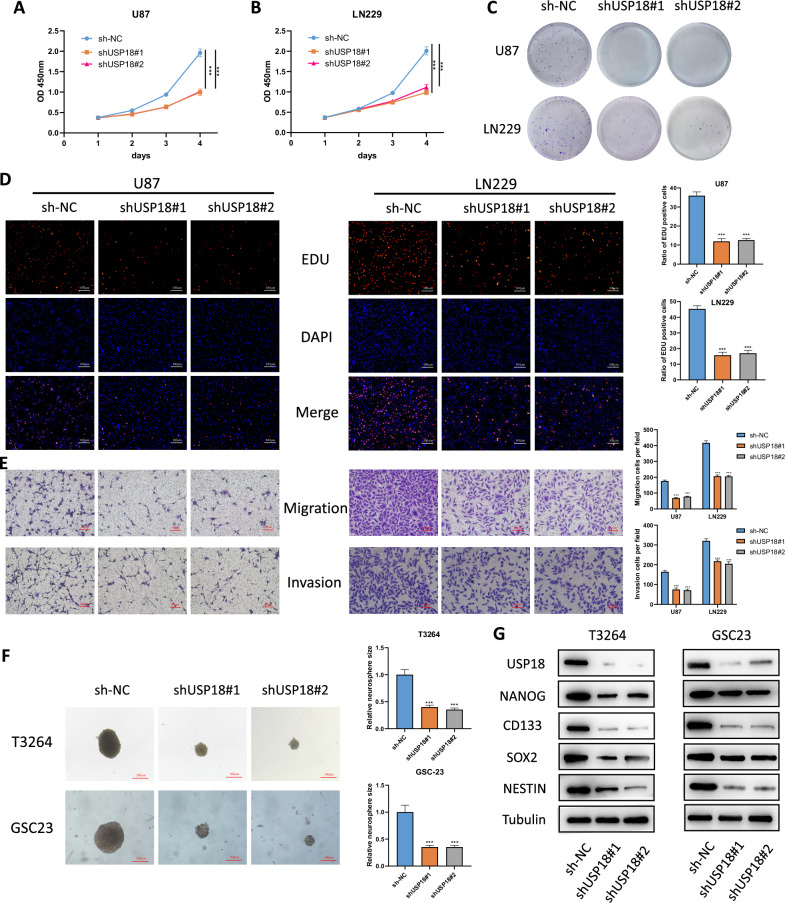
USP18 silencing inhibits the malignant phenotypes and stemness of glioma cells. **A**, **B** Cell viability measured by CCK-8 assay. U87 and LN229 cells transfected with *USP18*-targeting shRNAs (sh*USP18*#1 and sh*USP18*#2) or control shRNA (shNC) were plated in 96-well plates (3 × 10^3^ cells/well; *n* = 6 replicates/group). Absorbance at 450 nm was recorded daily for 5 days. **C** Colony formation assay. Cells (10,000/well) were cultured in 10-cm dishes for 15 days, fixed with 4% formaldehyde, stained with 0.5% crystal violet, and colonies (>100 cells) were counted manually. **D** EdU proliferation assay. Cells were labelled with 10 μM EdU for 2 h, fixed, and nuclei were counterstained with DAPI. EdU-positive cells were quantified using fluorescence microscopy. **E** Transwell migration and invasion assays. Cells (2 × 10^4^) were seeded in serum-free medium in the upper chamber (8-μm pores; Corning). For invasion, Matrigel-coated inserts were used. Migrated/invaded cells were stained and counted after 24 h (three fields/insert). **F** Patient-derived GSCs (GSC23, T3264; 2 × 10^3^ cells/well) were cultured in serum-free medium with EGF (20 ng/mL) and bFGF (20 ng/mL) for 10 days. Spheres were photographed under a light microscope. **G** Western blot analysis of stemness markers (CD133, Nestin, SOX2 and NANOG) in GSCs after *USP18* knockdown. All the results are presented as the means ± SDs (from three independent experiments). *p < 0.05, **p < 0.01, ***p < 0.001.

To investigate the function of *USP18* in glioma cells, lentiviral particles carrying two shRNAs targeting *USP18* were individually introduced into U87 and LN229 cells because of their relatively high expression of *USP18* among all the glioma cell lines. Following puromycin selection, we established stable *USP18*-knockdown glioma cell lines. WB and qPCR confirmed that *USP18* expression was significantly downregulated (Fig. S2A, B). To assess the functional consequences of *USP18* silencing, we conducted a series of cellular experiments. The results demonstrated that *USP18* silencing markedly inhibited cell viability, clonogenicity, and nuclear EdU incorporation in U87 and LN229 cells (Fig. 2A–D and Fig. S2C). Moreover, Transwell migration and invasion assays revealed that *USP18* silencing significantly inhibited cell migration and invasion (Fig. 2E). To further investigate the impact of *USP18* on the stemness of GSCs, we generated *USP18*-knockdown GSC models through lentiviral transduction (Fig. S2D). The neurosphere formation assay demonstrated that *USP18* knockdown resulted in a decreased sphere-forming ability of GSCs (Fig. 2F). Moreover, in vitro limiting dilution assays demonstrated that silencing *USP18* dramatically reduced the frequency of tumoursphere formation in both GSC lines (Fig. S2E). Additionally, to further investigate the role of *USP18* in maintaining the stemness of GSCs, we detected the levels of stemness biomarkers, including CD133, Nestin, SOX2 and NANOG, by Western blotting after *USP18* knockdown. The results revealed that *USP18* knockdown downregulated the protein levels of these stemness biomarkers (Fig. 2G).

### Ectopic expression of *USP18* promotes the malignant phenotypes and stemness of glioma cells in a DUB enzyme activity-dependent manner

**Fig. 3:**
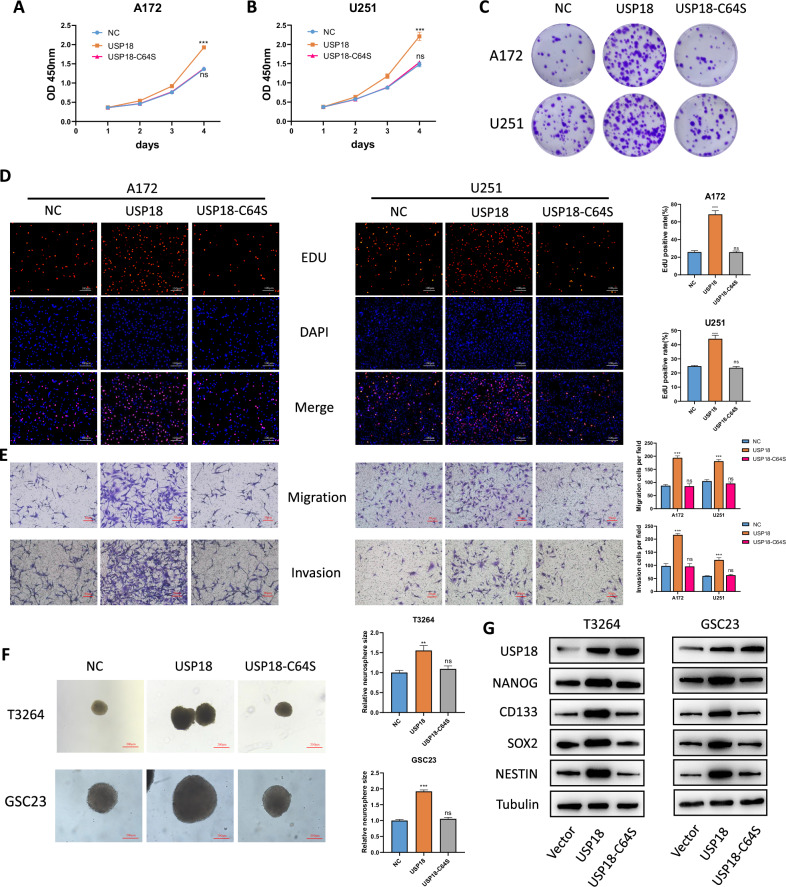
Ectopic expression of USP18 promotes the malignant phenotype and stemness of glioma cells in a DUB enzyme activity-dependent manner. **A**, **B** Cell viability assay (CCK-8) in A172 and U251 cells stably transfected with empty vector (EV), wild-type *USP18*, or catalytically inactive *USP18*-C64S. **C** Representative images of the colony formation assay following *USP18* or *USP18*-C64S overexpression. **D** An EdU assay demonstrated that the overexpression of *USP18*, rather than *USP18*-C64S, enhances the proliferation capability of glioma cells. **E** Representative images and quantitative analysis of Transwell migration and invasion assays in A172 and U251 cells following *USP18* or *USP18*-C64S overexpression. **F** Patient-derived GSCs (GSC23, T3264; 2 × 10^3^ cells/well) were cultured in serum-free medium with EGF (20 ng/mL) and bFGF (20 ng/mL) for 10 days. Spheres were photographed under a light microscope. **G** Western blot analysis of stemness markers (CD133, Nestin, SOX2 and NANOG) in GSCs transfected with *USP18*-WT or *USP18*-C64S. All the results are presented as the means ± SDs (from three independent experiments). *p < 0.05, **p < 0.01, ***p < 0.001; ns not significant.

To further investigate the role of *USP18* in glioma cells, the WT-*USP18* expression vector, the catalytically inactive mutant *USP18*-C64S vector and the empty vector were stably transfected into cells with low *USP18* expression (U251 and A172). Ectopic expression of USP18 was confirmed by Western blotting (Fig. S3A) and qPCR (Fig. S3B). Ectopic expression of WT-*USP18* significantly increased cell viability (Fig. 3A, B), clonogenicity (Fig. 3C and Fig. S3C), and nuclear EdU incorporation (Fig. 3D) in A172 and U251 cells. Migration and invasion assays demonstrated that ectopic expression of WT-*USP18* augmented the migratory and invasive capabilities of glioma cells (Fig. 3E). However, overexpression of *USP18*-C64S did not exert a similar effect on these glioblastoma cell lines. Furthermore, we transfected the same lentiviral vectors carrying the overexpression constructs into two patient-derived glioma stem cell lines, GSC23 and T3264 (Fig. S3D). The neurosphere formation assay confirmed that the overexpression of WT-*USP18* increased the sphere-forming ability of GSCs, whereas the overexpression of *USP18*-C64S did exert a similar effect (Fig. 3F). Limiting dilution assays further demonstrated that overexpression of wild-type USP18 increased the tumoursphere formation frequency of GSCs, whereas its enzymatically inactive mutant counterpart did not exhibit this effect (Fig. S3E). Similarly, Western blot assays revealed that wild-type *USP18*, but not *USP18*-C64S, increased the expression of various stemness markers (Fig. 3G). These results further confirmed the promoting effect of *USP18* on the malignant behaviour and stemness of glioblastoma and indicated that this effect is dependent on its DUB activity.

### USP18 directly interacts with SOX9 and increases the stability of SOX9 in glioma cells

**Fig. 4:**
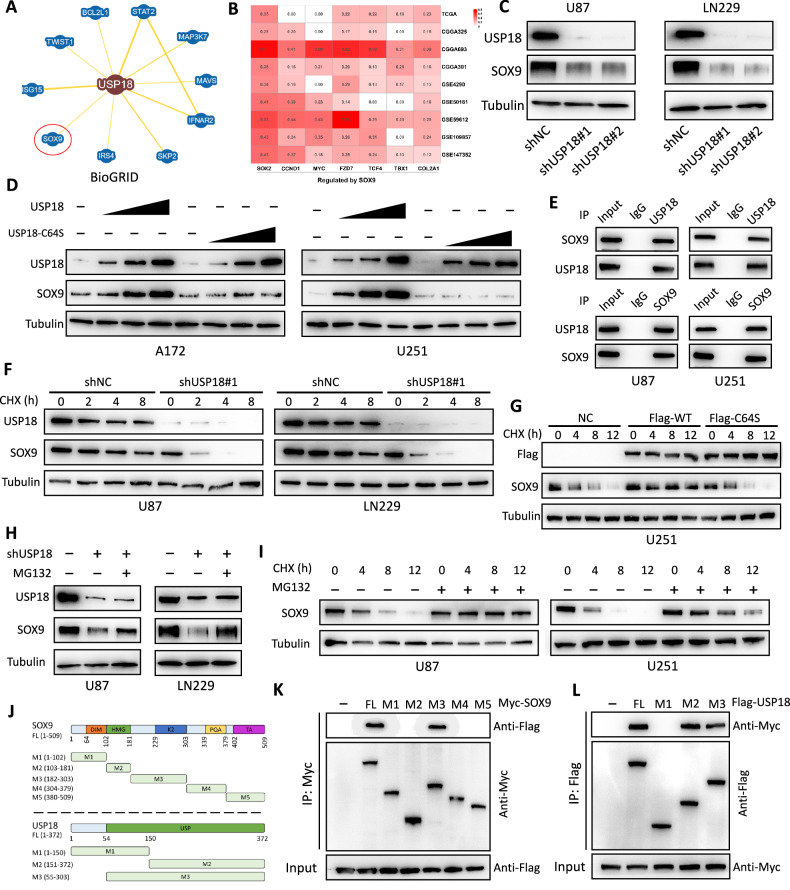
USP18 directly interacts with SOX9 and enhances its stability in glioma cells. **A** Potential genes interacting with *USP18*, as predicted by the BioGRID database. **B** Pearson correlation analysis between *USP18* and *SOX9* downstream targets (*SOX2, COL2A1*, *CCND1*, *MYC*, etc.) across TCGA, CGGA and GEO glioma datasets. **C** Western blot analysis of SOX9 protein levels in U87 and LN229 cells stably transfected with *USP18*-targeting shRNAs (sh*USP18*#1, sh*USP18*#2) or control shRNA (shNC). **D** Dose-dependent effects of *USP18* overexpression in stable cell lines. A172 and U251 cells were infected with lentivirus encoding *USP18*-WT or *USP18*-C64S at increasing MOIs (0, 2, 5, 10). After 7 days of puromycin selection (2 μg/mL), SOX9 protein levels were analyzed by Western blotting. **E** Endogenous USP18 and SOX9 proteins were immunoprecipitated from U87 and LN251 lysates using anti-USP18 or anti-SOX9 antibodies, followed by Western blotting. **F** *USP18* knockdown decreased the stability of the SOX9 protein. The indicated cells were treated with 20 µg/ml CHX for the indicated durations, and protein levels were analysed by Western blotting. **G** Wild-type *USP18*, but not *USP18*-C64S, significantly extended the half-life of the endogenous SOX9 protein. GBM cells transfected with empty vector, *USP18* (WT) or *USP18* (CA) were treated with CHX (20 µg/ml) and collected at the indicated time points for Western blotting. **H** MG132 treatment reversed the decrease in SOX9 protein expression caused by *USP18* depletion. GBM cells transfected with *USP18* shRNA were treated with or without the proteasome inhibitor MG132 (20 μM, 6 h). USP18 and SOX9 protein levels were detected by WB. **I** Levels of the SOX9 protein after different durations of CHX (20 µg/ml) treatment with or without MG132 (20 μM). **J** Schematic diagram of full-length and truncated mutants of *SOX9* and *USP18*. **K** Immunoprecipitation with anti-Myc revealed that SOX9-FL and SOX9-M3 (182–303) could bind to USP18. **L** Immunoprecipitation with an anti-Flag antibody revealed that the C-terminal region of USP18 (amino acids 151–372) is crucial for its interaction with SOX9. All the results are presented as the means ± SDs (from three independent experiments). *p < 0.05, **p < 0.01, ***p < 0.001.

After determining the biological functions of *USP18* in glioma, we explored the potential regulatory mechanisms involved. Using the BioGRID database [23], we predicted potential direct interactors of *USP18*. To ensure the accuracy of our predictions, we set the MINIMUM EVIDENCE threshold to 3 and identified 10 candidate proteins (Fig. 4A). Among these genes, *STAT2*, *MAP3K7*, *MAVS*, *IFNAR2* and *IRS4* have never been reported in relation to GSCs. There are a few sporadic reports suggesting the associations of *BCL2L1* [24], *TWIST1* [25], *ISG15* [26] and *SKP2* [27] with GSCs. However, the role of *SOX9* in promoting glioma stemness and progression has been widely reported [16, 28–31]. In the current study, we demonstrated the positive role of *USP18* in promoting glioma stemness. Therefore, proteins associated with glioma stemness are more likely to be targeted and regulated by *USP18*, especially *SOX9*, because of its widely recognised role in promoting GSCs. To validate this hypothesis, we first conducted preliminary Western blot analysis in U87 and U251 cell lines to examine changes in the expression levels of the candidate proteins following *USP18* overexpression or knockdown. Our results revealed that only SOX9 expression was significantly upregulated or downregulated by changes in *USP18* expression (Fig. S4A). Furthermore, we performed Kaplan‒Meier survival analysis on glioma patient datasets from TCGA and CGGA, which revealed that high *SOX9* expression is correlated with poor clinical outcomes across multiple glioma cohorts (CGGA693-primary glioma, CGGA693-recurrent glioma, TCGA-LGG, and TCGA-GBM). In contrast, the other four proteins were not significantly associated with tumour prognosis in at least some of the datasets (Fig. S5A–E). Additionally, analysis of proteomics data from the Clinical Proteomic Tumour Analysis Consortium (CPTAC) confirmed that SOX9 protein expression in glioma tissue is significantly higher than that in normal brain tissue, and this pattern is consistent with the changes observed for USP18 expression (Fig. S4B). In the TCGA glioma dataset, *SOX9* expression was found to be significantly positively correlated with several GSC markers, further emphasising its role in glioma stemness (Fig. S4C). As a transcription factor, SOX9 regulates numerous downstream genes, including *SOX2*, *COL2A1*, *CCND1*, *MYC*, *FZD7*, *TBX1* and *TCF4* [16, 32–34]. Correlation analysis across multiple datasets revealed a significant positive correlation between *USP18* expression levels and the expression of these downstream targets of *SOX9* (Fig. 4B). These findings strongly suggest that *USP18* likely positively regulates *SOX9* expression. Notably, several of these downstream targets of *SOX9*, such as *SOX2* and *MYC*, have been widely implicated in tumour stemness and aggressive tumour behaviour, further supporting the idea that *USP18* may exert its biological functions through the regulation of *SOX9*. On the basis of these extensive analyses, we selected *SOX9* for further investigation. As shown in Fig. 4C, S6A, *USP18* shRNAs significantly decreased the SOX9 protein levels but had no significant effect on *SOX9* mRNA expression. Conversely, overexpression of *USP18* dose-dependently increased the protein level of SOX9 (Fig. 4D), whereas the *USP18*-C64S mutation, which resulted in loss of enzymatic activity, had no significant effect on SOX9 expression (Fig. 4D). Neither wild-type *USP18* nor catalytically inactive *USP18* altered the mRNA levels of *SOX9* (Fig. S6B). These findings suggest that *USP18* regulates *SOX9* at the protein level rather than at the mRNA level. To further validate the clinical relevance of this regulation, we analyzed SOX9 protein expression by immunohistochemistry in 90 human glioma specimens and correlated its expression with USP18 IHC scores. Notably, SOX9 and USP18 protein levels exhibited a statistically significant positive correlation in glioma tissues (Fig. S6C). Next, we examined whether the USP18 protein directly interacts with the SOX9 protein in cells. Endogenous USP18 and SOX9 proteins were coimmunoprecipitated from lysates of two GBM cell types, confirming a direct interaction between the USP18 and SOX9 proteins (Fig. 4E). Immunofluorescence experiments further confirmed the colocalization of the USP18 protein and the SOX9 protein in glioma cells (Fig. S6D). To further explore the impact of *USP18* on endogenous SOX9 protein stability, we treated cells with the protein synthesis inhibitor cycloheximide (CHX) for various durations. Our results indicated that the knockdown of *USP18* promoted the degradation of SOX9 in glioblastoma cells, thereby shortening its half-life (Fig. 4F and Fig. S7A). In contrast, wild-type *USP18*, but not *USP18*-C64S, significantly extends the half-life of the endogenous SOX9 protein (Fig. 4G and Fig. S7B). Treatment with the proteasome inhibitor MG132 reversed the decrease in the protein level of SOX9 caused by *USP18* knockdown (Fig. 4H). Furthermore, MG132 can extend the half-life of the SOX9 protein under CHX treatment (Fig. 4I and Fig. S7C). USP18 was originally reported to function as a deISGylation enzyme, and ISGylation regulates the stability or degradation of target proteins [35]. To investigate this potential interaction, we assessed the ISGylation level of SOX9 under varying USP18 expression conditions (including overexpression and knockdown). However, no ISGylation of SOX9 was detected in any experimental setting (Fig. S7D). These results suggest that USP18 may regulate SOX9 through mechanisms independent of ISGylation, with the ubiquitin-proteasome pathway likely playing a central role in SOX9 protein degradation. We further explored the minimal binding regions between SOX9 and USP18. The full-length SOX9 (SOX9-FL) protein contains 509 amino acids, which include several functional domains [36, 37]: a dimerisation domain (DIM, amino acids 64-102), a high-mobility group domain (HMG, 102–181), a K2 transactivation domain (229-303), a proline‒glutamine‒alanine (PQA) domain (339–379) and a C-terminal transactivation (TA) domain (402–509). To identify the minimal binding regions, we generated a series of SOX9 truncation mutants, each containing one of these major domains: SOX9-M1 (1–102), SOX9-M2 (103–181), SOX9-M3 (182–303), SOX9-M4 (304–379) or SOX9-M5 (380–509), each tagged with Myc (Fig. 4J). Subsequently, HEK293T cells were transfected with Myc-tagged *SOX9* FL or truncation mutants (M1-M5) along with the Flag-*USP18* vector. Immunoprecipitation with anti-Myc revealed that SOX9-FL and SOX9-M3 (182–303) could bind to *USP18* (Fig. 4K). Similarly, the full-length USP18 (USP18-FL) protein consists of 372 amino acids, including a C-terminal USP domain (amino acids 54–372). We designed three USP18 truncation mutants, namely, *USP18*-M1 (1–150), *USP18*-M2 (151–372) and *USP18*-M3 (54–372) (Fig. 4J), each tagged with Flag. After HEK293T cells were transfected with these constructs, immunoprecipitation with an anti-Flag antibody revealed that the C-terminal region of USP18 (amino acids 151–372) is crucial for its interaction with SOX9 (Fig. 4L). Overall, these results indicate that USP18 and SOX9 physically interact through specific regions and that *USP18* promotes SOX9 protein stability via the ubiquitin‒proteasome pathway.

### *USP18* decreases the K48-linked ubiquitination of SOX9 at the K205 site

**Fig. 5:**
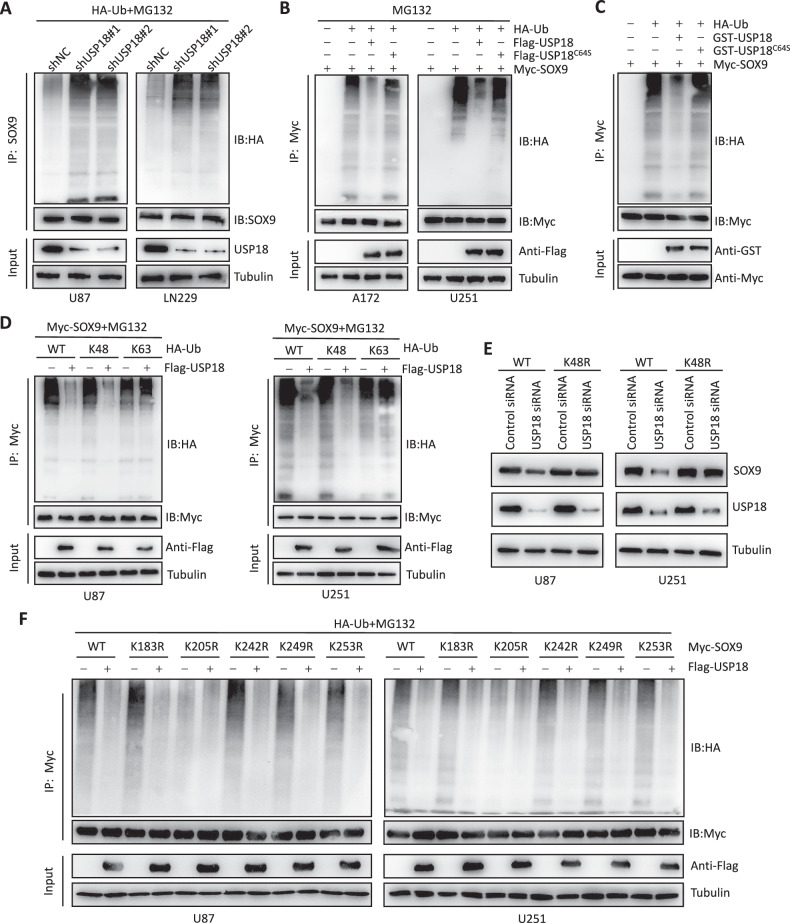
USP18 decreased the K48-linked ubiquitination of SOX9 at the K205 site. **A** Immunoprecipitation assays revealed that *USP18* knockdown increased the accumulation of polyubiquitinated SOX9 in glioma cells. An anti-HA antibody was used to detect HA-tagged ubiquitin. The cells were treated with 20 μM MG132 for 8 h before being harvested. **B** A172 and U251 cells were cotransfected with Myc-*SOX9*, HA-Ub, and either Flag-*USP18* (WT) or Flag-*USP18* (CA). The cell lysates were subjected to IP with an anti-Myc antibody, followed by IB with the indicated antibodies. The cells were treated with 20 μM MG132 for 8 h before being harvested. **C** Ubiquitylated Myc-SOX9 was incubated with GST-USP18 WT or GST-USP18 C64S in deubiquitination buffer. Myc-SOX9 was subjected to IP with Myc, followed by IB with antibodies against HA and Myc. **D** Lysates from cells expressing Myc-SOX9, Flag-USP18, or ubiquitin mutants were pulled down with anti-Myc and then immunoblotted with anti-HA. The input was immunoblotted with anti-Flag. Tubulin was used as a loading control. **E** U87 and U251 cells transfected with Ub WT or Ub K48R were cultured for 72 h in the presence of control siRNA or *USP18* siRNA. The cell lysates were analysed by IB using antibodies against SOX9 and USP18. **F** Immunoprecipitation assays were performed to assess SOX9 ubiquitination in glioma cells transfected with HA-Ub or Myc-SOX9, including its lysine residue mutants (K183R, K205R, K242R, K249R and K253R), with or without Flag-USP18.

We demonstrated that *USP18* promotes SOX9 protein stability via the ubiquitin‒proteasome pathway. Considering the biological function of USP18 as a deubiquitinating enzyme [38–41], we next investigated its impact on the ubiquitination process of SOX9. Immunoprecipitation experiments revealed that *USP18* knockdown significantly increased SOX9 ubiquitination in glioblastoma cells (Fig. 5A). Additionally, we coexpressed Myc-*SOX9* with either wild-type or catalytically inactive mutant *USP18* in A172 and U251 cells. The immunoprecipitation results revealed that coexpressing *USP18*(WT) decreased SOX9 ubiquitination, whereas *USP18*(CA) did not have a similar effect (Fig. 5B). Moreover, in vitro ubiquitylation assays revealed that *USP18*-WT, but not *USP18*-C64S, directly removed ubiquitin from SOX9 (Fig. 5C). To date, two primary forms of polyubiquitin chains have been identified, which are formed through distinct linkage types (Lys48 or Lys63). Lys48-linked ubiquitin chains act as the principal signal for targeting proteins for degradation via the proteasome, whereas Lys63-linked ubiquitin chains participate in various cellular processes independent of proteasomal degradation. Consequently, we investigated which type of polyubiquitin modification on the SOX9 protein is regulated by *USP18*. As shown in Fig. 5D, *USP18* effectively dismantled the Lys48-linked polyubiquitination of SOX9 but had a negligible effect on nondegradative Lys63-linked polyubiquitination. Moreover, in *USP18*-depleted U87 and U251 cells, the expression of a Lys48-resistant (Lys48R) ubiquitin variant rescued *USP18* depletion-induced SOX9 downregulation (Fig. 5E), thus highlighting the crucial role of Lys48-linked polyubiquitination in *USP18*-mediated regulation of SOX9. Together, these findings confirm that USP18 is a deubiquitinating enzyme that targets the SOX9 protein. In the previous part of this study, we identified the interaction between USP18 and the K2 domain of SOX9 (amino acids 182–303). Previous studies [42–44] have indicated that deubiquitination is much more likely to occur within the binding domain. To identify the specific lysine residues, we referred to the UniProt database [45] and retrieved the amino acid sequence for SOX9 corresponding to the 182–303 region. Our examination of this sequence revealed five lysine residues at positions K183, K205, K242, K249 and K253. We mutated each lysine residue to arginine (R). Myc-tagged WT-SOX9 or different mutants were cotransfected with HA-Ub and Flag-*USP18* into glioblastoma cells. As shown in Fig. 5F, the K205R mutation resulted in a significant reduction of SOX9 ubiquitination, and the overexpression of Flag-*USP18* no longer affected the ubiquitination level of SOX9, indicating that *USP18* primarily removes ubiquitin at K205 of SOX9. Furthermore, the CHX treatment assay revealed that the degradation rate of the K205R mutant SOX9 was lower than that of WT-SOX9 (Fig. S8A). These findings highlight the critical role of K205 in SOX9 as a target residue for *USP18*-mediated deubiquitination of SOX9.

### *USP18* regulates the malignant phenotypes and stemness of glioma cells via *SOX9*

**Fig. 6:**
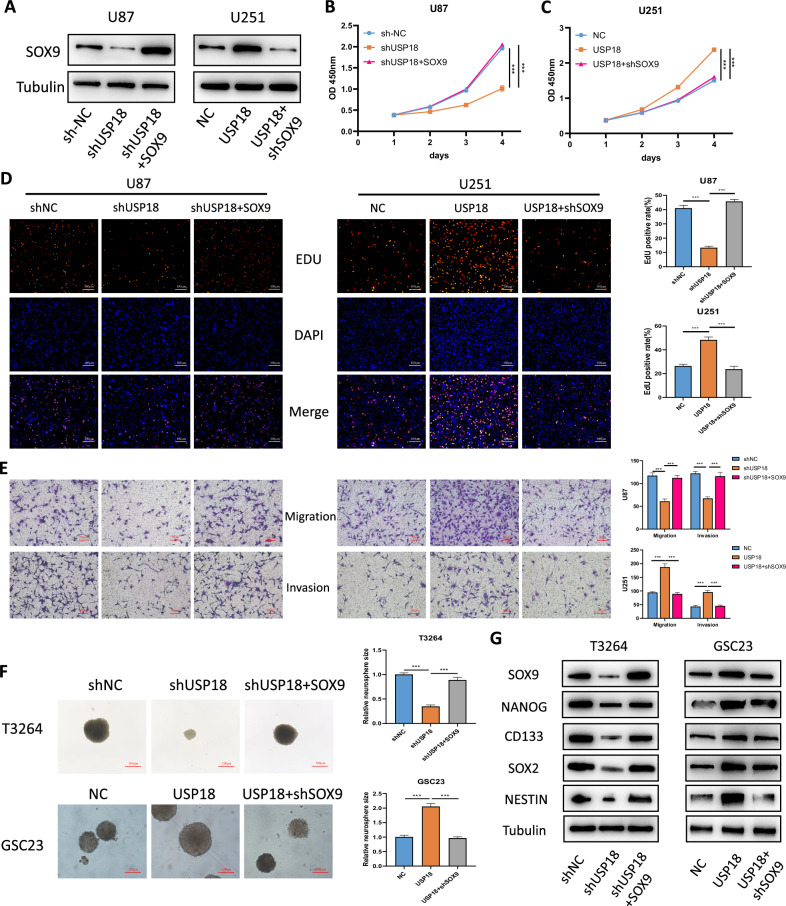
*USP18* regulated the malignant phenotypes and stemness of glioma cells via *SOX9.* **A** Western blot analysis of SOX9 protein levels in rescue experiments. U251 cells stably overexpressing *USP18*-WT were transfected with *SOX9*-targeting shRNA (shSOX9), while U87 cells with *USP18* knockdown were transfected with a *SOX9* overexpression vector. **B**, **C** The viability of glioma cells after the indicated treatments was assessed by a CCK-8 assay. **D** The EdU assay confirmed that *SOX9* treatment rescued the changes in glioma cell proliferation induced by *USP18* knockdown or overexpression. Cells were labelled with 10 μM EdU for 2 h, fixed, and counterstained with DAPI. EdU-positive nuclei were quantified using fluorescence microscopy. **E** Representative images and quantitative analysis of Transwell migration and invasion assays in U87 and U251 cells following the indicated treatments. **F** Neurosphere formation assays confirmed that *SOX9* treatment reversed the changes in the neurosphere formation capability of GSCs caused by USP18 knockdown or overexpression. GSCs (GSC23, T3264; 2 × 10^3^ cells/well) were cultured in serum-free medium with EGF/bFGF for 10 days. Spheres were photographed. **G** Protein levels of stemness markers and SOX9 in GSCs under different treatments. All the results are presented as the means ± SDs (from three independent experiments). *p < 0.05, **p < 0.01, ***p < 0.001.

To confirm whether *SOX9* mediates the biological functions of *USP18* in glioma cells, we conducted a series of rescue experiments. Lentiviruses carrying *SOX9* shRNA were transduced into U251 cells that stably overexpress *USP18*, while lentiviruses containing a *SOX9* expression vector were transduced into U87 cells with stable *USP18* knockdown. The effectiveness of these transfections was confirmed through a Western blot analysis (Fig. 6A). CCK-8, colony formation, EdU incorporation, and Transwell assays revealed that *SOX9* overexpression significantly abrogated the *USP18* shRNA-mediated inhibition of cell proliferation (Fig. 6B–D), cell migration and cell invasion (Fig. 6E). Conversely, when *SOX9* was silenced, these malignant phenotypes promoted by *USP18* overexpression were reversed (Fig. 6B–E). Moreover, treatment with *SOX9* reversed the alterations in the neurosphere-forming ability of GSCs caused by *USP18* knockdown or overexpression (Fig. 6F and Fig. S8B). Additionally, we further observed through Western blotting that the overexpression of *SOX9* reversed the decrease in the expression of stemness markers caused by *USP18* knockdown (Fig. 6G). Conversely, depletion of *SOX9* reduced the levels of stemness markers in GSCs overexpressing *USP18*. Collectively, these findings reveal that *USP18* promotes the malignant phenotypes and stemness of glioma cells by regulating *SOX9*.

### *USP18/SOX9* is essential for glioma growth in vivo

**Fig. 7:**
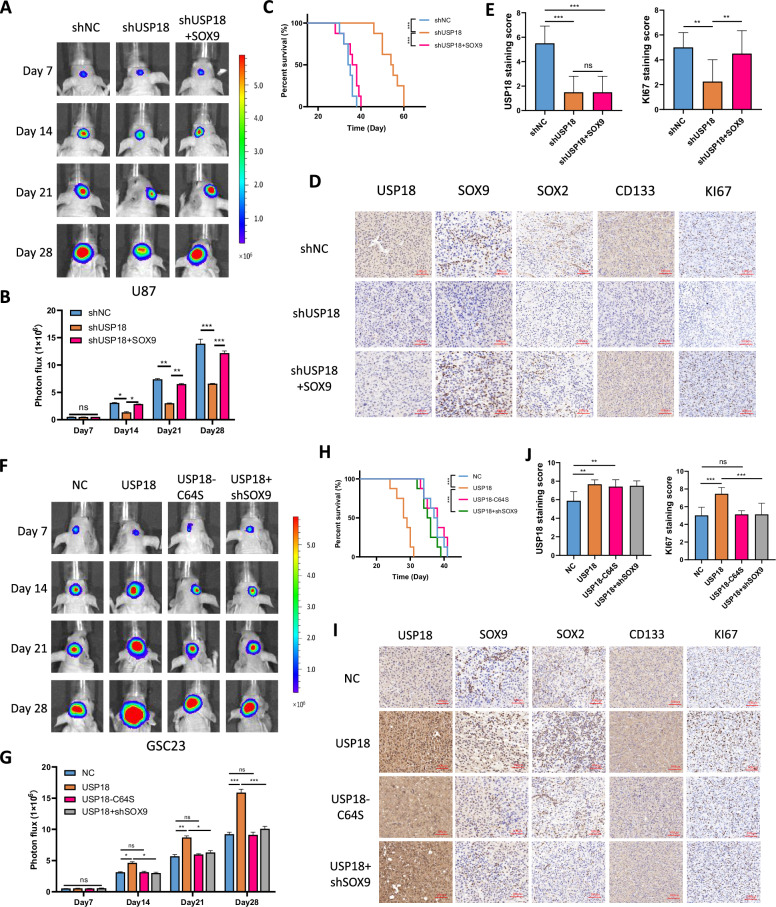
*USP18*/*SOX9* is essential for glioma growth in vivo. **A**, **B** Representative images of the orthotopic xenograft model and quantitative analysis of fluorescence intensity showing that *USP18* knockdown in U87 cells leads to reduced tumorigenicity and that this effect can be rescued by *SOX9*. *n* = 8 for each group. **C** Differences in survival time among the three groups of mice. **D** Representative IHC images of tumour tissues from three groups of mice showing the expression of USP18, SOX9, SOX2, CD133 and Ki67. **E** Statistical analysis of the IHC staining scores for USP18 and KI67 expression. **F**, **G** Representative images of the orthotopic xenograft model and quantitative analysis of fluorescence intensity in the indicated treatment groups. *n* = 8 for each group. **H** Differences in survival time among the four groups of mice. **I** Representative IHC images of tumour tissues from four groups of mice showing the expression of USP18, SOX9, SOX2, CD133 and Ki67. **J** Statistical analysis of the IHC staining scores for USP18 and KI67 expression. All the results are presented as the means ± SDs. *p < 0.05, **p < 0.01, ***p < 0.001; ns not significant.

We demonstrated in vitro that *USP18/SOX9* plays a significant role in glioblastoma malignancy and the maintenance of stemness and explored its underlying mechanisms. To further investigate the impact of *USP18/SOX9* on glioma progression, we established orthotopic xenograft models. Equal numbers of luciferase-labelled U87 cells transduced with control shRNA (shNC), sh*USP18* or sh*USP18* + *SOX9* were intracranially injected into 6-week-old nude mice, and intracranial tumour growth was monitored using bioluminescent imaging. Compared with the control, depletion of *USP18* significantly reduced tumour growth (Fig. 7A, B). Compared with control mice, mice bearing xenografts derived from sh*USP18*-U87 cells exhibited significantly prolonged survival (Fig. 7C). Moreover, overexpression of *SOX9* alleviated the tumour growth inhibition and prolonged survival caused by *USP18* depletion (Fig. 7A–C). Additionally, IHC analysis of tumour tissues from nude mice revealed that *USP18* knockdown resulted in reduced expression of stemness markers (SOX2 and CD133) and a proliferation marker (KI67). This effect was rescued by the re-expression of *SOX9* (Fig. 7D, E and Fig. S8C). Next, equal numbers of luciferase-labelled GSC23 cells transfected with empty vector, *USP18* overexpression vector, *USP18*-C64S overexpression vector or *USP18* + sh*SOX9* vector were implanted intracranially into 6-week-old nude mice. Weekly live imaging revealed that overexpression of wild-type *USP18* significantly increased the luciferase intensity (Fig. 7F, G), indicating faster tumour growth and shorter survival in mice (Fig. 7H). However, the *USP18*-C64S mutant did not exhibit this effect. Interestingly, we also observed that reduced expression of *SOX9* rescued the increased tumour burden and decreased survival caused by *USP18* overexpression (Fig. 7F–H). IHC analysis revealed that overexpression of wild-type *USP18*, but not *USP18*-C64S, increased the expression of GSC stemness biomarkers and Ki67 staining. This effect was reversed by the knockdown of *SOX9* (Fig. 7I, J and Fig. S8D). Overall, the abovementioned results suggest that the *USP18/SOX9* axis plays a critical role in regulating glioma tumorigenesis in vivo.

### YY1 directly binds to the *USP18* promoter to promote its expression

**Fig. 8:**
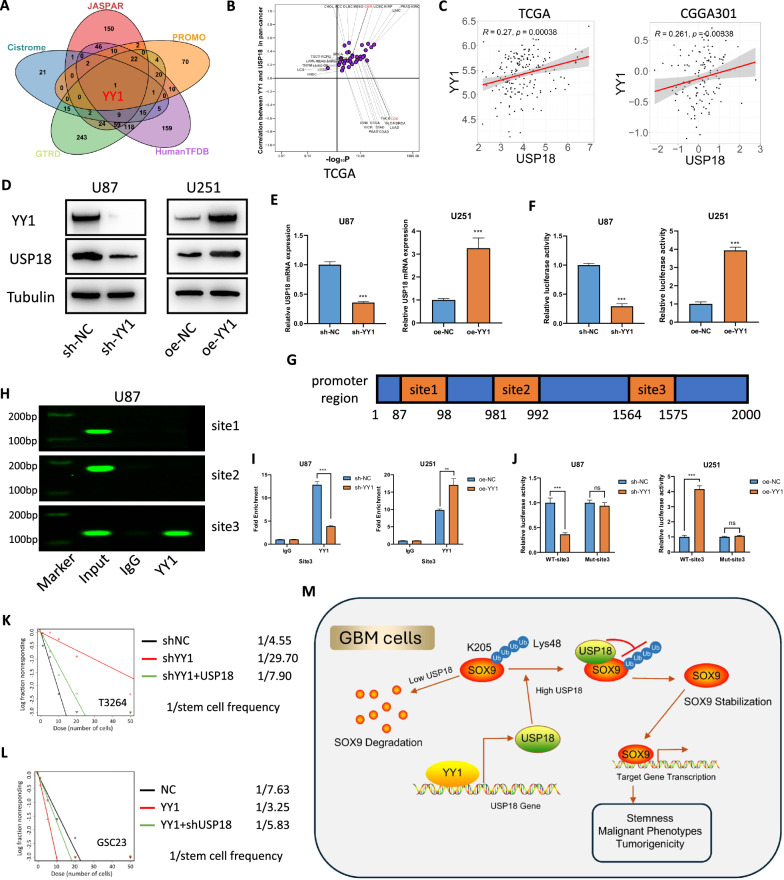
YY1 directly binds to the *USP18* promoter to promote its expression. **A** The Venn diagram visually illustrates the overlap among different databases in predicting transcription factors for *USP18*. **B** The correlation between *YY1* and *USP18* expression in 33 different cancer tissues from the TCGA. **C** In the TCGA and CGGA301 datasets, the expression levels of *YY1* and *USP18* were significantly positively correlated. **D**, **E** Western blot and qPCR analysis of *USP18* in U87 and U251 cells transfected with *YY1*-targeting shRNA (sh-*YY1*) or *YY1* overexpression vector (oe-*YY1*). Both the protein and the mRNA levels of *USP18* are positively regulated by *YY1*. **F** Dual-luciferase reporter gene assays confirmed that *YY1* increased the transcriptional activity of *USP18*. **G** Schematic of *USP18* promoter region with three predicted YY1 binding sites. **H** For ChIP‒qPCR, which targets three potential binding sites, agarose gel electrophoresis of the products confirmed the significant binding of YY1 to site 3. **I** ChIP‒qPCR was conducted in *YY1*-knockdown and *YY1*-overexpressing cell lines, along with their respective controls, further confirming the binding of YY1 to site 3 of the *USP18* promoter region. **J** Mutations in the sequence of binding site 3 abolished the changes in luciferase activity induced by *YY1* overexpression or knockdown. **K**, **L** A limiting dilution assay was used to elucidate the changes in the tumoursphere-forming frequency of GSCs under the indicated treatments. GSCs (GSC23 and T3264) were seeded at 1, 5, 10, 20 or 50 cells/well (10 replicates/density). Tumoursphere frequency was calculated using ELDA software. **M** Schematic diagram showing that USP18, regulated by *YY1*, interacts with SOX9 and promotes deubiquitination at specific sites, thereby stabilising the SOX9 protein and promoting the stemness and malignant progression of GBM. All the results are presented as the means ± SDs (from three independent experiments). *p < 0.05, **p < 0.01, ***p < 0.001; ns not significant.

Given the significant impact of *USP18* on glioma cells, identifying the upstream transcription factors responsible for its regulation is crucial. We predicted potential transcription factors of *USP18* using five different types of transcription factor prediction databases: JASPAR [46], CistromeDB [47], PROMO [48], HumanTFDB [49] and GTRD [50]. The complete prediction results are provided in Supplementary Table S5. *YY1* was selected for further study because it was identified as a common factor across these databases (Fig. 8A). *YY1* is a well-known oncogenic transcription factor that promotes the initiation, progression, or drug resistance of various cancers, including prostate cancer, cervical cancer, bladder cancer, gastric cancer, and colorectal cancer, through multiple mechanisms [51–55]. Notably, some studies have linked *YY1* to the stemness characteristics of cancer stem cells [56–58].

We analysed the correlation between *YY1* and *USP18* expression in 33 cancer tissues from the TCGA and observed a positive correlation between *YY1* and *USP18* across various tumour types (Fig. 8B). Bioinformatics analysis also revealed a positive correlation between the expression levels of *USP18* and *YY1* across multiple glioma public databases (Fig. 8C and Fig. S9A). The knockdown of *YY1* resulted in a reduction in *USP18* expression at both the protein and mRNA levels, whereas *YY1* overexpression led to an increase in *USP18* expression at both levels (Fig. 8D, E). These results further suggest that *YY1* may positively regulate *USP18* transcriptionally. To further confirm the role of *YY1* in *USP18* transcriptional activity, we performed dual-luciferase reporter gene assays by cloning the full sequence of the *USP18* promoter into a luciferase vector. We observed that *YY1* significantly increased the transcriptional activity of *USP18* (Fig. 8F). Using the JASPAR database [46], we identified three potential YY1 binding sites within the *USP18* promoter region (Fig. 8G). We then designed primers targeting the three potential binding sites and performed ChIP‒qPCR experiments. Agarose gel electrophoresis revealed that YY1 significantly binds to site 3 but not to sites 1 and 2 (Fig. 8H and Fig. S9B). We further verified this binding in *YY1*-knockdown and *YY1*-overexpressing cell lines via ChIP‒qPCR (Fig. 8I). Additionally, we mutated the sequence of site 3 and performed reporter gene assays again. We found that the mutation of site 3 abolished the impact of *YY1* knockdown or overexpression on *USP18* transcriptional activity (Fig. 8J). Next, we investigated the impact of *YY1* on glioma stemness and the role of *USP18* in this context. Neurosphere formation and limiting dilution assays revealed that *YY1* knockdown reduced the ability of GSCs to form tumourspheres, whereas *YY1* overexpression increased this ability. Furthermore, restoring *USP18* expression partially reversed these effects (Fig. 8K, L and Fig. S9C, D). The results from the EdU and Transwell assays further confirmed that *YY1* can promote the proliferation, invasion, and migration abilities of glioma cells by regulating *USP18* (Fig. S9E, F). In summary, our results show that YY1 enhances *USP18* transcriptional activity by binding to specific sites within the *USP18* promoter region, thereby exerting its oncogenic effect (Fig. 8M).

## Discussion

Tumour heterogeneity is a significant factor contributing to tumour progression, invasion, migration, and resistance to therapy. Tumour heterogeneity is often attributed to the existence of different populations of cancer cells, with cancer stem cells (CSCs) residing at the top of this hierarchy [4, 17]. Therefore, eliminating CSCs is crucial for the successful treatment of tumours. In recent years, there have been numerous reports on the associations between the DUB family and CSCs. Various DUBs are believed to regulate stem cell core transcription factors or CSC-associated proteins through deubiquitination, thereby promoting the maintenance of tumour stemness and malignant characteristics [18, 19]. However, research specifically examining the correlation between DUBs and glioma stem cells is currently limited. In this study, we identified a member of the DUB family, USP18, which regulates the protein stability of SOX9, thereby promoting the maintenance of glioma stemness and tumour progression.

DUBs are a class of enzymes that regulate the ubiquitination levels of proteins within cells. Ubiquitination is a pivotal protein modification process in which DUBs remove ubiquitin tags from proteins, thereby modulating the degradation rate and stability of target proteins. Through this mechanism, DUBs play crucial roles in regulating the cell cycle, signal transduction pathways, and gene expression [59]. USP18, which was once regarded as a specific deubiquitinase for IFN-stimulated gene 15 (ISG15), recognises and selectively removes ISG15 (a ubiquitin-like protein structurally similar to a dimer of ubiquitin) from modified proteins [60, 61]. However, recent studies have increasingly revealed additional functions of USP18 independent of ISG15. Zhang et al. suggested that USP18 recruits another DUB, USP20, to remove K33- and K48-linked polyubiquitin chains from STING [38]. Another study indicated that USP18 acts as a direct DUB that targets K63-linked polyubiquitin chains on TAK1 [39]. Song et al. proposed that USP18 stabilises the FTO protein by inhibiting its ubiquitination and degradation, thereby activating mitophagy during ischaemic stroke [40]. Furthermore, USP18 has also been reported to regulate reactive astrogliosis by acting as a direct DUB that cleaves Lys48-linked polyubiquitin chains on SOX9 [41]. Notably, while Liu et al. demonstrated USP18-mediated stabilisation of SOX9 in spinal cord injury models [41], our study unveils its distinct oncogenic role in glioblastoma. We identify YY1 as the transcriptional activator of *USP18* and pinpoint K205 as the critical ubiquitination site on SOX9, mechanisms unreported in prior work. These findings establish *USP18* as a novel regulator of cancer stemness and highlight its context-dependent functions in tumour progression.

Increasing evidence has demonstrated that *USP18* exerts oncogenic effects in various cancers. The expression of *USP18* has been shown to be deregulated in lung cancer, breast cancer, bladder cancer, hepatocellular carcinoma and melanoma [35]. It has been reported that *USP18* enhances the resistance of *BRAF*-mutated melanoma cells to vemurafenib by stabilising cGAS expression and inducing autophagy [11]. *USP18* is also highly expressed in colorectal cancer, where it regulates the oncogene Snail1 through the deubiquitination pathway, thus promoting the proliferation, colony formation, migration, and invasion of colorectal cancer cells [62]. Additionally, studies on the role of *USP18* in gliomas are rare. Cai et al. reported that *USP18* deubiquitinates and stabilises Twist1, thus promoting epithelial‒mesenchymal transition in GBM [63]. Li et al. also revealed the critical role of *USP18* in regulating GBM stemness [64]. However, to date, the specific upstream and downstream molecular mechanisms underlying the oncogenic effects of *USP18*, particularly its role in promoting glioma stemness, remain largely unexplored. In this study, we detected high expression levels of *USP18* in glioma cells, particularly in glioma stem cell subpopulations, and found that its expression was correlated with patient prognosis. Through a series of in vitro and in vivo experiments, we further confirmed that *USP18* promotes glioma cell proliferation, invasion, and migration. Importantly, *USP18* is crucial for maintaining the stemness of GSCs. To further explore the mechanisms underlying the oncogenic effects of *USP18*, we utilised the BioGRID database to predict potential proteins that interact with USP18. Among these potential interactors, SOX9, a well-known transcription factor associated with cancer stem cells [65, 66], has emerged as a key candidate. We also observed that the expression levels of *SOX9* downstream target genes are positively correlated with *USP18*, suggesting that *USP18* may positively regulate *SOX9* expression.

*SOX9* is a transcription factor involved in regulating cancer stem cells (CSCs) across various cancers and functions as an oncogene. Increasing evidence indicates that aberrant activation of the *SOX9* signalling pathway contributes to the acquisition of CSC characteristics in cancer types, including glioma [13, 29]. In this study, we found that knocking down *USP18* reduced the protein level of SOX9, whereas overexpressing *USP18* increased the intracellular SOX9 protein level in a DUB activity-dependent manner. Interestingly, *USP18* manipulation did not significantly affect *SOX9* mRNA levels, suggesting that *USP18* influences *SOX9* at the posttranslational level. Further confirmation through coimmunoprecipitation experiments provided additional evidence of a physical interaction between USP18 and SOX9. To delineate the interaction regions between USP18 and SOX9, we generated several truncation mutants of Flag-*USP18* and Myc-*SOX9*. Coimmunoprecipitation analysis revealed that the C-terminal USP domain of USP18 (amino acids 151–372) is crucial for its binding to SOX9 (amino acids 182–303). In contrast to the *USP18*-C64S mutant, USP18 overexpression significantly reduced the K48-linked polyubiquitination of SOX9 by cleaving the K48-linked ubiquitin chains. Furthermore, we generated mutant variants of *SOX9* at specific lysine sites and confirmed that *USP18* enhances deubiquitination at the K205 site of SOX9. To our knowledge, this study represents the first comprehensive investigation of the interaction between *USP18* and the oncogenic transcription factor *SOX9* in gliomas. We not only identified the minimal essential binding regions between USP18 and SOX9 but also identified the critical lysine residues on SOX9 that are essential for its deubiquitination by *USP18*.

Overexpression of *USP18* is commonly observed in tumours [62, 67–69]; however, the underlying mechanisms responsible for its dysregulated expression remain poorly understood. After conducting a thorough analysis across five distinct transcription factor prediction databases, each of which emphasises different aspects, and validating our findings through experimental data, we identified YY1 as an upstream regulator of USP18. YY1 is a well-known oncogenic transcription factor that has been extensively studied due to its role in glioblastoma progression. Bracken et al. reported that *YY1* regulates EMT-related genes such as *E-cadherin*, *Snail*, and *Twist1*, thereby promoting epithelial‒mesenchymal transition, invasion, and metastasis in GBM [70]. Chen et al. reported that *YY1* transcriptionally activates *SNHG5* in GBM cells, which in turn exerts oncogenic effects by activating the *p38/MAPK* pathway [71]. Additionally, *YY1* has been shown to induce *VEGF*-mediated angiogenesis [72]. Studies have also elucidated the relationship between *YY1* and GSCs. For example, You et al. reported that silencing *YY1* reduces the self-renewal capacity of GSCs [73]. Furthermore, *YY1* knockdown in GSCs downregulates genes involved in RNA Pol II transcription, splicing, and m6A modification [74]. In this study, we validated the transcriptional activation of *USP18* by YY1, thus confirming its role as an upstream regulator. Through ChIP and reporter gene assays, we identified the specific binding site of YY1 in the promoter region of *USP18*. To the best of our knowledge, this is the first study to explore the mechanism underlying *USP18* overexpression in gliomas and the transcriptional regulatory relationship between *YY1* and *USP18*, thus providing new insights into the upstream and downstream molecular biological mechanisms driving the oncogenic effects of *USP18*.

In conclusion (Fig. 8M), we identified USP18 as a direct DUB for SOX9 that targets its K48-linked polyubiquitination chains. Through its USP domain, USP18 interacts with SOX9, stabilising the protein and thereby promoting the maintenance of glioblastoma stemness and the progression of malignant phenotypes. Furthermore, the transcription factor YY1 enhances *USP18* expression by directly binding to specific sites within the *USP18* promoter region. Our study underscores the potential of targeting the *YY1/USP18/SOX9* axis as a therapeutic strategy for glioblastoma.

## Methods and materials

### Inference of cell subpopulation proportions

Glioma single-cell data were obtained from Synapse (https://www.synapse.org/) with the dataset ID syn22257780 and processed using the standard workflow of the Seurat R package. After glioma cells were extracted, a more detailed clustering analysis was performed, and the proportion of glioma stem cells in the TCGA-GBM dataset was estimated using the BayesPrism R package.

### Source of *USP18* expression data

Sequencing data for glioma stem cells and glioma cells were obtained from GSE124145. Sequencing data for glioma and normal brain tissues were obtained from the GSE4290, GSE50161, GSE59612, GSE109857 and GSE147352 datasets. Data for gliomas of various grades were derived from the TCGA (https://www.cancer.gov/ccg/research/genome-sequencing/tcga) and CGGA (http://www.cgga.org.cn/analyse/RNA-data-expression-distribution-result.jsp) databases. The expression-distribution information of USP18 at the single-cell level was obtained from the TISCH (http://tisch.comp-genomics.org/gallery/) database.

### Survival analysis

Patients were stratified into high and low USP18 expression groups on the basis of the median value of *USP18* expression. Survival analysis was conducted using the log-rank test, implemented in the survival and survminer R packages.

### Transcription factor prediction

Upstream transcription factors for *USP18* were predicted using data from multiple databases, including JASPAR (https://jaspar.elixir.no/), Cistrome (http://cistrome.org/), PROMO (https://alggen.lsi.upc.es/cgibin/promo_v3/promo/promoinit.cgi?dirDB=TF_8.3), GTRD (https://gtrd20-06.biouml.org/) and HumanTFDB (https://guolab.wchscu.cn/AnimalTFDB#!/).

### Cell culture

The human glioblastoma cell lines LN229, U87, U118, A172 and U251 were purchased from the National Collection of Authenticated Cell Cultures (Shanghai, China). T98 cells and HEK293T cells were purchased from the American Type Culture Collection (Shanghai, China). The human normal astrocyte cell lines NHA and SVGP12 were purchased from Fenghbio (Hunan, China). All these cell lines were cultured in DMEM (Gibco, United States) supplemented with 10% foetal bovine serum (FBS) and 100 μg/mL penicillin‒streptomycin. The patient-derived glioblastoma stem cell lines GSC23 and T3264 were kind gifts from the Institute for Brain Tumours of Nanjing Medical University [75]. GSCs were cultured in neurobasal medium (Invitrogen) supplemented with B27, epidermal growth factor (EGF), basic fibroblast growth factor (FGF), and penicillin‒streptomycin. All the cell lines were authenticated by STR profiles and tested for mycoplasma contamination every 4 months.

### Human tissue samples and immunohistochemical staining

A total of 90 glioma tissue samples (including 20 diffuse astrocytomas, 22 anaplastic astrocytomas and 48 glioblastomas) were collected from the Department of Neurosurgery at The First Affiliated Hospital of Nanjing Medical University. The general information of the patients and the IHC scoring data are provided in Supplementary Table S1. All patients provided written informed consent. The study was conducted in accordance with the ethical principles outlined in the Declaration of Helsinki and was approved by the Institutional Review Board of Nanjing Medical University (Nanjing, China). Paraffin-embedded sections of human glioma tissues were subjected to immunohistochemical staining with an antibody against USP18. The immunostaining was then quantitatively assessed based on the proportion of positively stained cells and the intensity of the staining as described previously. For the proportion score, the following scale was used: 0 for 0% positive tumour cells, 1 for 0–1%, 2 for 2–10%, 3 for 11–30%, 4 for 31–70% and 5 for 71–100% positive cells. The staining intensity was rated on a scale of 0 to 3: 0 indicated no staining, 1 indicated weak staining, 2 indicated moderate staining, and 3 indicated strong staining. The final score was calculated by adding the proportion and intensity scores, resulting in a total score ranging from 0 to 8. Three pathologists, who were blinded to the clinical data of the patients, independently performed the IHC scoring.

### CCK-8 assay

The glioma cells were plated in a 96-well plate (3 × 10^3^ cells/well) with 100 μL of complete culture medium. Then, on the indicated day, 10 μL of CCK-8 reagent (Beyotime, C0037, China) was added to each well, and the plates were incubated for 2 h. After that, the absorbance values at 450 nm were measured using a spectrophotometer.

### Colony formation assay

Glioma cells subjected to the indicated treatments were initially seeded at 10,000 cells per well into 10-cm cell culture plates. The complete medium was renewed every 3 days (for a total of 15 days). The plates were subsequently washed with PBS twice, fixed with 4% formaldehyde for 30 min, and stained with 0.5% crystal violet for 1 h. Colonies containing >100 stained cells were manually counted.

### EdU assays

EdU assays were conducted using an EdU Proliferation Kit (Beyotime, C0071S, Shanghai, China) following the manufacturer’s instructions. Glioma cells were seeded in a six-well plate (2 × 10^5^ cells/well). After the experimental procedures were performed according to the manufacturer’s instructions, images were captured using an Olympus FSX100 microscope (Olympus, Tokyo, Japan).

### Transwell assay

For the migration assay, Transwell chambers (Corning, USA) with 8-μm pores were utilised. A total of 2 × 10^4^ cells were seeded into the upper surface of the chamber and cultured with DMEM without FBS. Simultaneously, the lower chamber was filled with DMEM containing 10% FBS. After 24 h of migration, the cells that had migrated to the lower chamber were fixed, stained, photographed, and counted. For the invasion assay, a layer of “Matrigel” was coated on the chamber, and the remaining steps were the same as those for the migration assay.

### Quantitative polymerase chain reaction (qPCR)

Total RNA was extracted with TRIzol reagent (Thermo Fisher Scientific, USA) and reverse transcribed with HiScript III RT SuperMix for qPCR (Vazyme Biotech). For data quantification, the 2^ΔΔCt^ method was utilised, with GAPDH used as the housekeeping gene for normalisation. All the primers used are listed in Table S2.

### Western blotting

The cells or samples were lysed in RIPA buffer (Beyotime) containing protease and phosphatase inhibitors. The lysates were subjected to sodium dodecyl sulfate‒polyacrylamide gel electrophoresis (SDS‒PAGE) for protein separation and then transferred onto a nitrocellulose membrane, which was subsequently blocked with 5% nonfat milk for 2 h and incubated with primary antibodies. The primary antibodies used for WB analysis were purchased from Cell Signaling Technology (#4813, #5568, #3580, #64326, #3579, #73349, #82630, #2276, #3724, #2624, #46395, #2743) or Abcam (ab205606). Following incubation with HRP-linked secondary antibodies, the signals were detected using the SuperSignal® Maximum Sensitivity Substrate (Thermo Fisher Scientific).

### Immunofluorescence

GBM cells were fixed in 4% paraformaldehyde for 20 min and permeabilised with 0.25% Triton X-100. The cells were subsequently blocked with 1% bovine serum albumin (BSA) for 1 h and incubated overnight with specific primary antibodies (#53229, Cell Signaling Technology; ab185966, Abcam). Following three washes with PBS-T, the cells were incubated with the appropriate secondary antibodies for 1 h. Nuclei were counterstained with DAPI, and images were acquired using confocal laser-scanning microscopy (Zeiss LSM 5).

### Lentiviral transfection

Purified lentiviral vectors with puromycin resistance for the silencing or expression of *USP18*, *SOX9*, *YY1*, Flag-tagged *USP18* and truncation mutants, Flag-tagged *USP18-C64S*, Myc-tagged *SOX9* and mutants, HA-Ub, HA-Ub-K48 and HA-Ub-K63 were obtained from Genechem (Shanghai, China). The plasmids were constructed using the GV102 vector backbone (Genechem) with a puromycin resistance marker. For transient expression experiments, cells were transfected with plasmids using Lipofectamine 3000 (Invitrogen) according to the manufacturer’s protocol, and harvested 48 h post-transfection for analysis. For stable cell line generation, lentiviral particles were produced by co-transfecting HEK293T cells with the target plasmid, psPAX2 (packaging plasmid), and pMD2.G (envelope plasmid) using polyethylenimine (PEI). Viral supernatants were collected 48 and 72 h post-transfection, concentrated via ultracentrifugation, and titrated using a qPCR-based method. Target cells were infected with lentivirus at a multiplicity of infection (MOI) of 5 in the presence of 8 μg/mL polybrene. After 48 h, cells were selected with 2 μg/mL puromycin for 7 days to obtain stable cell pools. Knockdown or overexpression efficiency was validated by qPCR and Western blotting. The shRNA sequences used are listed in Table S3.

### Neurosphere formation assay

GSCs were seeded (2 × 10^3^ cells/well) in ultralow attachment six-well plates and cultured in serum-free DMEM supplemented with EGF, bFGF or B27 for 10 days. The cells were then fixed with 4% formaldehyde and photographed under a light microscope.

### Chromatin immunoprecipitation (ChIP)

ChIP was performed using a ChIP kit (Abcam, #ab500). Briefly, cells were cross-linked with formaldehyde, and chromatin was isolated and fragmented by sonication. Antibodies specific to the protein of interest (#46395; Cell Signaling Technology) were used to immunoprecipitate the protein‒DNA complexes. After the reversal of cross-linking, the DNA was purified and analysed by qPCR or agarose gel electrophoresis to identify regions of interest. Negative control samples without antibodies were also included.

### Dual-luciferase reporter gene assay

The *USP18* luciferase reporter plasmid and control Renilla luciferase reporter plasmid were constructed by GeneChem. Plasmids containing the firefly luciferase reporter gene and a control Renilla luciferase gene were transfected into cells. After a specified time, the cell lysates were collected, and the luciferase activity was measured using dual-luciferase assay kits (Cat. No. RG027; Beyotime Biotechnology).

### Limiting dilution assays

For the in vitro limiting dilution assay, cells were seeded at the indicated densities, with ten replicates per group, in a 96-well plate. Tumoursphere formation and counts were recorded on day 7 after seeding. The neurosphere formation efficiency was analysed using the software available at http://bioinf.wehi.edu.au/software/elda/.

### Coimmunoprecipitation assay

To perform coimmunoprecipitation, cells were first lysed in lysis buffer (catalogue P0013, Beyotime) containing both protease inhibitors and phosphatase inhibitors, which prevent protein degradation and dephosphorylation during the process. After lysis, the protein concentration was determined using a BCA assay kit (catalogue P0009, Beyotime), and equal amounts of total protein were incubated overnight at 4 °C with the indicated primary antibodies (anti-USP18, anti-SOX9, anti-Myc, and anti-FLAG) in a gently shaking incubator. Following incubation, protein A/G agarose beads (catalogue P2055, Beyotime) were added to the lysates and incubated for an additional 3 h at 4 °C to allow the antibodies to bind to the beads. The beads were then washed four times with cold lysis buffer to prevent nonspecific binding. The immunoprecipitated proteins were eluted from the beads by boiling in SDS‒PAGE loading buffer and analysed by Western blotting (WB) using appropriate primary and secondary antibodies.

### Protein stability assay

For the protein stability assay, the cells were treated with 20 μg/ml cycloheximide (CHX; Sigma‒Aldrich) to inhibit protein synthesis. The cells were then harvested at various time points (0, 2, 4 and 8 h or 0, 4, 8 and 12 h) after CHX treatment. At each time point, the cells were lysed in RIPA buffer containing protease inhibitors and phosphatase inhibitors to prevent degradation or modification of the proteins. The protein concentration of the lysates was determined using a BCA assay kit (catalogue P0009, Beyotime), and equal amounts of protein were separated via SDS‒PAGE. To monitor protein degradation, Western blotting was performed using an antibody specific to the target protein, SOX9. The relative protein levels were quantified using ImageJ software to measure the intensity of the target protein bands. A plot of the band intensity over time was generated to visualise protein degradation.

### In vitro deubiquitylation assay

HEK293T cells were transfected with vectors encoding Myc-*SOX9* and HA-ubiquitin and cultured for 36 h. After that, the cells were treated with 20 μM MG132 for 8 h to block protein degradation and increase the accumulation of polyubiquitinated proteins. The cells were then harvested, and total protein was extracted using cold RIPA lysis buffer containing protease and phosphatase inhibitors. The extracts were clarified by centrifugation at 12,000×*g* for 10 min at 4 °C to remove cell debris. Then, the cell lysates were incubated overnight with anti-Myc agarose beads (Thermo Fisher) at 4 °C to purify ubiquitinated SOX9 protein, followed by washing the beads four times with cold lysis buffer to prevent nonspecific binding. For the deubiquitination step, the ubiquitinated SOX9 was incubated with recombinant GST-USP18(WT) or GST-USP18(CA) in deubiquitination buffer (50 mM Tris-HCl pH 7.5, 50 mM NaCl, 1 mM MgCl2, 10 mM DTT and 5% glycerol) for 2 h at 37 °C to remove the ubiquitin chains. Then, the immunocomplexes were washed with cold lysis buffer four times, eluted in loading buffer, and boiled at 95 °C for 10 min. The mixture was analysed by immunoblotting with anti-HA and anti-Myc antibodies.

### In vivo deubiquitination assay

Glioma cells were transfected with Myc-*SOX9* and its mutants, Flag-*USP18* and its mutants, and HA-Ub vectors. After 48 h, the cells were treated with 20 μM MG132 proteasome inhibitor for 8 h to block protein degradation. The cells were then harvested and lysed in NETN buffer supplemented with 0.1% SDS, 20 μM MG132, and protease inhibitors. The lysates were incubated with anti-SOX9 or anti-Myc antibodies for 8 hours at 4 °C, followed by incubation with protein A/G agarose beads for an additional 3 h at 4 °C to pull down the protein complexes. The immunoprecipitates were then washed extensively with NETN buffer containing 0.1% SDS and subjected to Western blot analysis. The blots were probed with antibodies against SOX9, USP18, Myc, Flag and HA to detect the ubiquitylation status of SOX9 and its interaction with USP18 and its mutants.

### Xenograft mouse model

Six-week-old male nude mice were obtained from Nanjing Medical University Animal Center. The mice were randomly allocated to experimental groups, with each group consisting of eight mice, and were implanted with luciferase-transfected U87 or GSC23 cells at a dose of 500,000 cells per mouse in the frontal subdural region. In vivo optical imaging was performed weekly to monitor tumour growth. Prior to imaging, each mouse received an intraperitoneal injection of 10 mg d-luciferin (YEASEN, Shanghai, China). All animal procedures were approved by the Animal Management Rule of the Chinese Ministry of Health and the Nanjing Medical University Animal Experimental Ethics Committee. The investigators were blinded to the group allocation of the mice during the experiment.

### Statistical analysis

Statistical analysis was performed with GraphPad Prism software (v8.0.3). Student’s *t*-test was used to analyse differences between the two groups. One-way ANOVA and Tukey’s post hoc test were used to compare three or more groups. All the statistical analyses were two-tailed. The results are shown as the means ± SDs and were obtained from at least three independent experiments. *P < 0.05 was considered statistically significant, with the level of significance indicated as *p < 0.05, **p < 0.01 and ***p < 0.001.

## Supplementary information

original Western blot

Figure S1-S9

Supplementary Figure S1-S9 legends

Table S1

Table S2-S3

Table S4

Table S5

## Data Availability

All data used in this work can be acquired from the corresponding author upon reasonable request.
