# Supplementary material for: USP18 deubiquitinates and stabilizes SOX9 to promote the stemness and malignant progression of glioblastoma
Source: Cell Death Discov. 2025 May 15;11:237. doi: 10.1038/s41420-025-02522-9 (PMC12081856; doi:10.1038/s41420-025-02522-9)

Figure 1 I

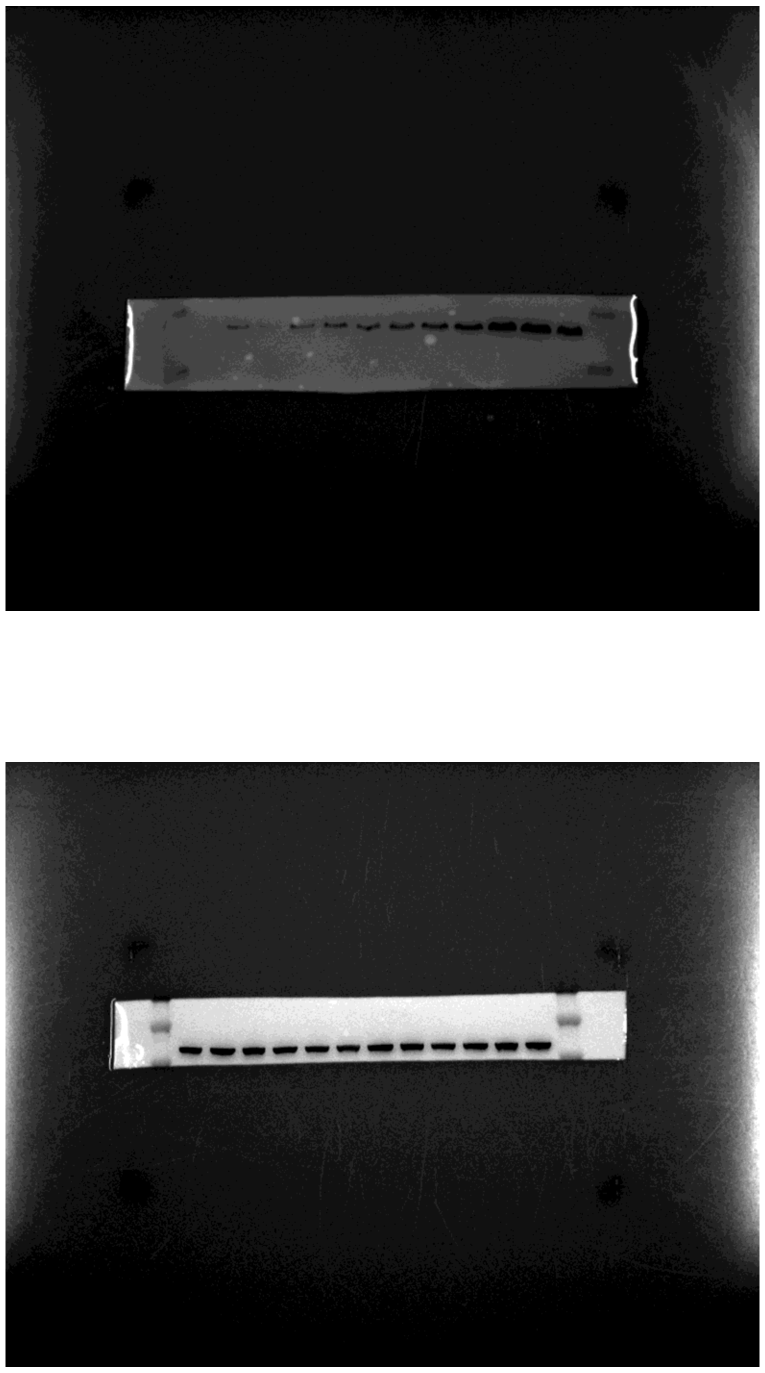

USP18

Tubulin

Figure 1 L

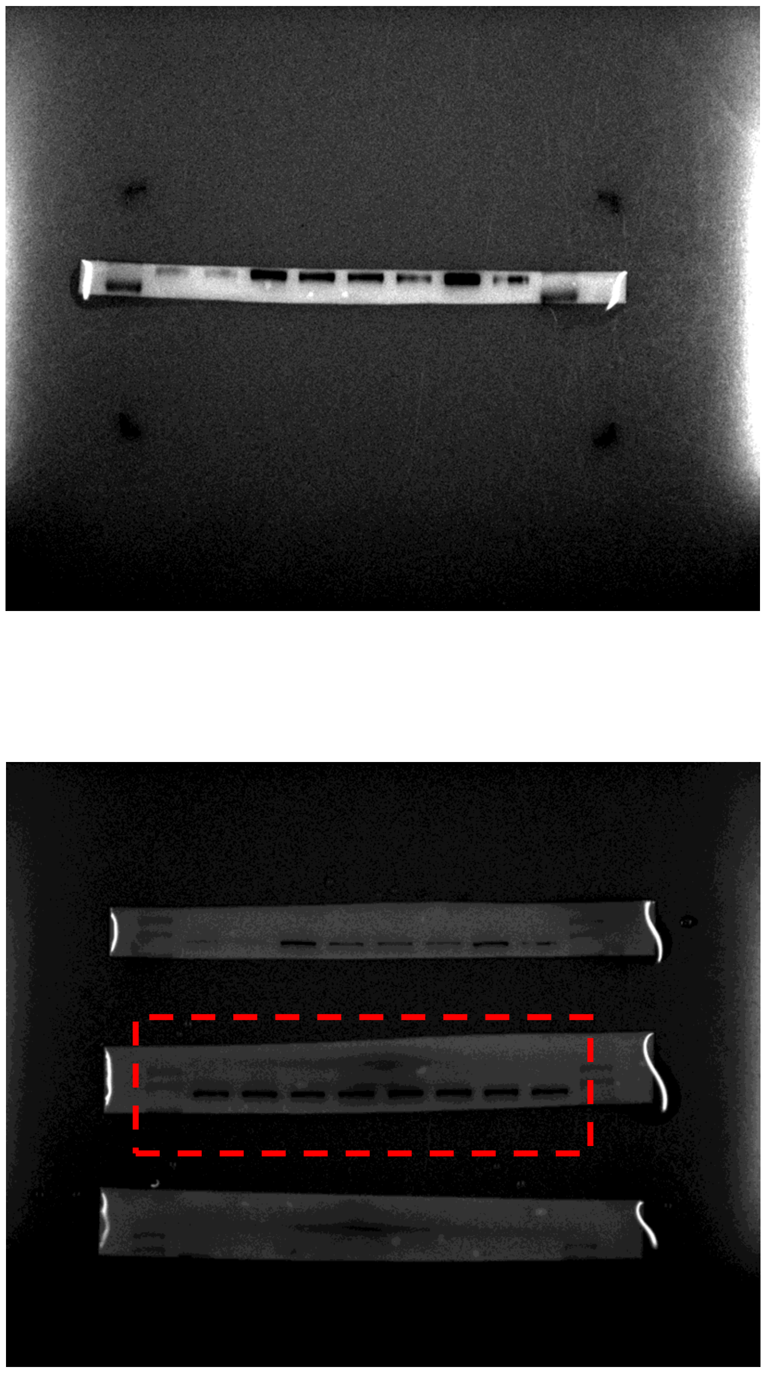

USP18

Tubulin

Figure 2 G

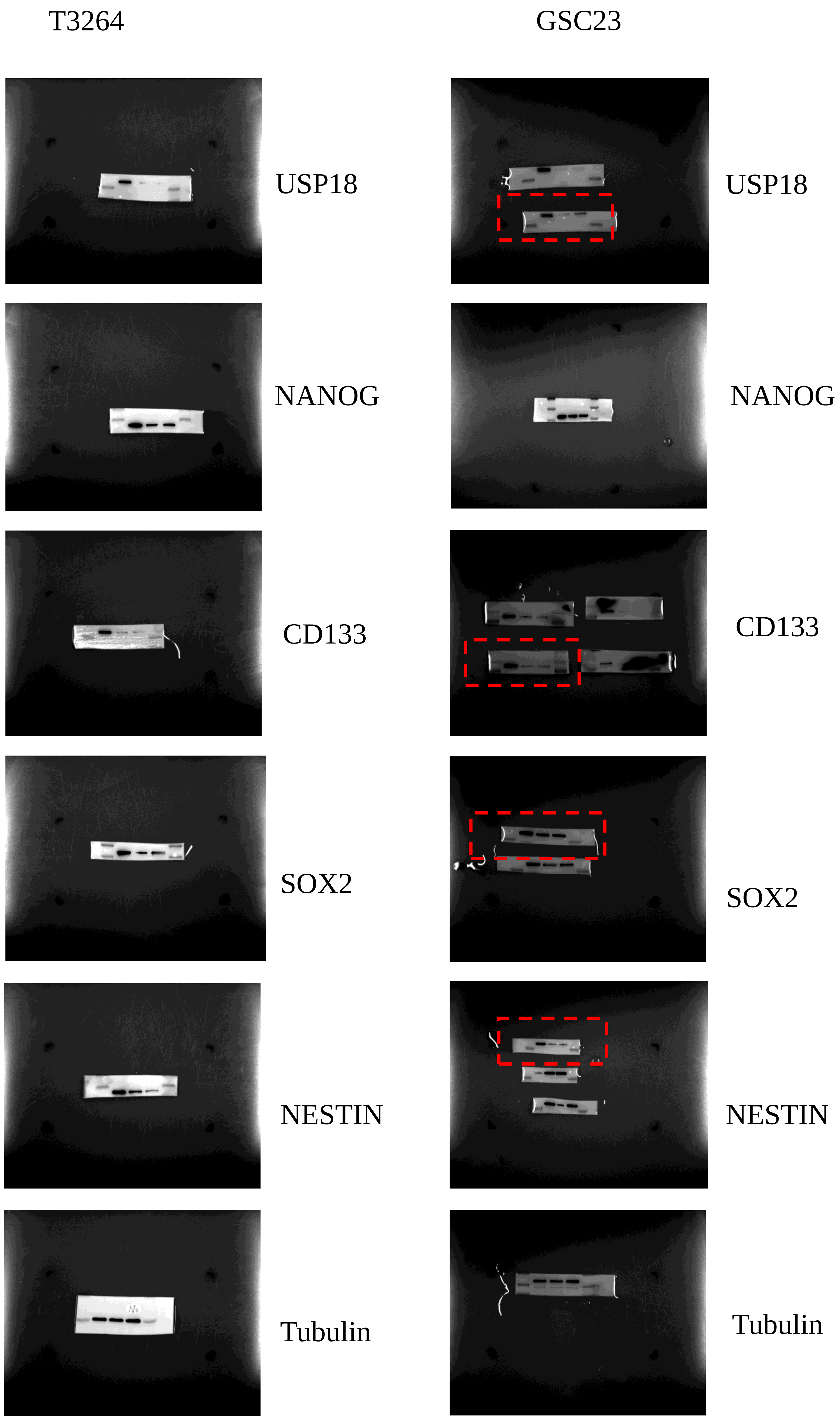
T3264
GSC23
USP18
USP18
NANOG
NANOG
CD133
CD133
SOX2
SOX2
NESTIN
NESTIN
Tubulin
Tubulin

Figure 3 G

T3264 GSC23

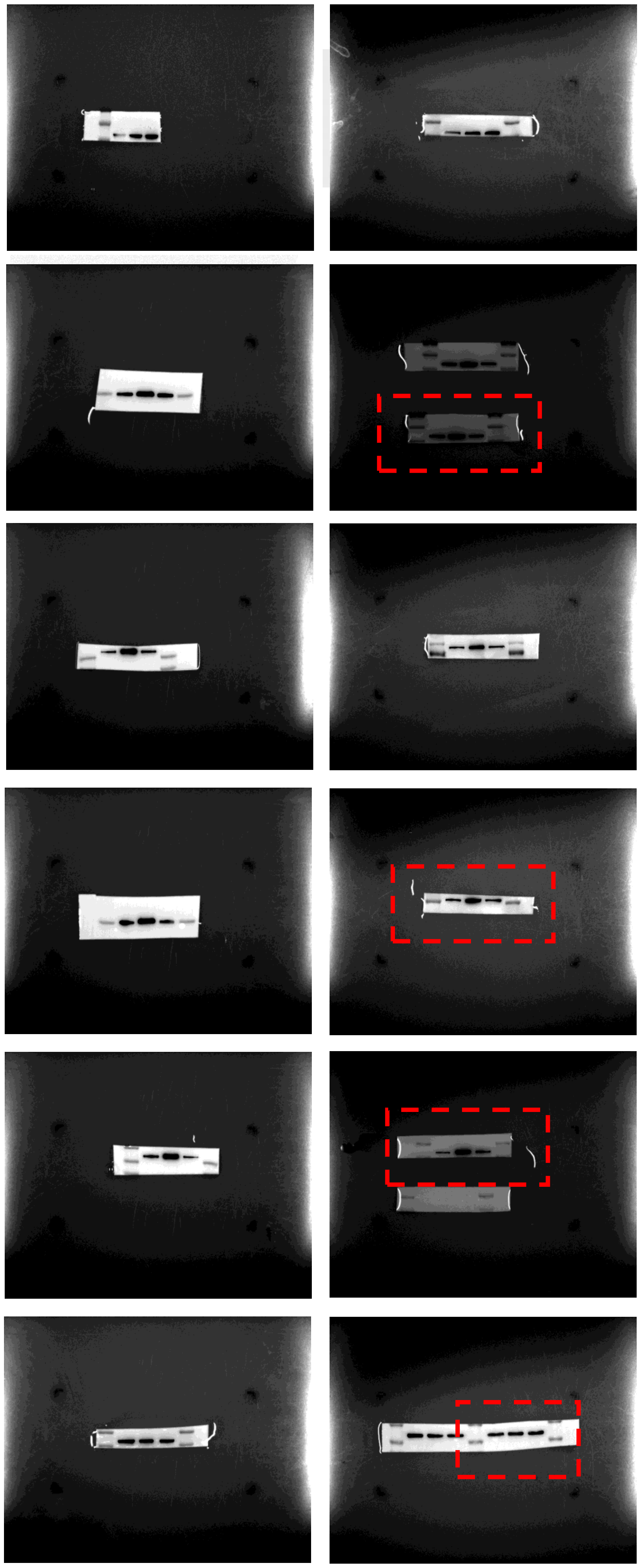

USP18

NANOG

CD133

SOX2

NESTIN

Tubulin

Figure 4 C

U87 LN229

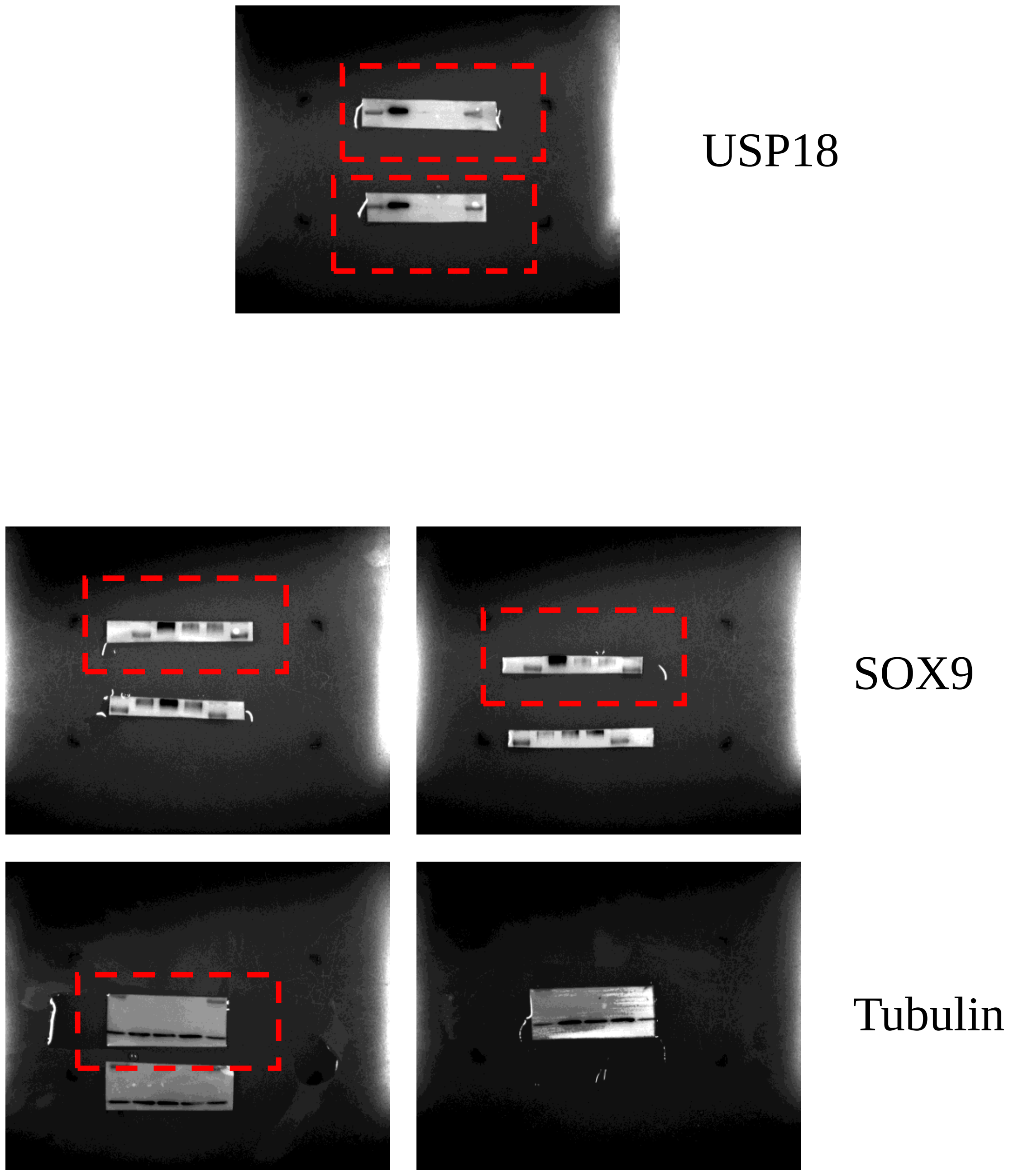

USP18

SOX9

Tubulin

Figure 4 D

A172

U251

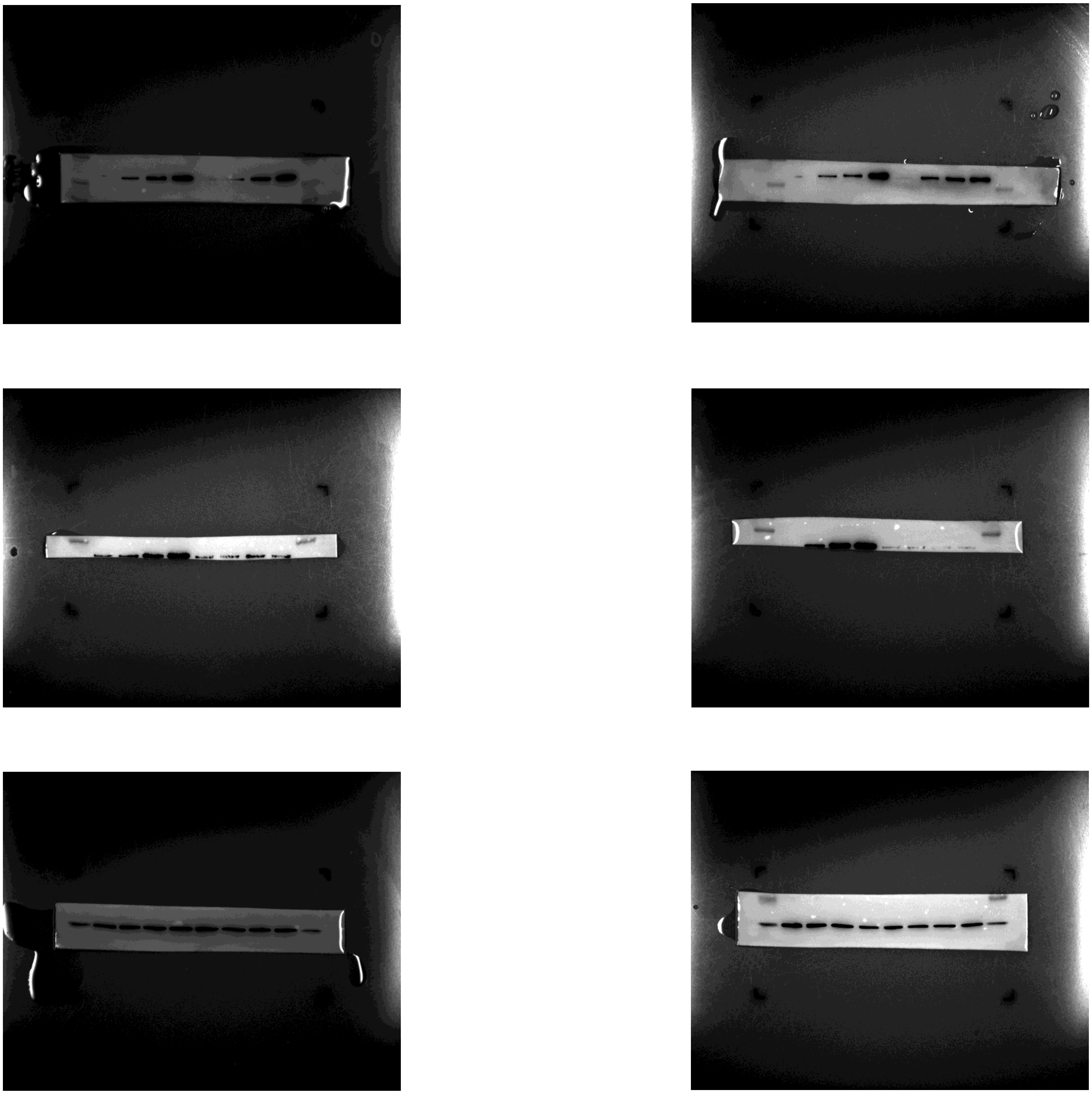

USP18

SOX9

Tubulin

Figure 4 E

U87

U251

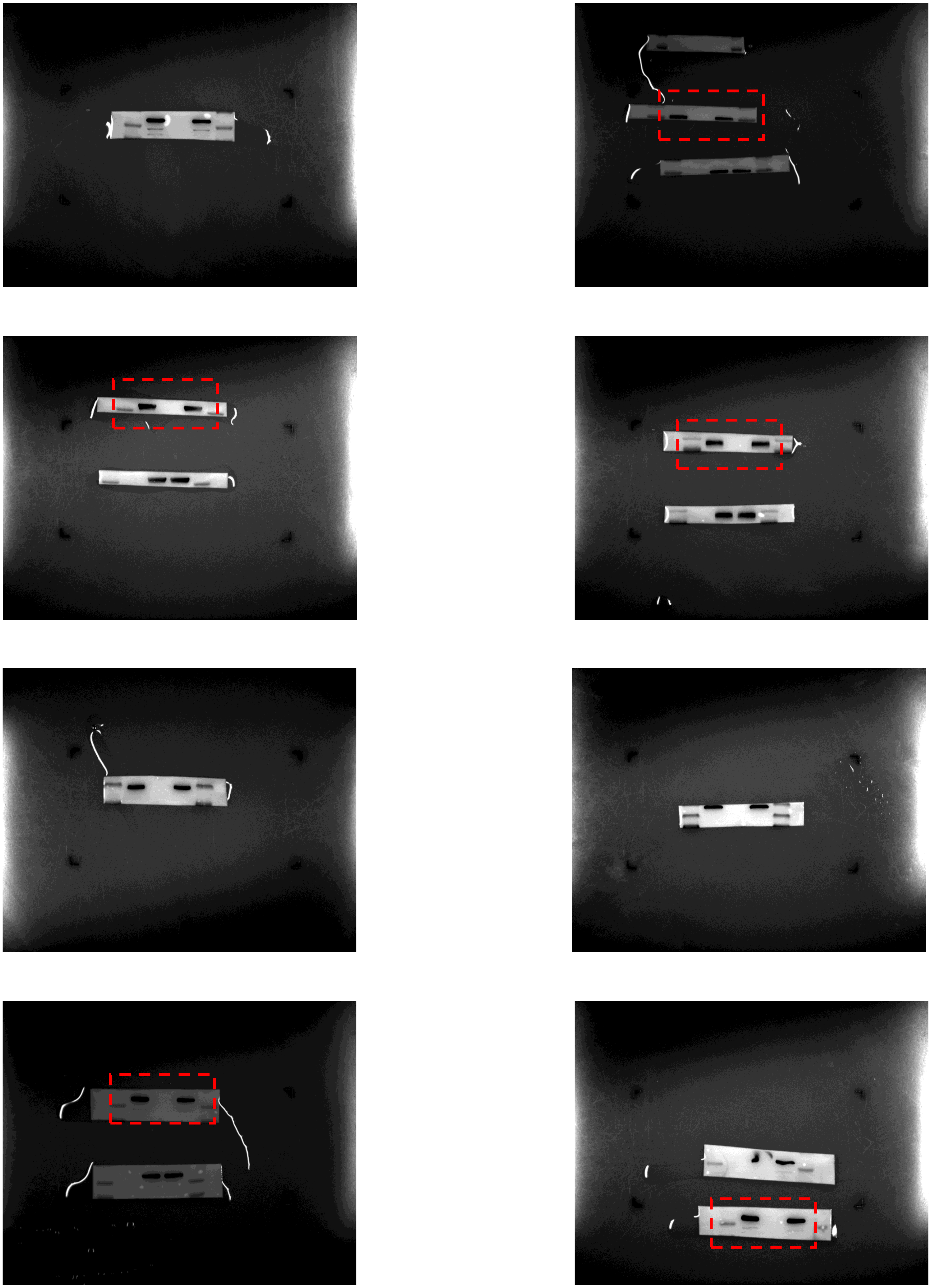

SOX9

USP18

USP18

SOX9

Figure 4 F

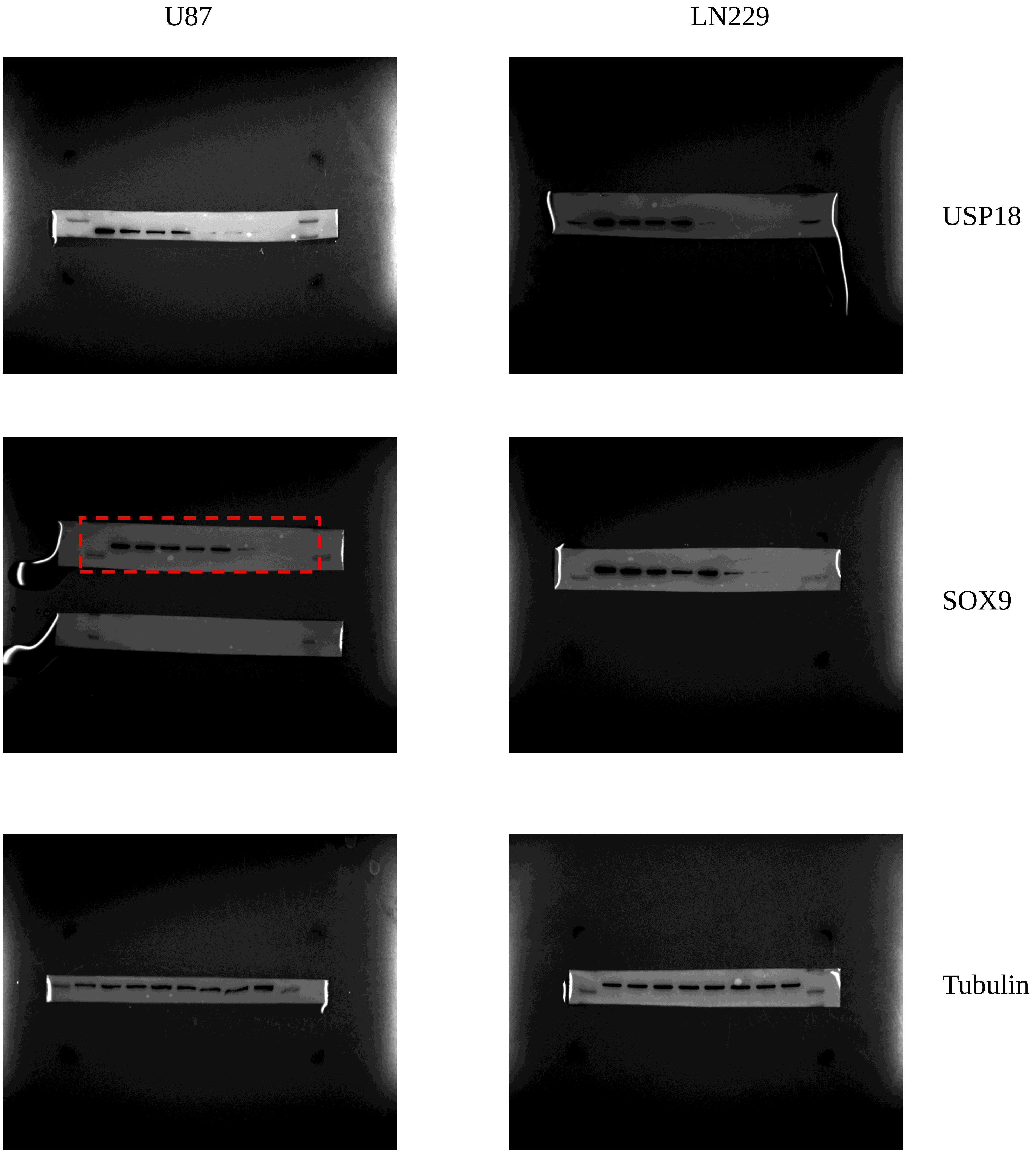

Figure 4 G

U251

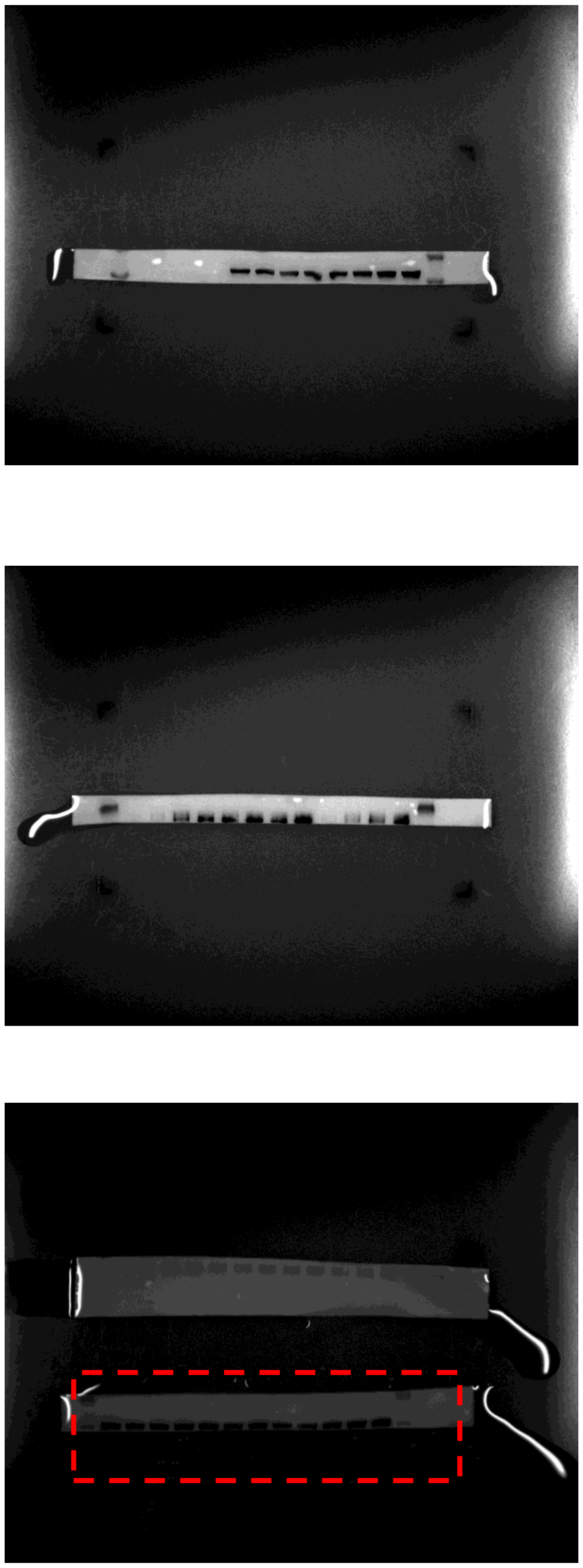

Flag

SOX9

Tubulin

Figure 4 H

U87 LN229

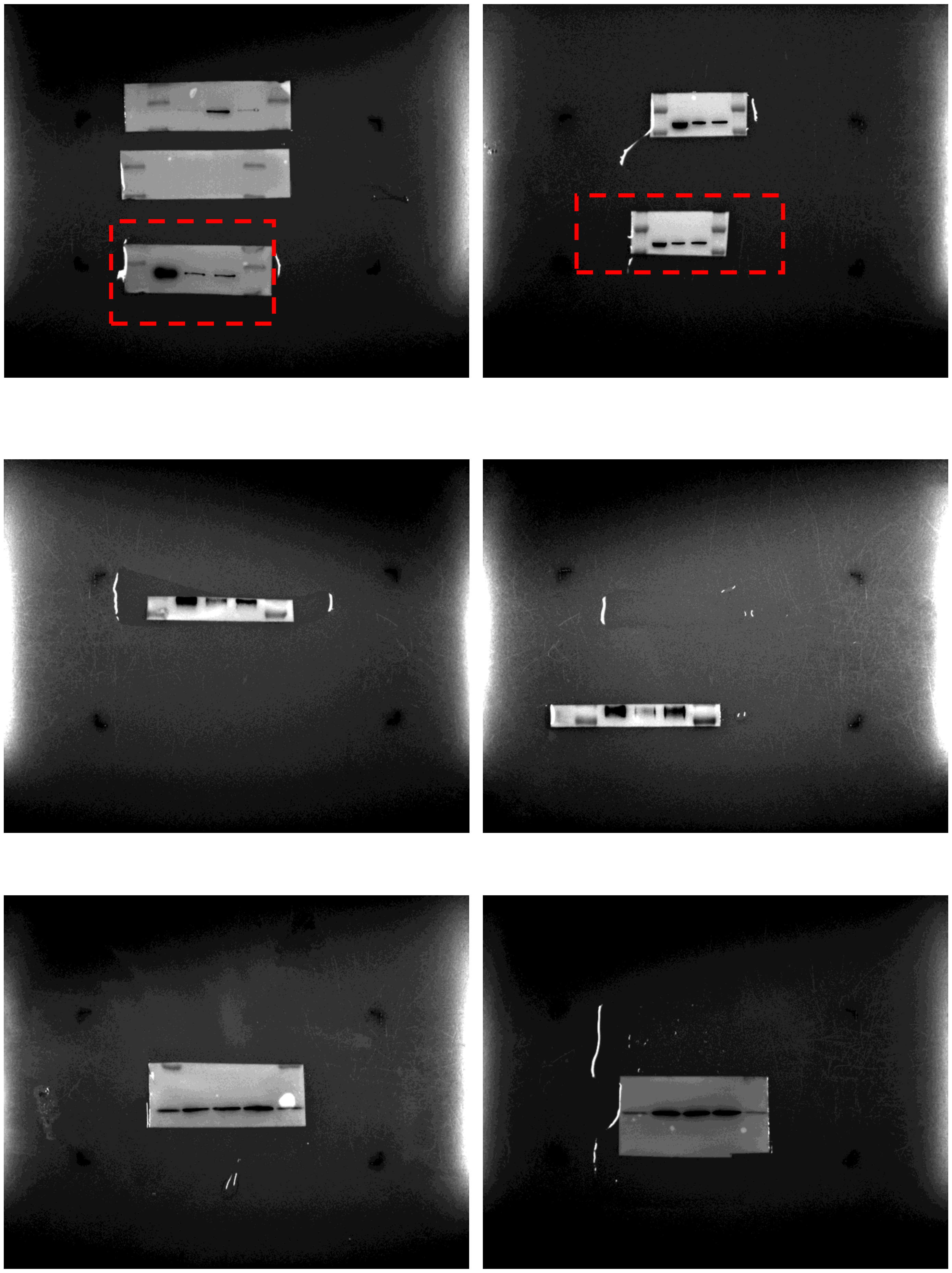

USP18

SOX9

Tubulin

Figure 4 I

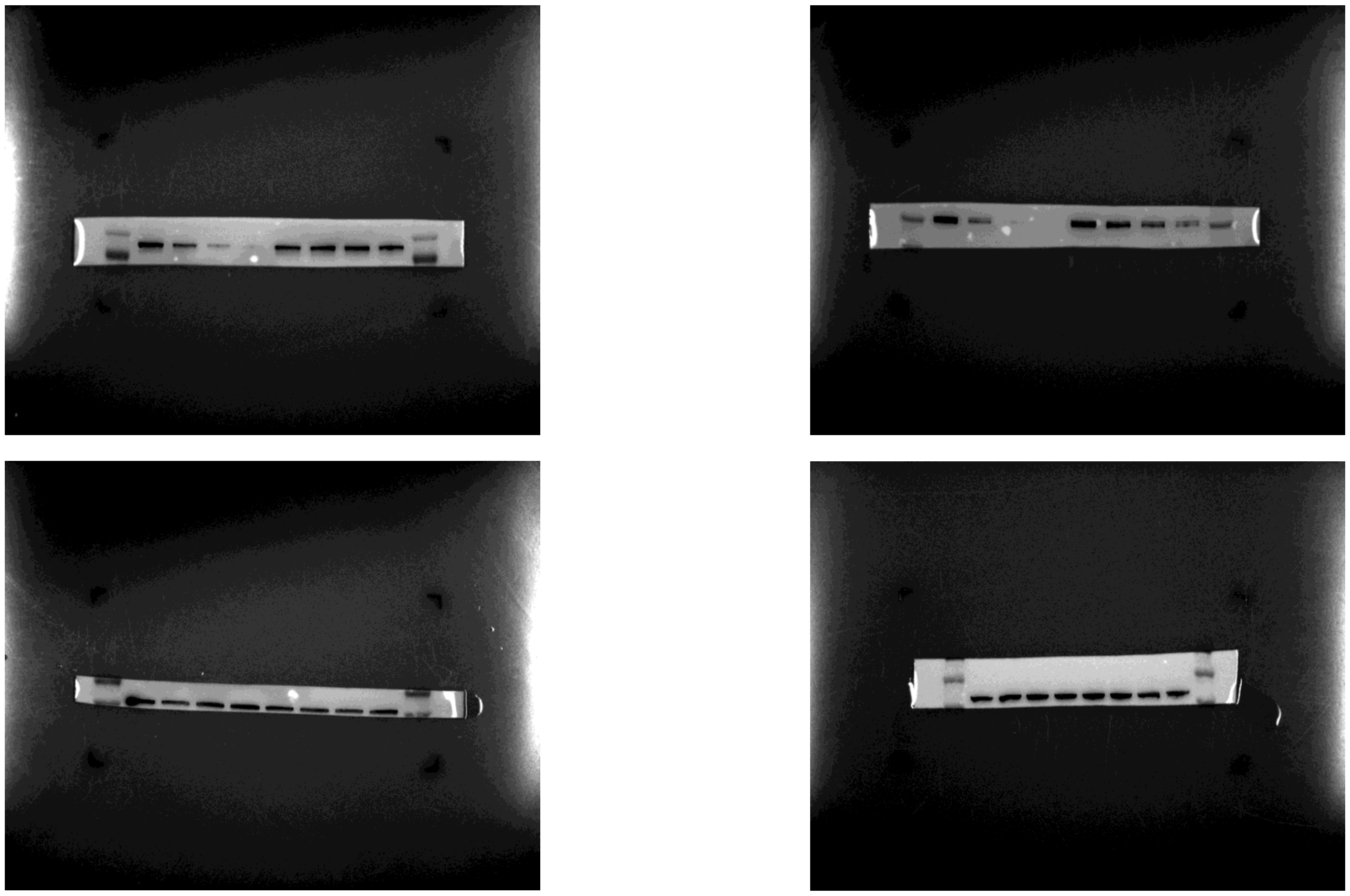

Figure 4 K

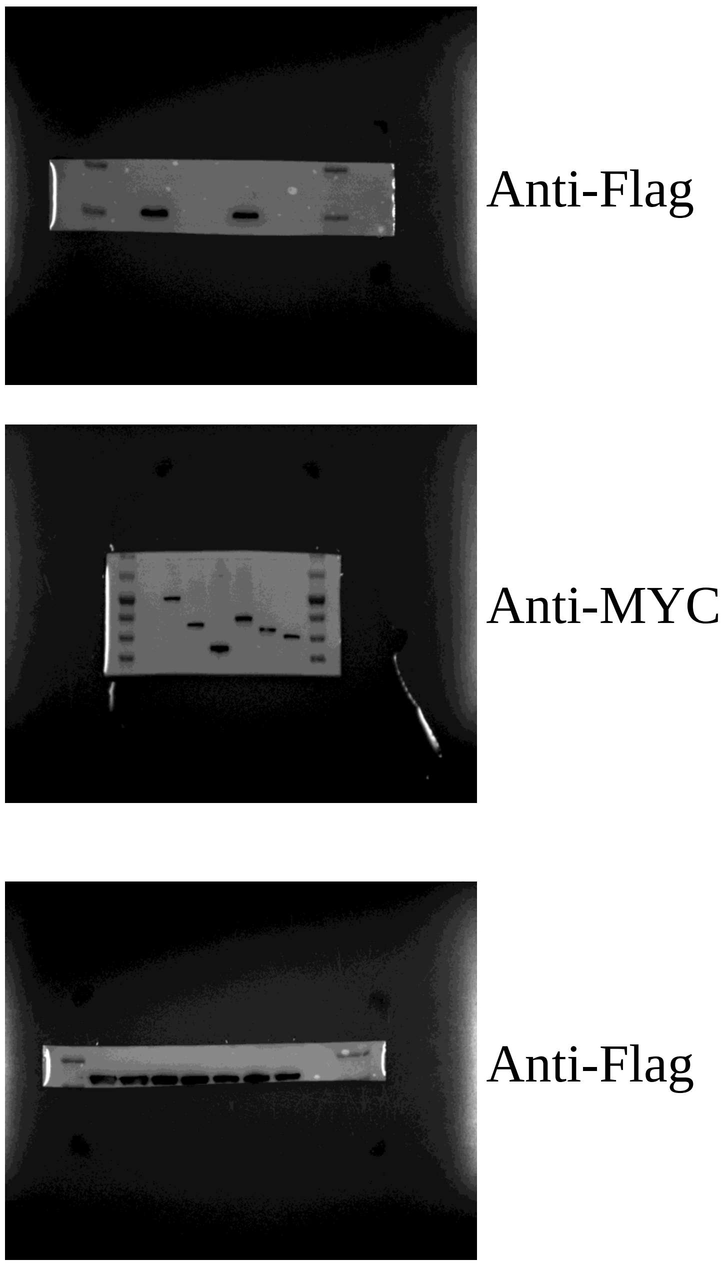

Figure 4 L

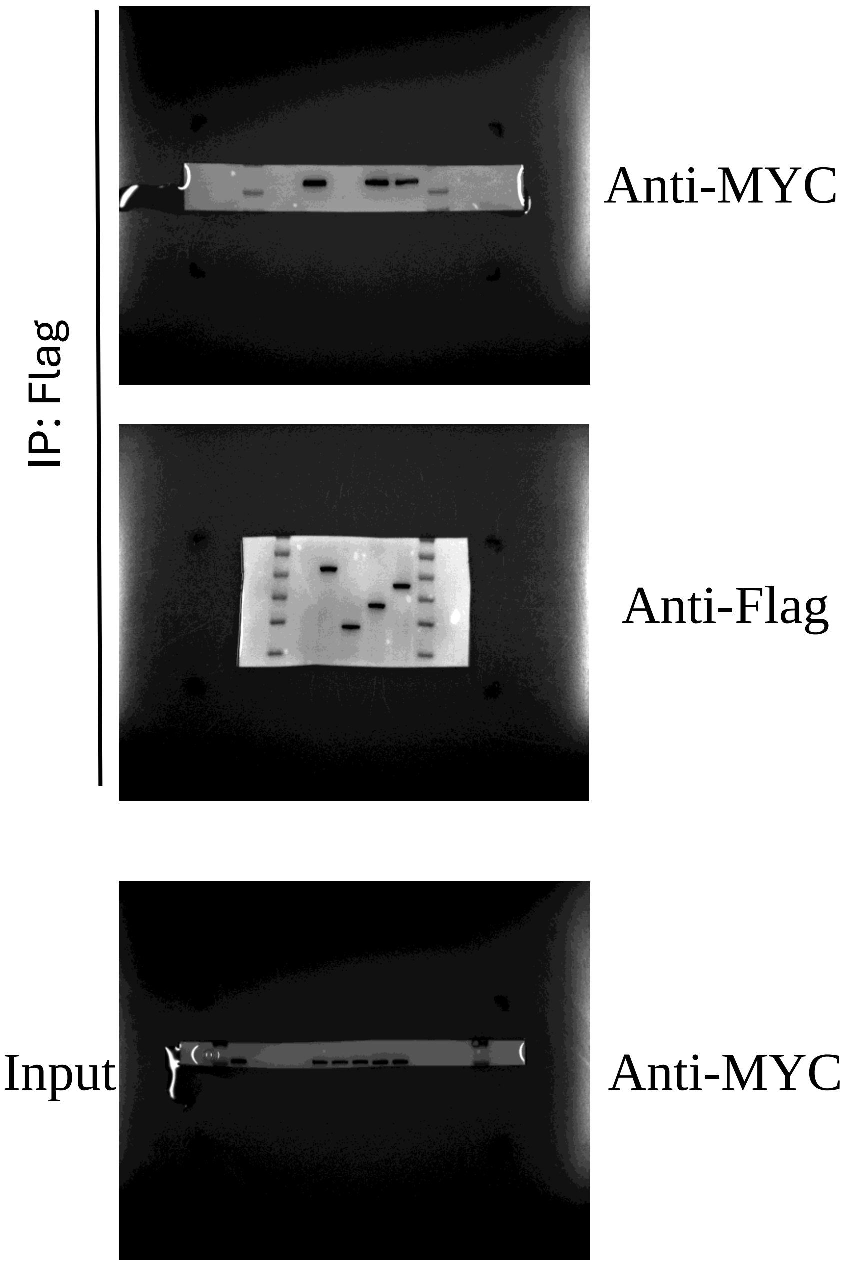

Figure 5 A

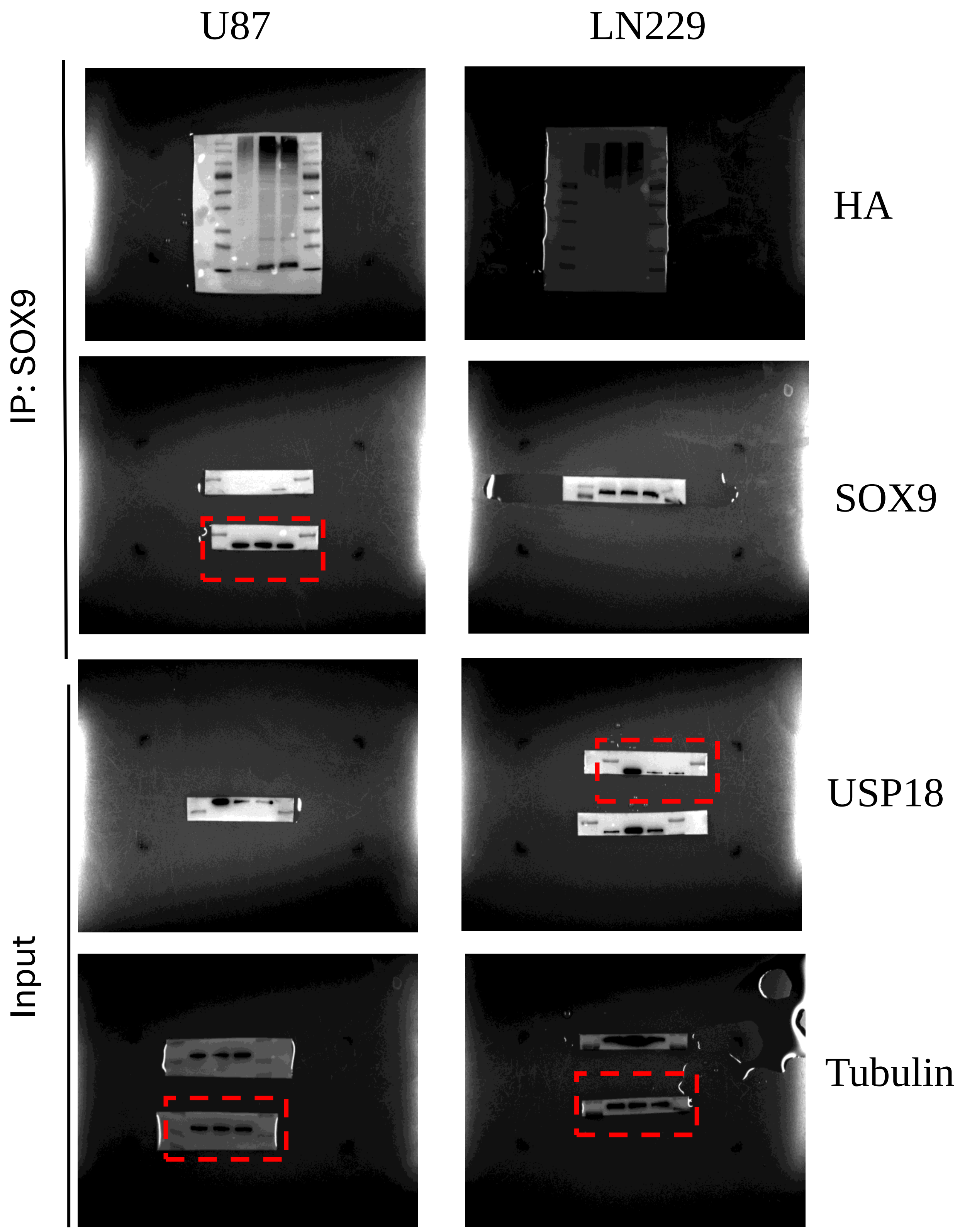

Figure 5 B

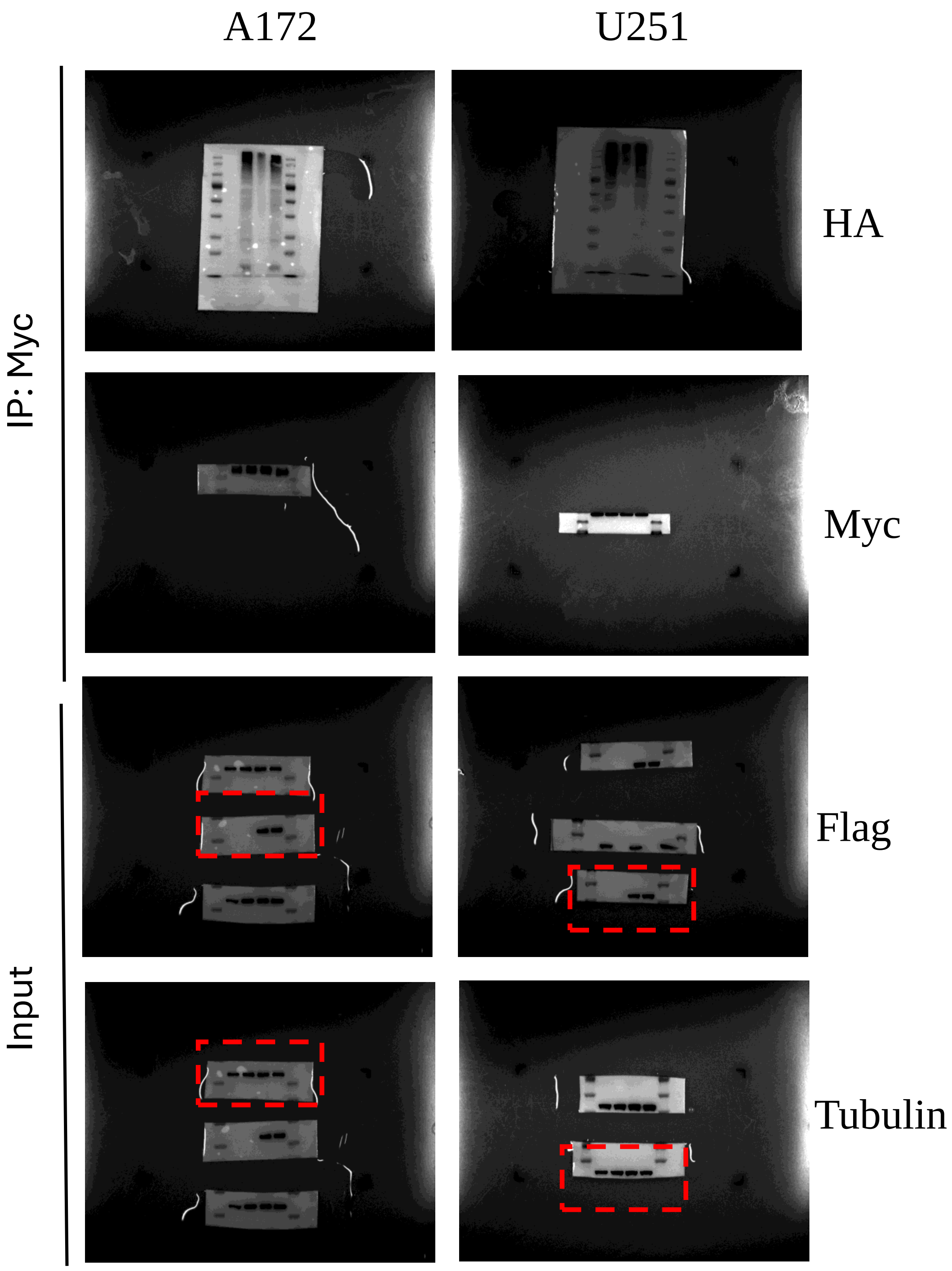

Figure 5 C

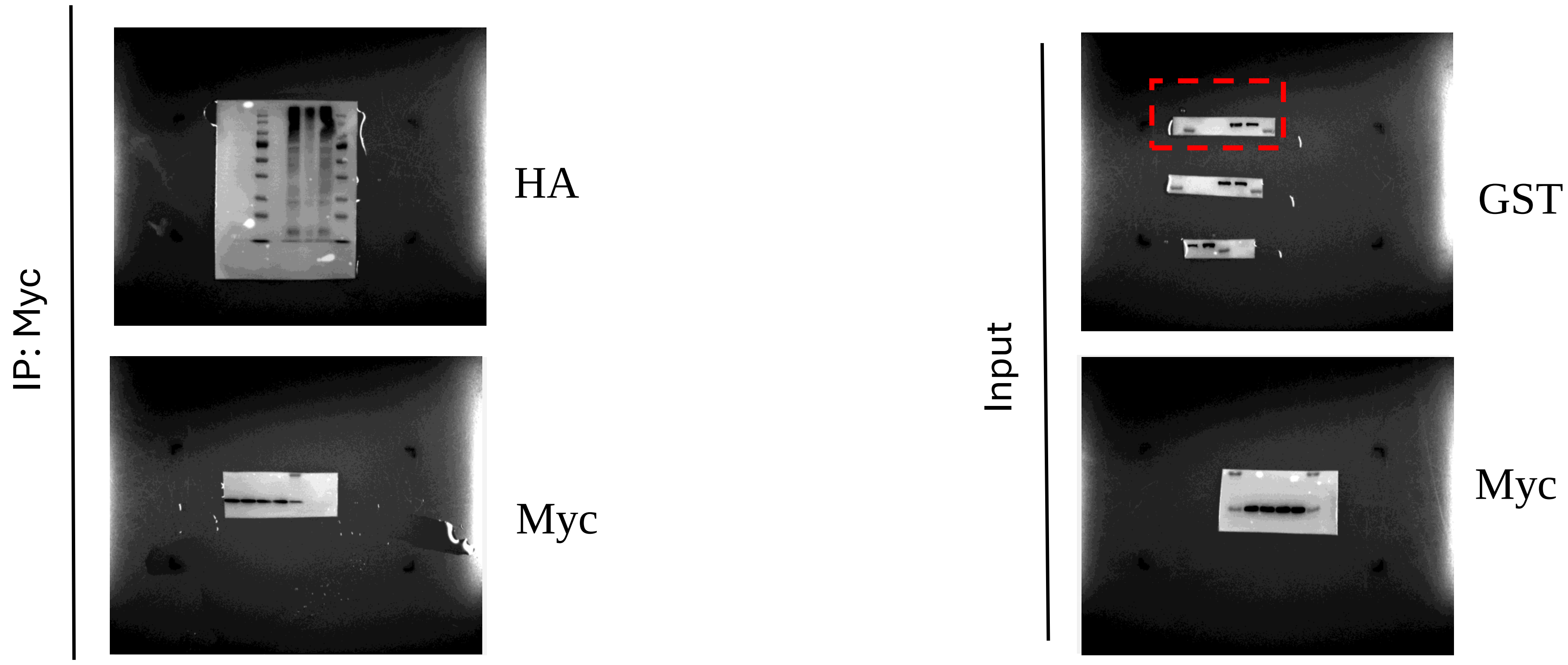

Figure 5 D

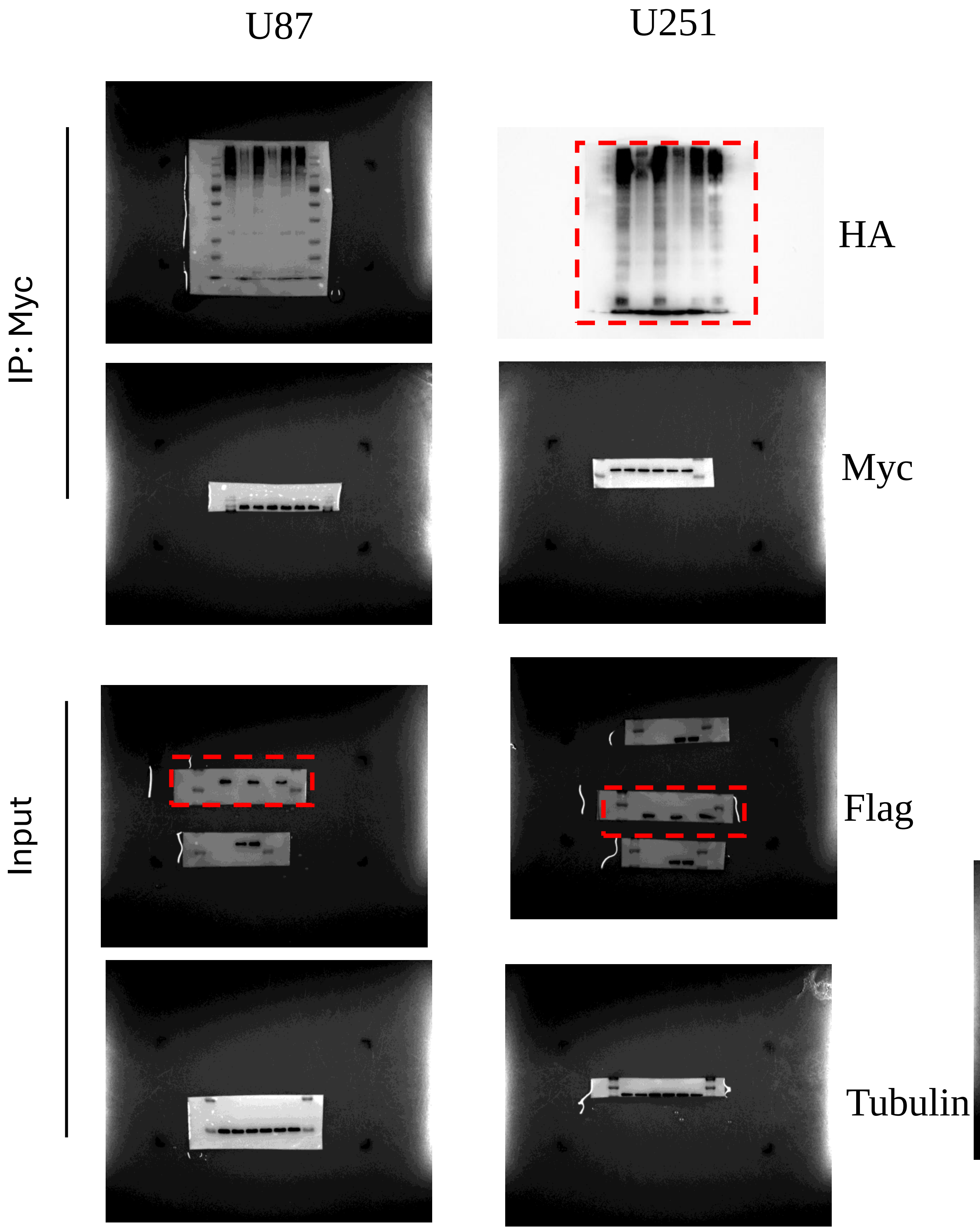

Figure 5 E

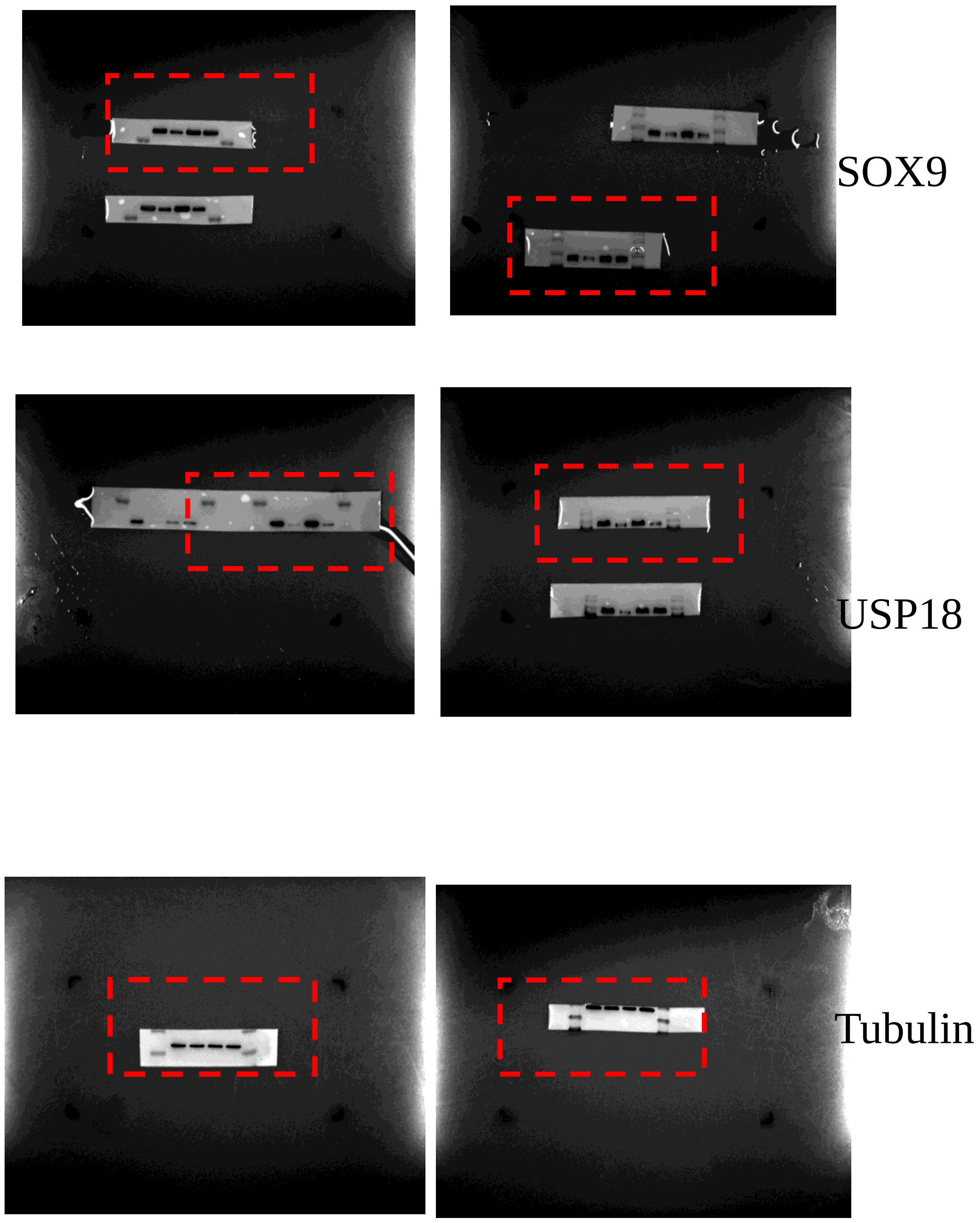

Figure 5 F

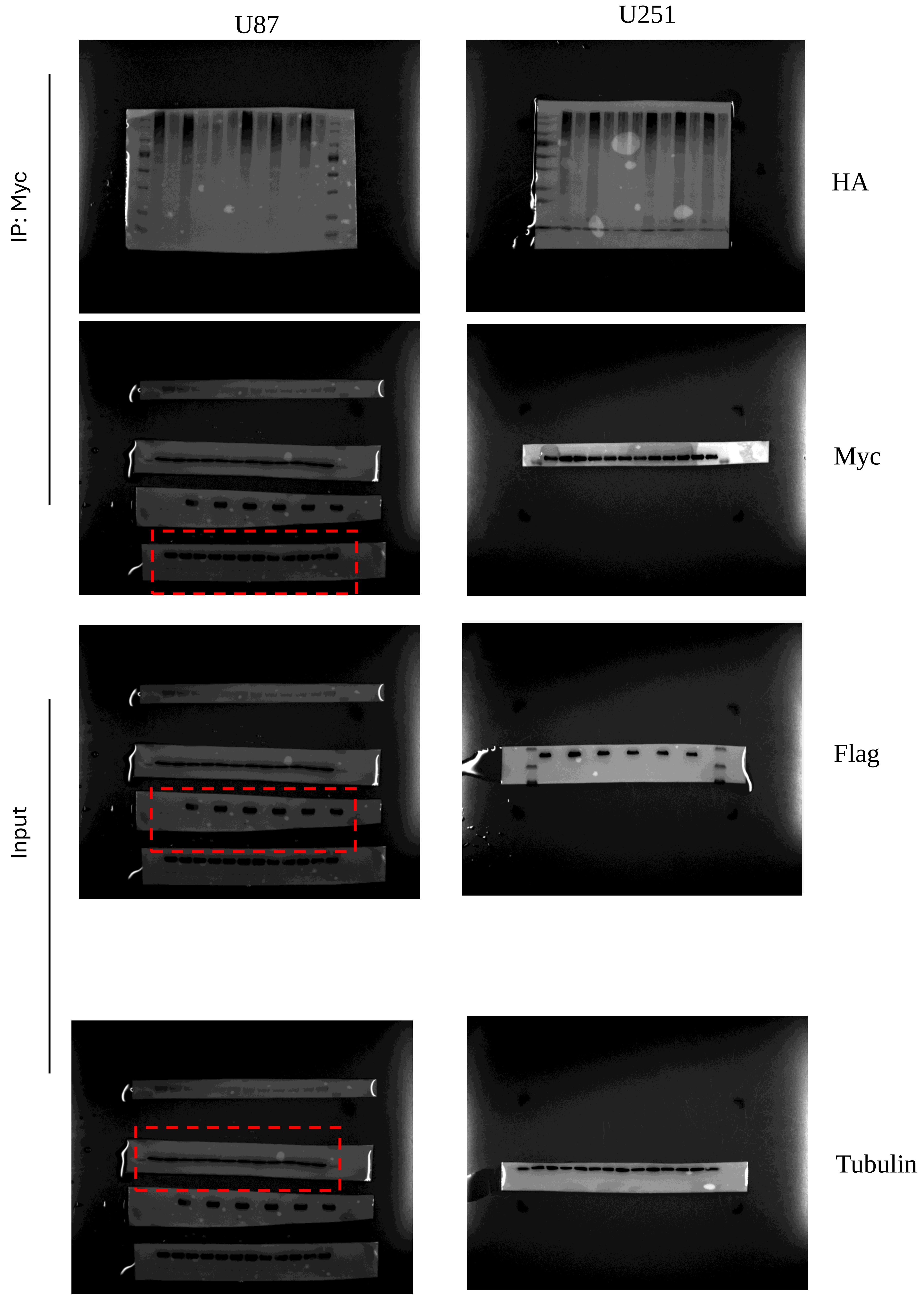

Figure 6 A

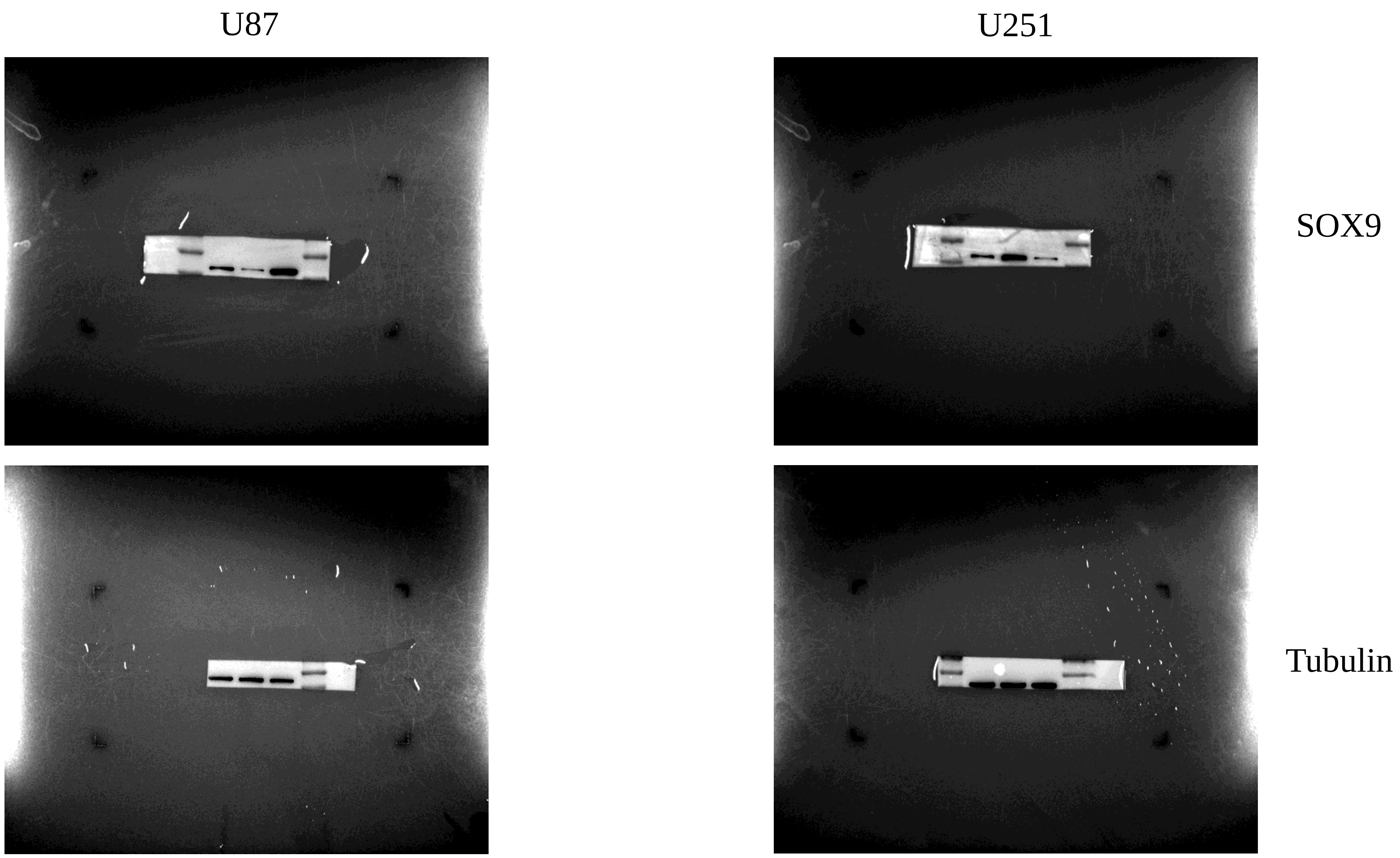

Figure 6 G

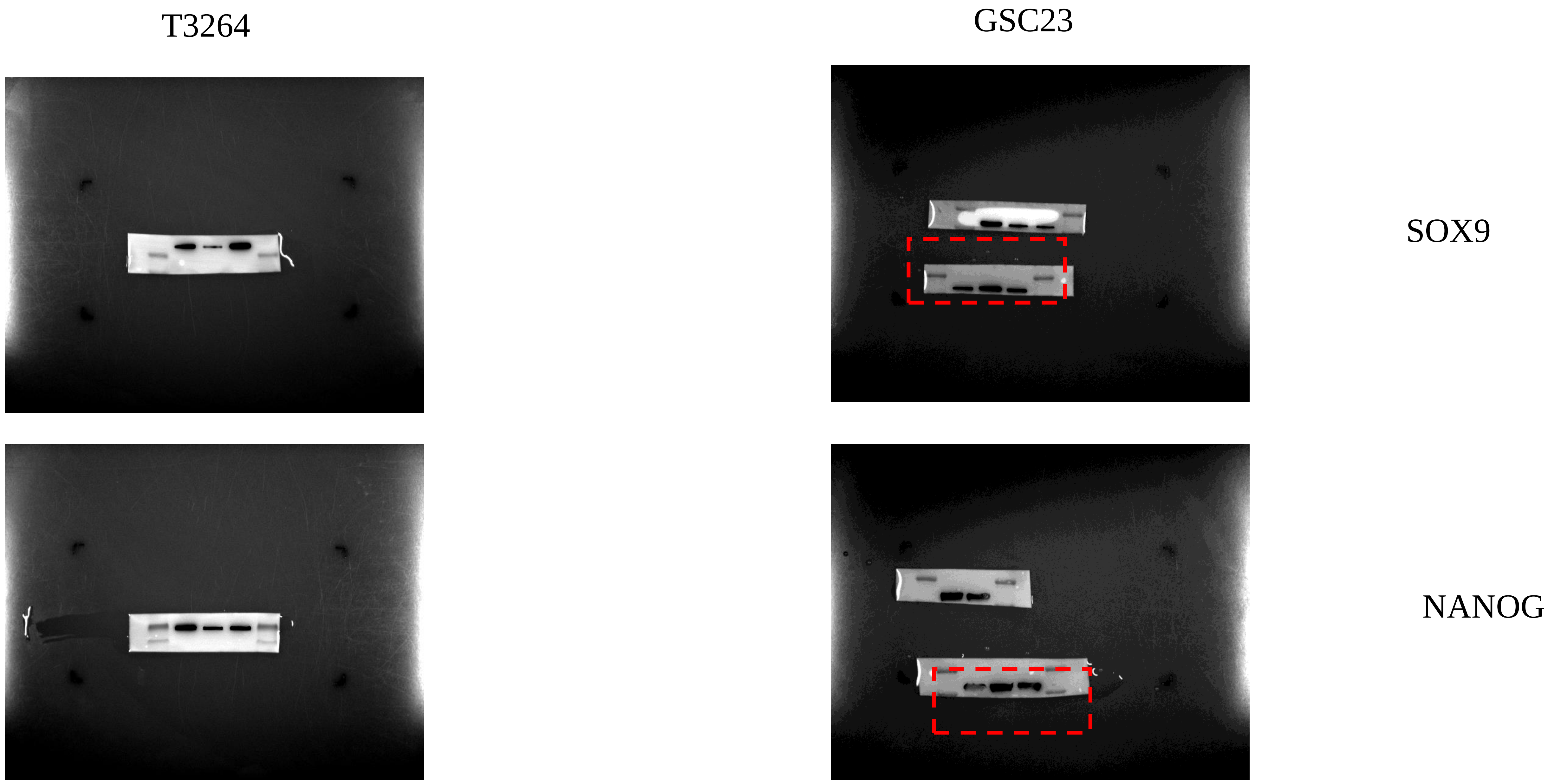

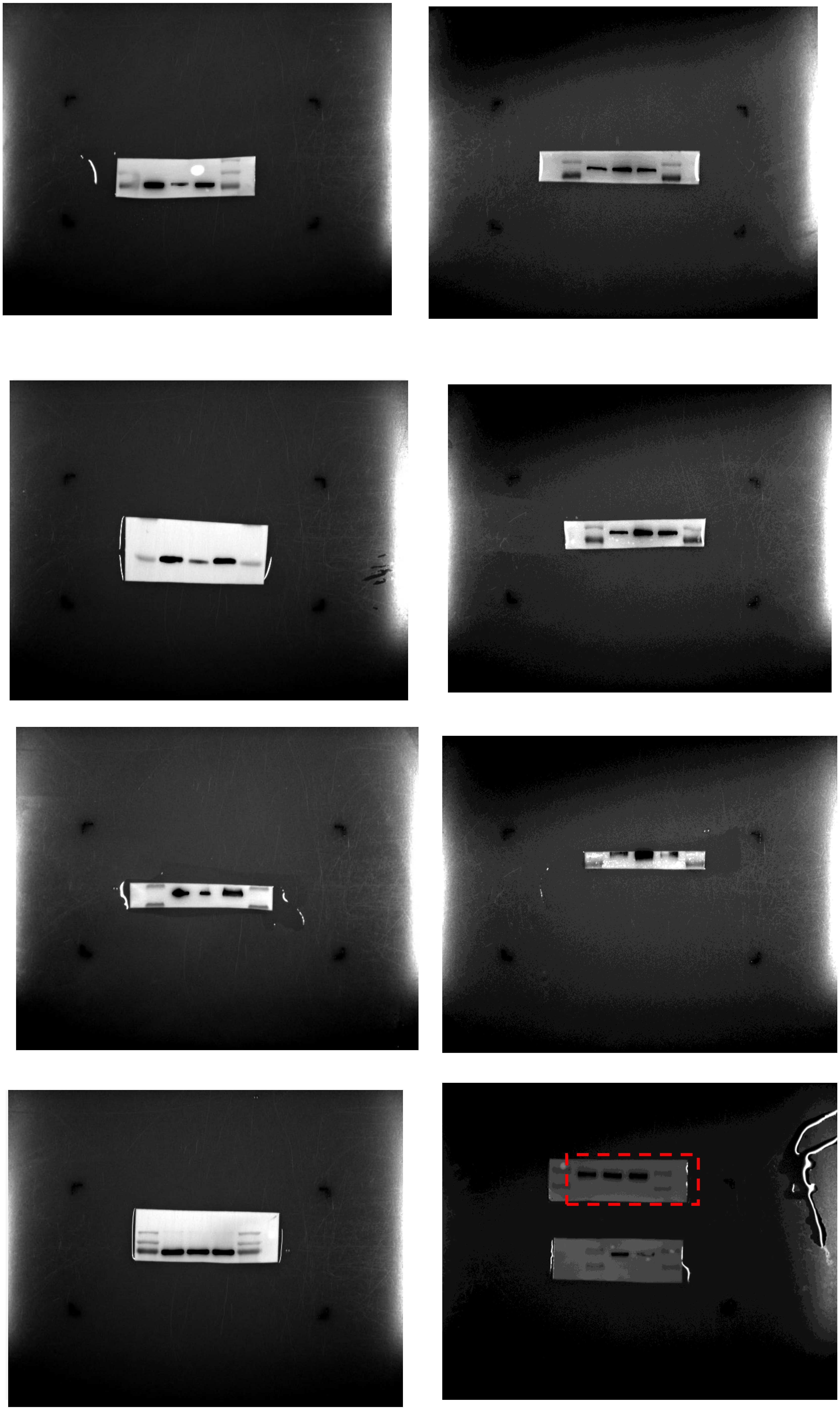

CD133

SOX2

NESTIN

Tubulin

Figure 8 D

U87 U251

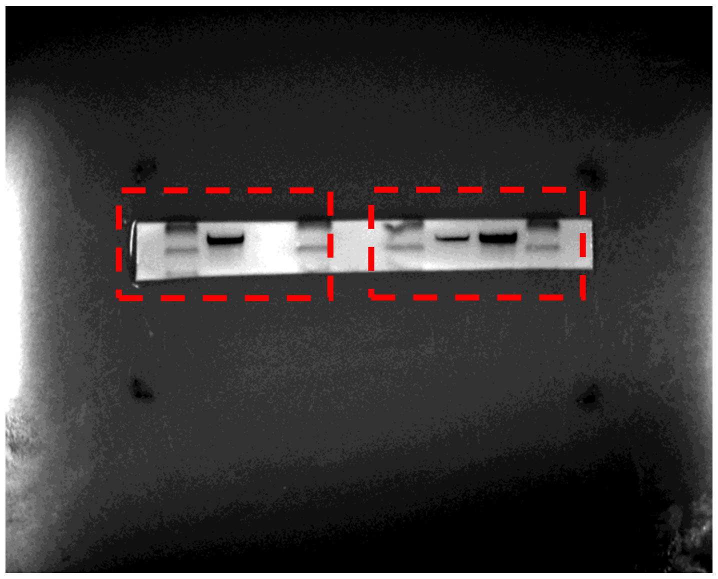

YY1

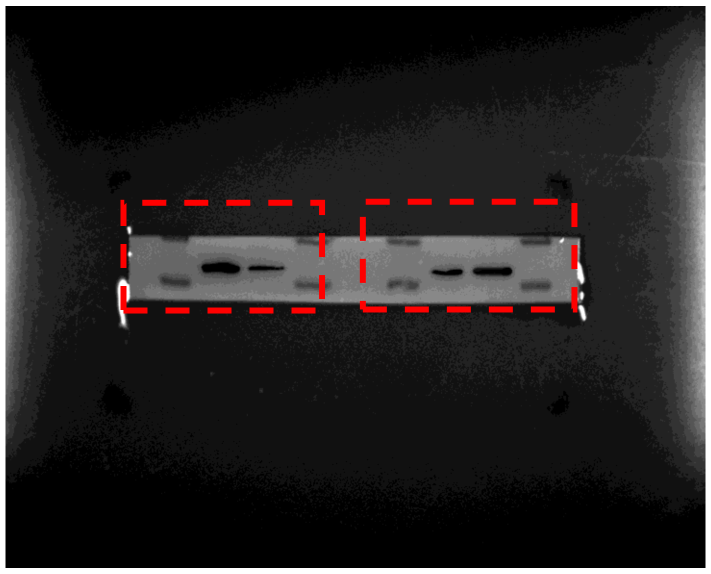

USP18

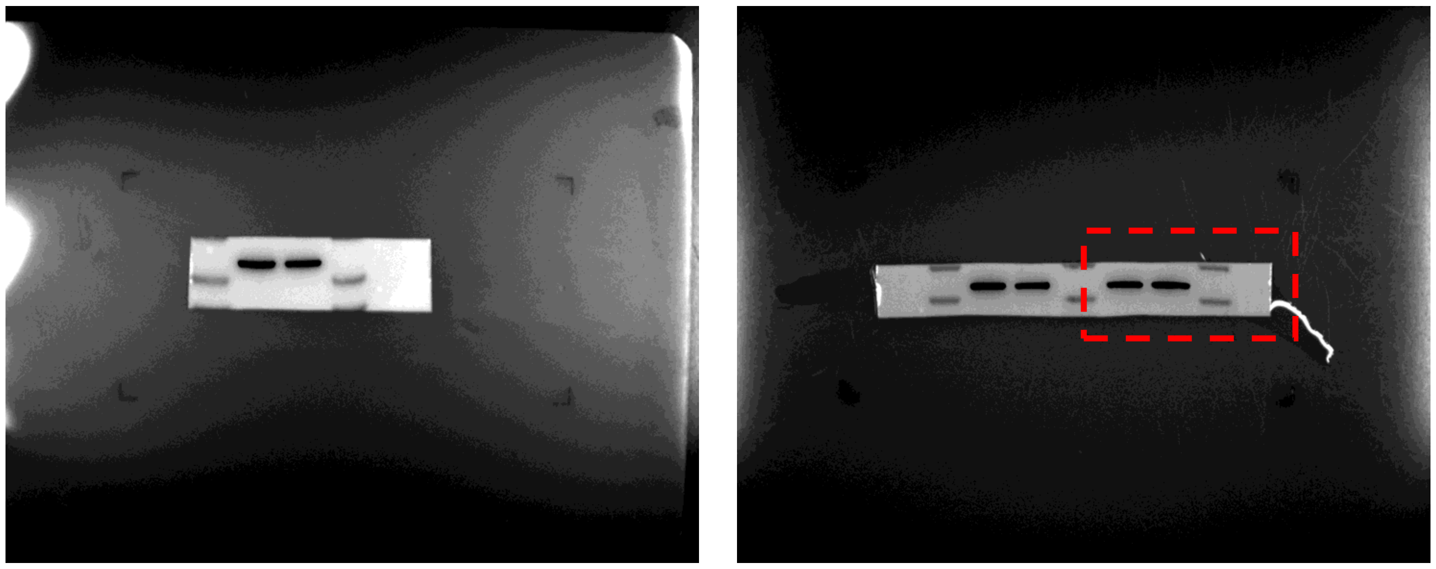

Tubulin

Figure S2 A

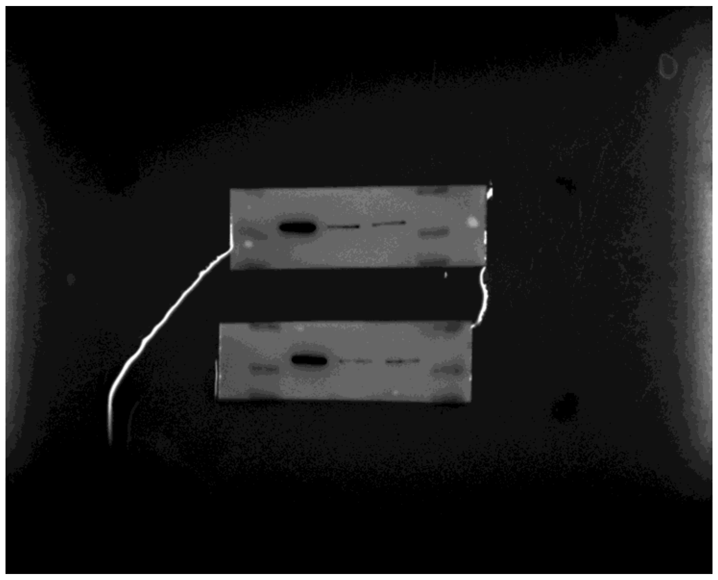

USP18

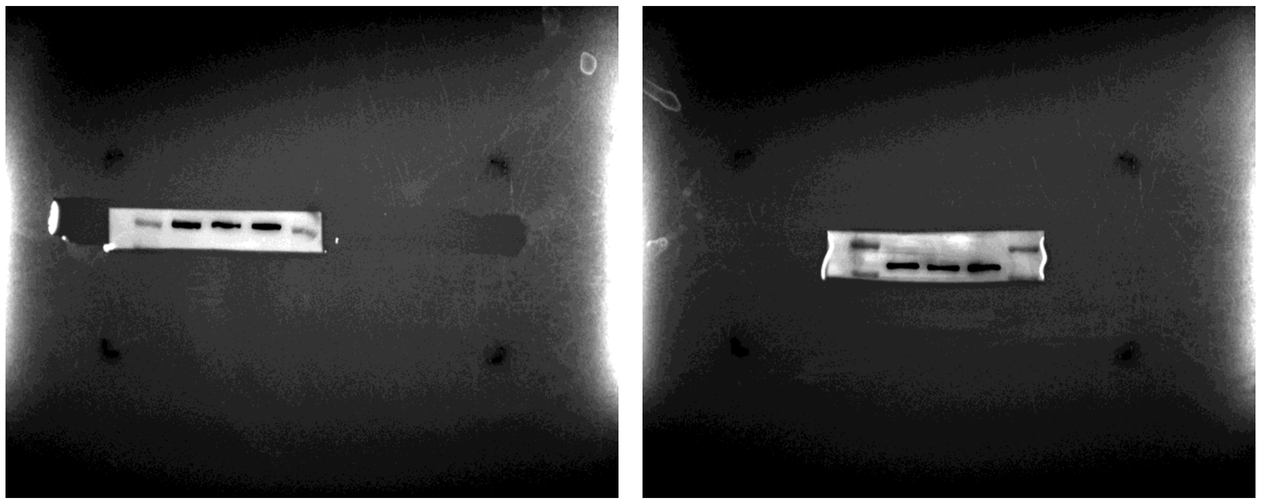

Tubulin

Figure S3 A

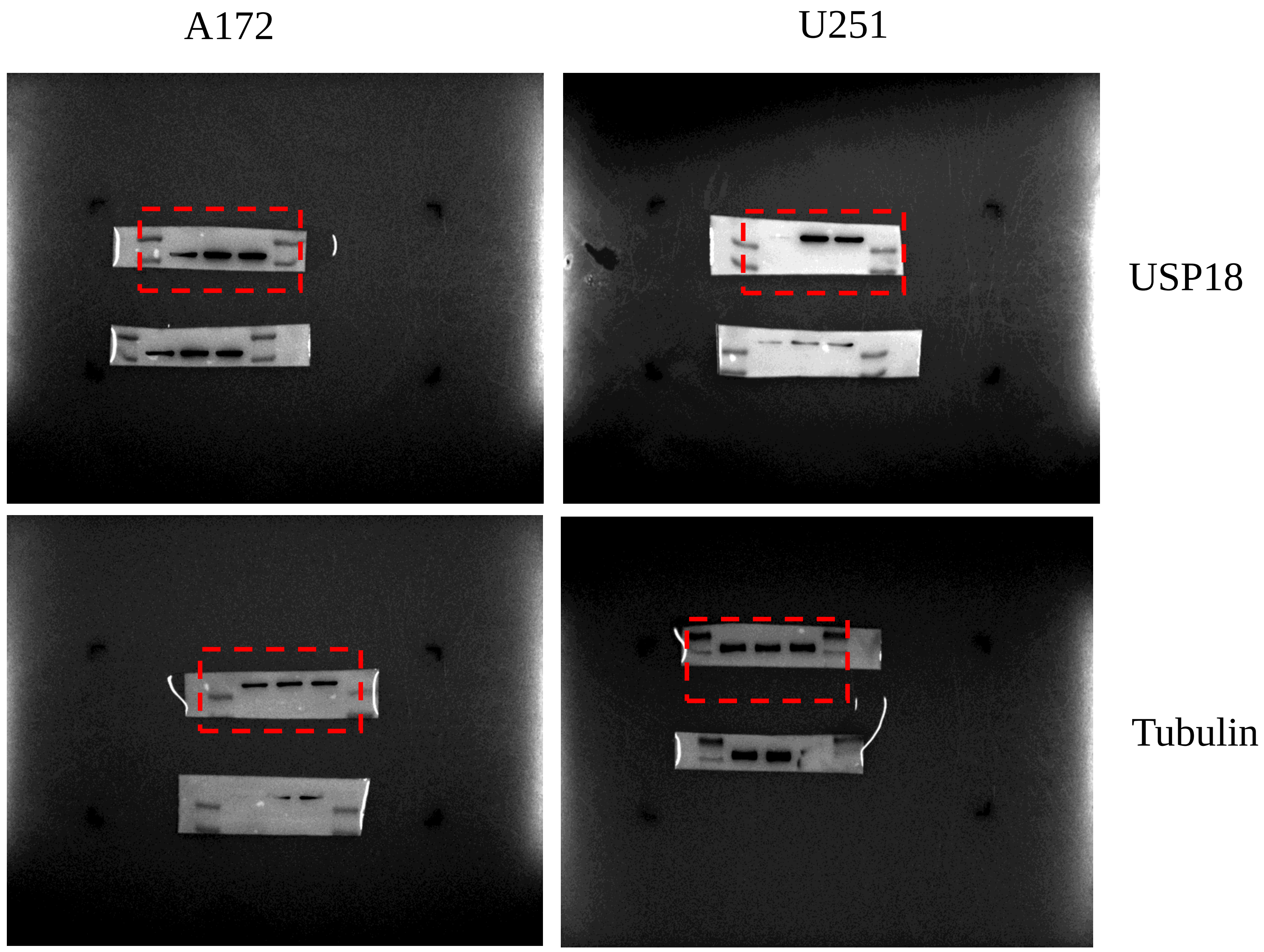

Figure S7 D

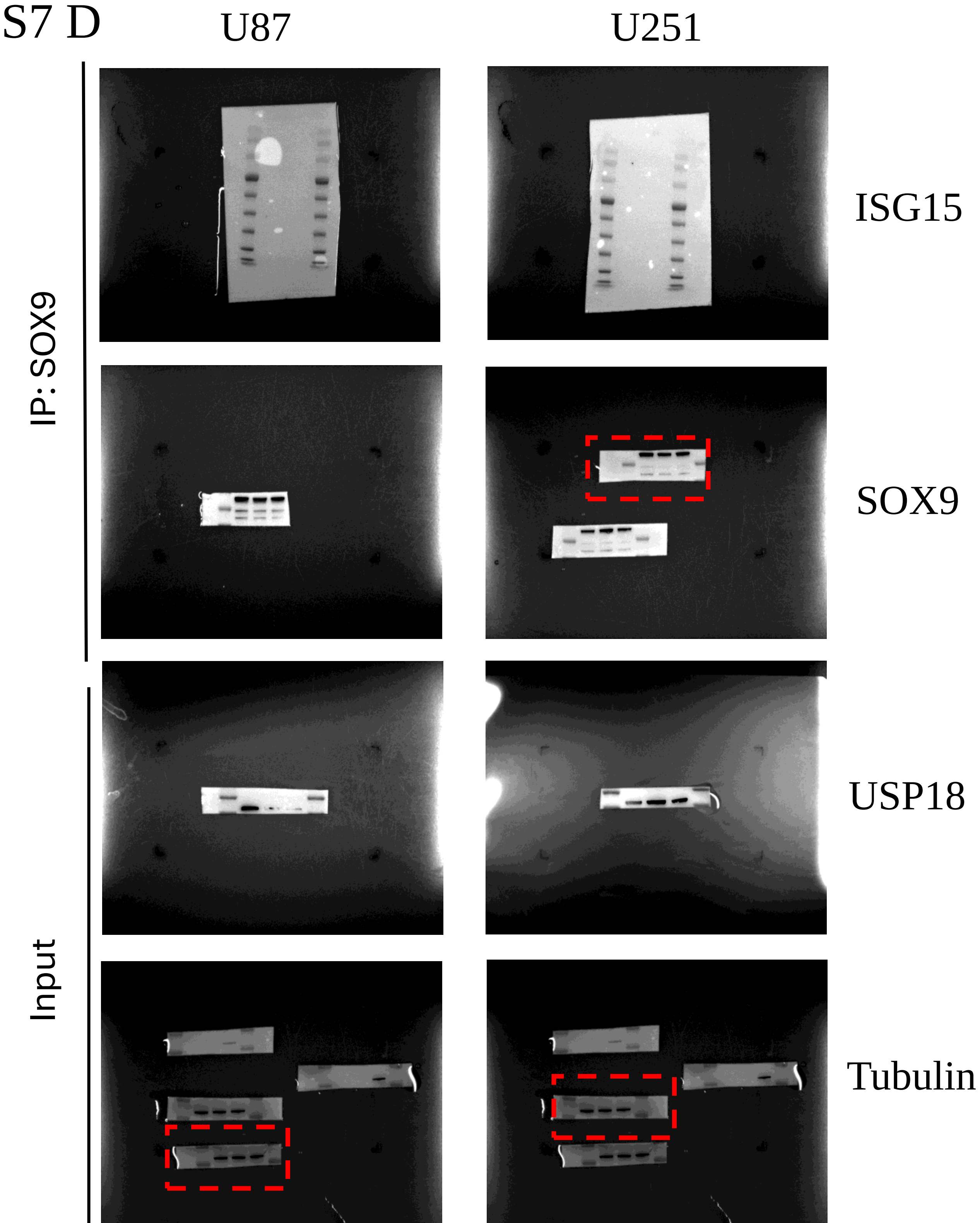

Figure S8 A

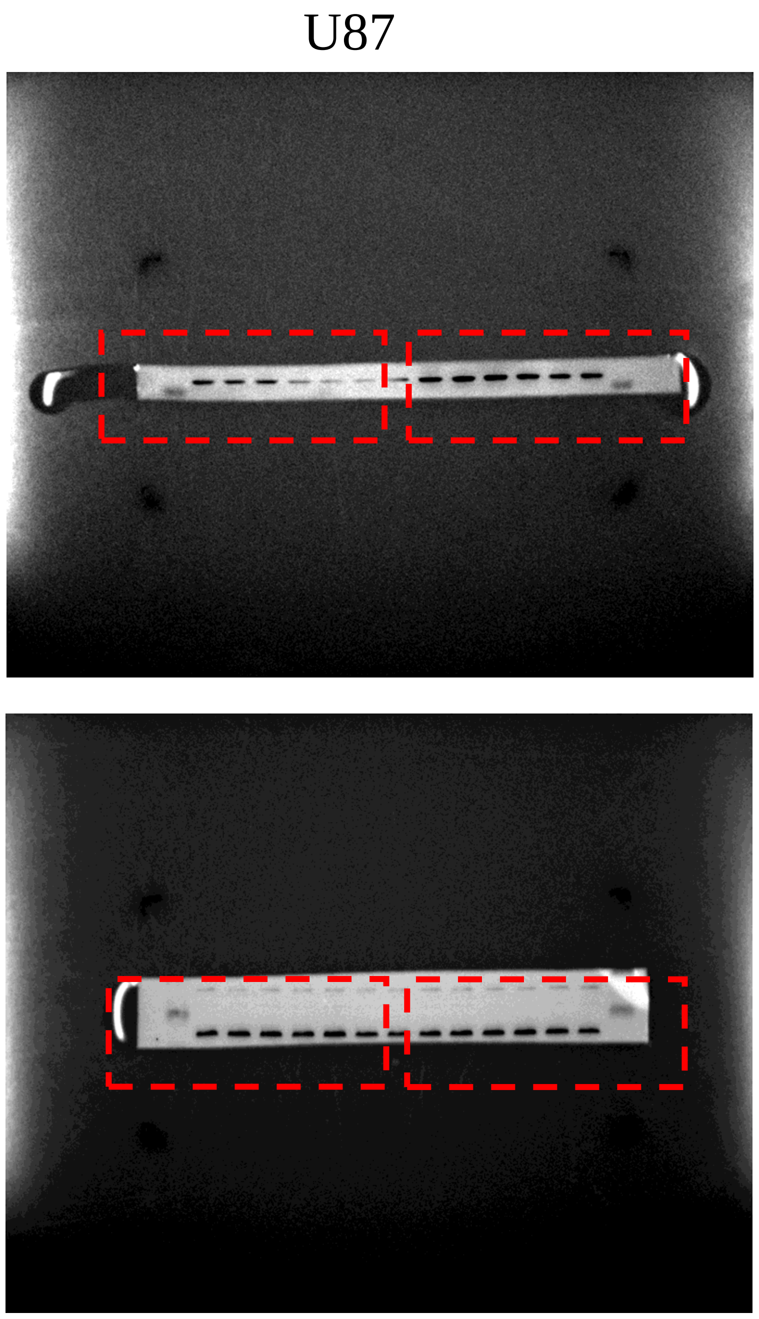

Figure S4 A

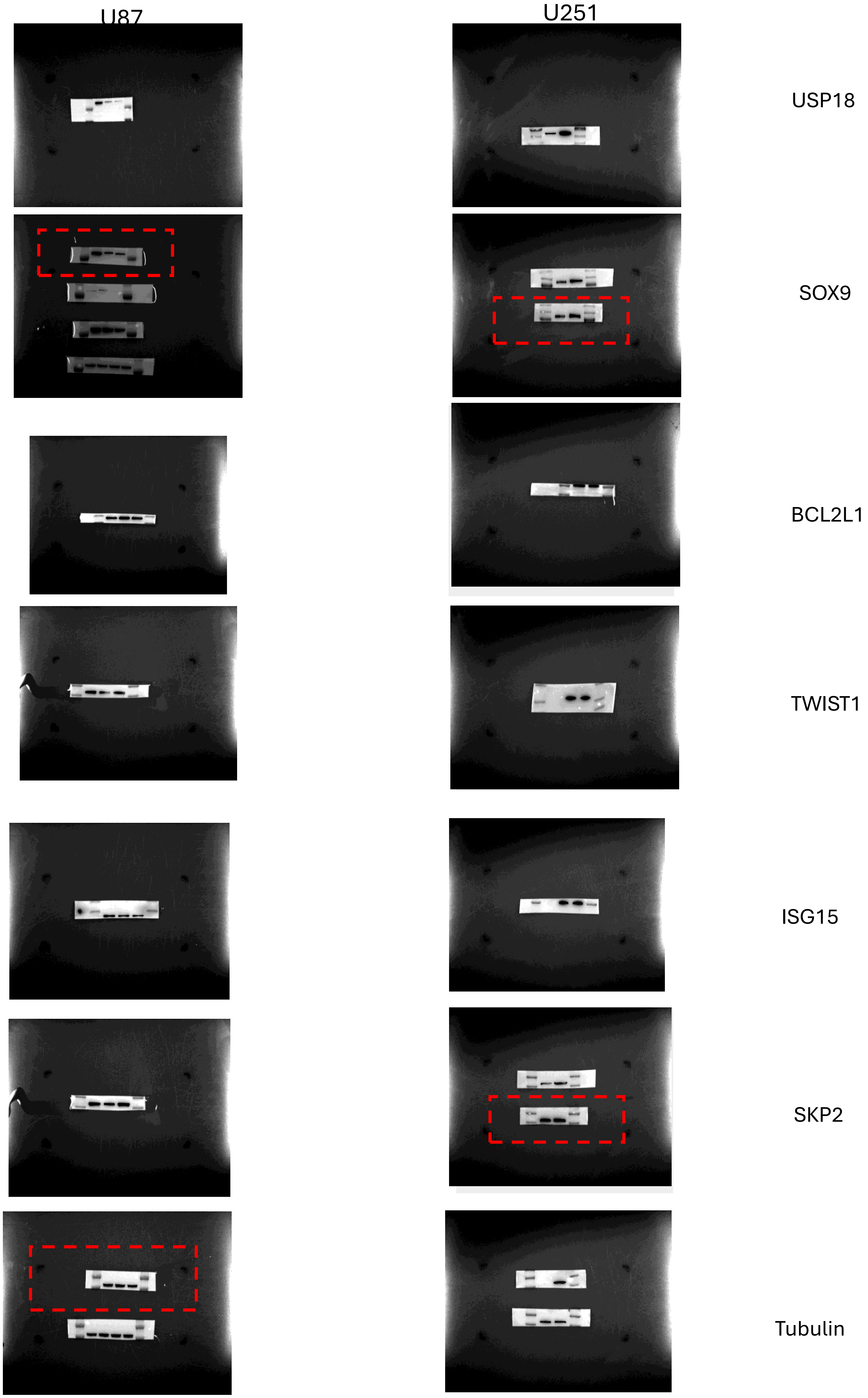

Supplement: Supplementary file 1 — original Western blot [file 41420_2025_2522_MOESM1_ESM.pdf]
