# Supplementary figures and images for: USP18 deubiquitinates and stabilizes SOX9 to promote the stemness and malignant progression of glioblastoma
Source: Cell Death Discov. 2025 May 15;11:237. doi: 10.1038/s41420-025-02522-9 (PMC12081856; doi:10.1038/s41420-025-02522-9)

**A**

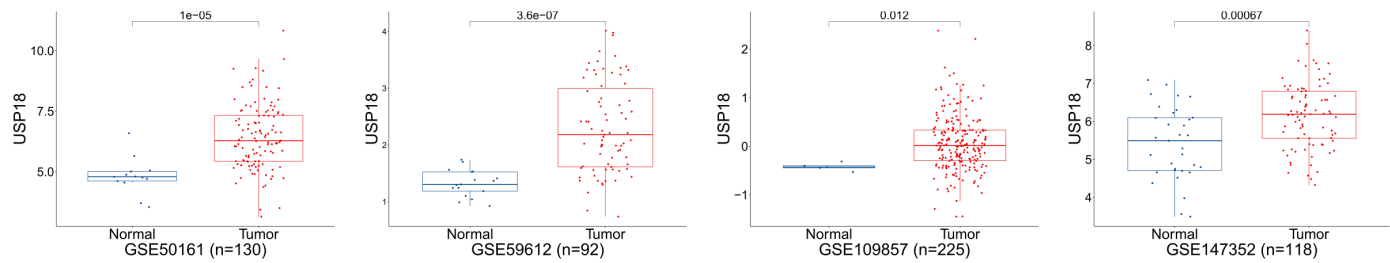

**B**

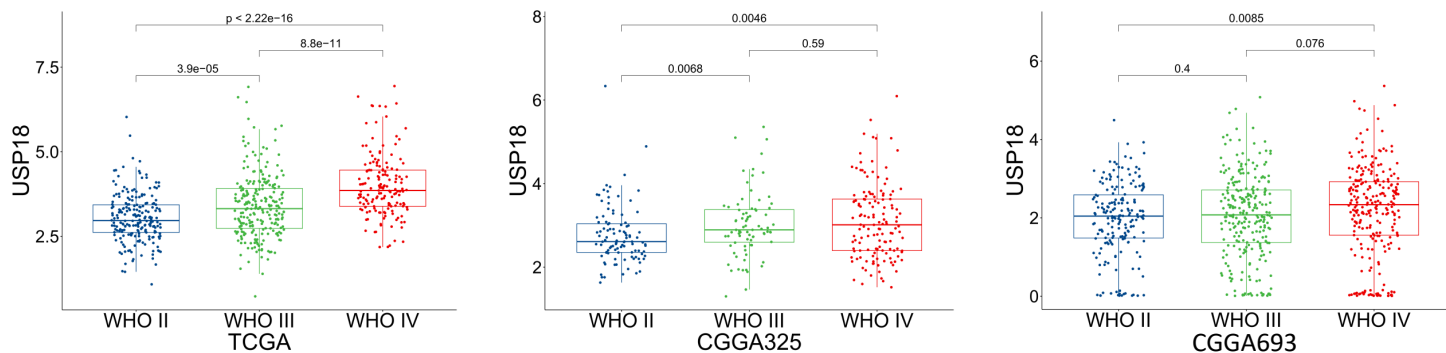

**C**

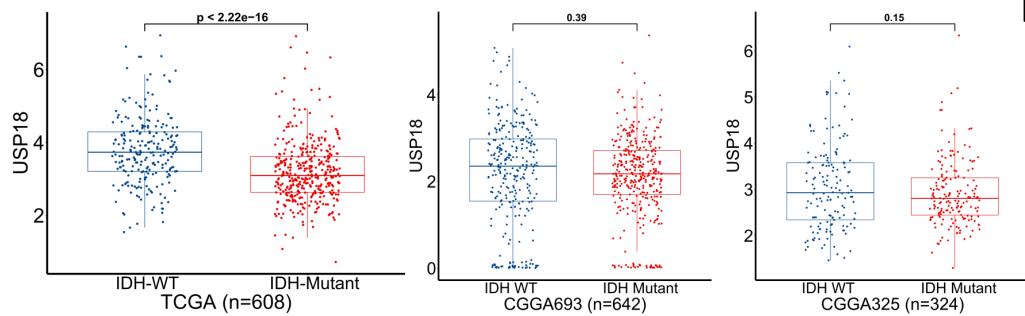

**E**

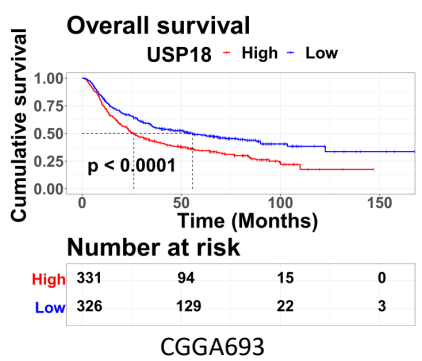

**D**

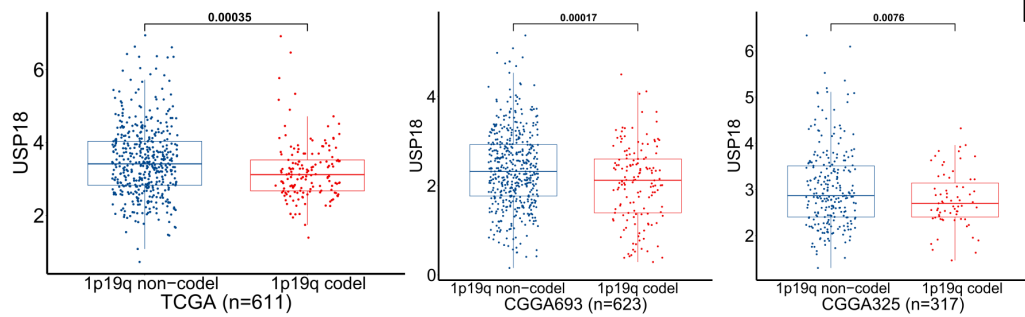

**F**

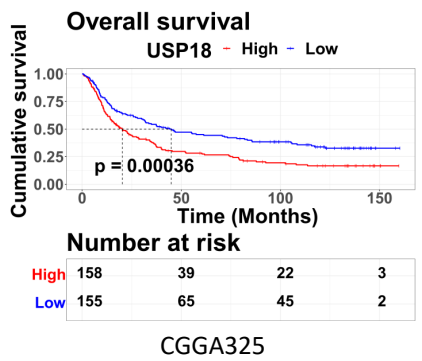

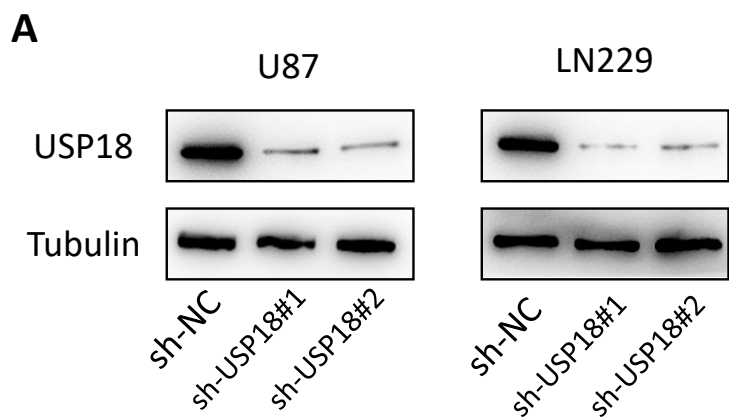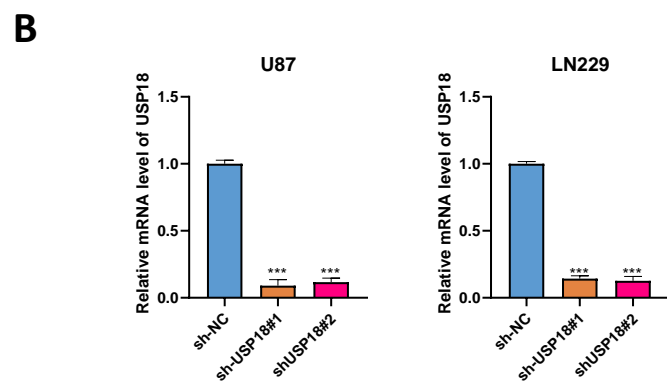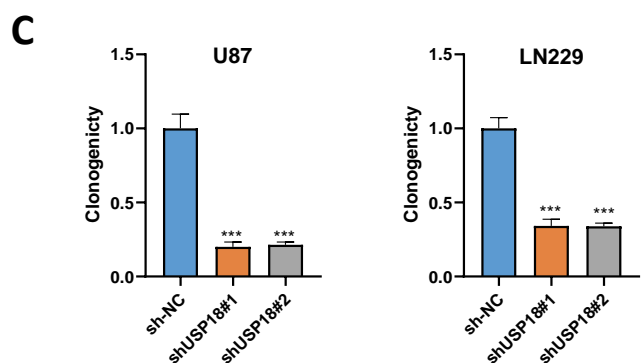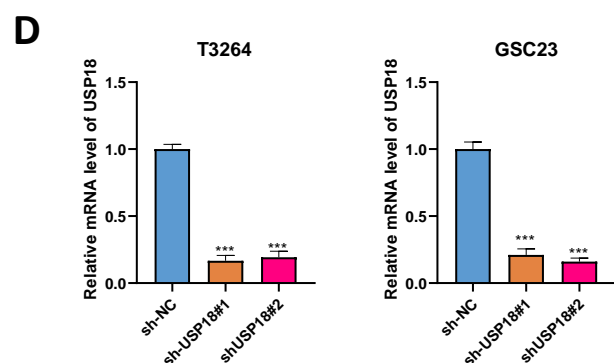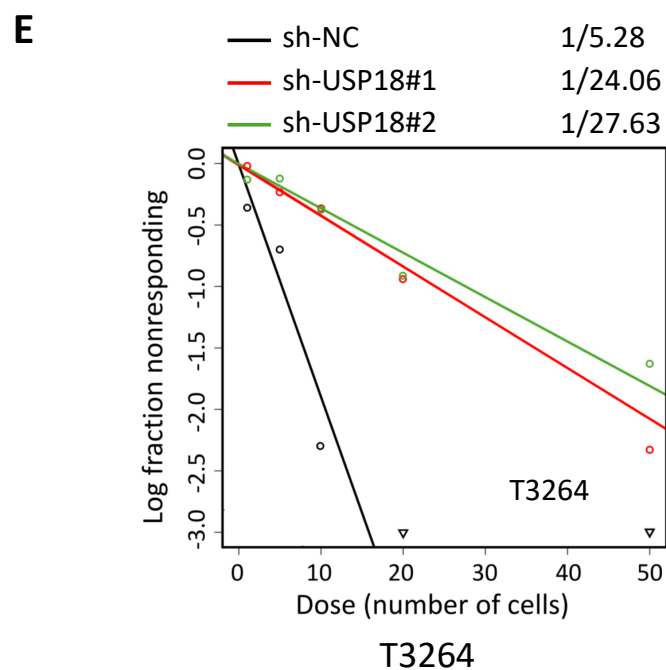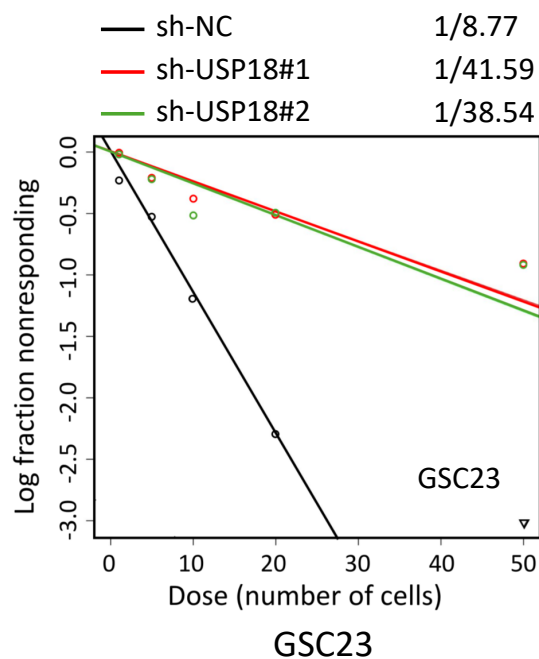

**A**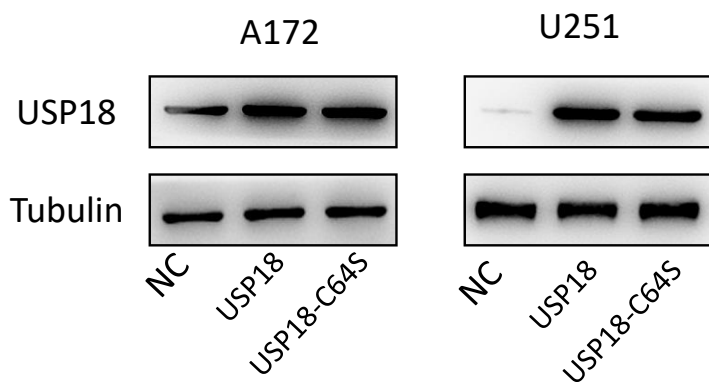**B**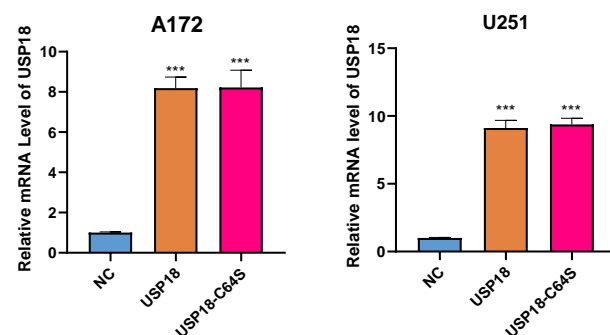**C**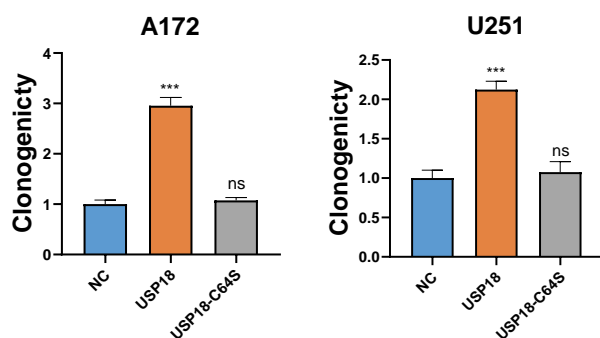**D**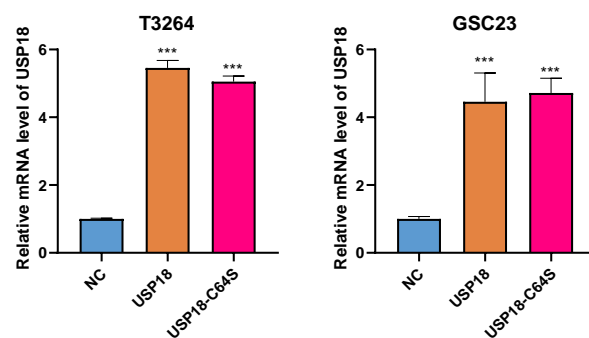**E**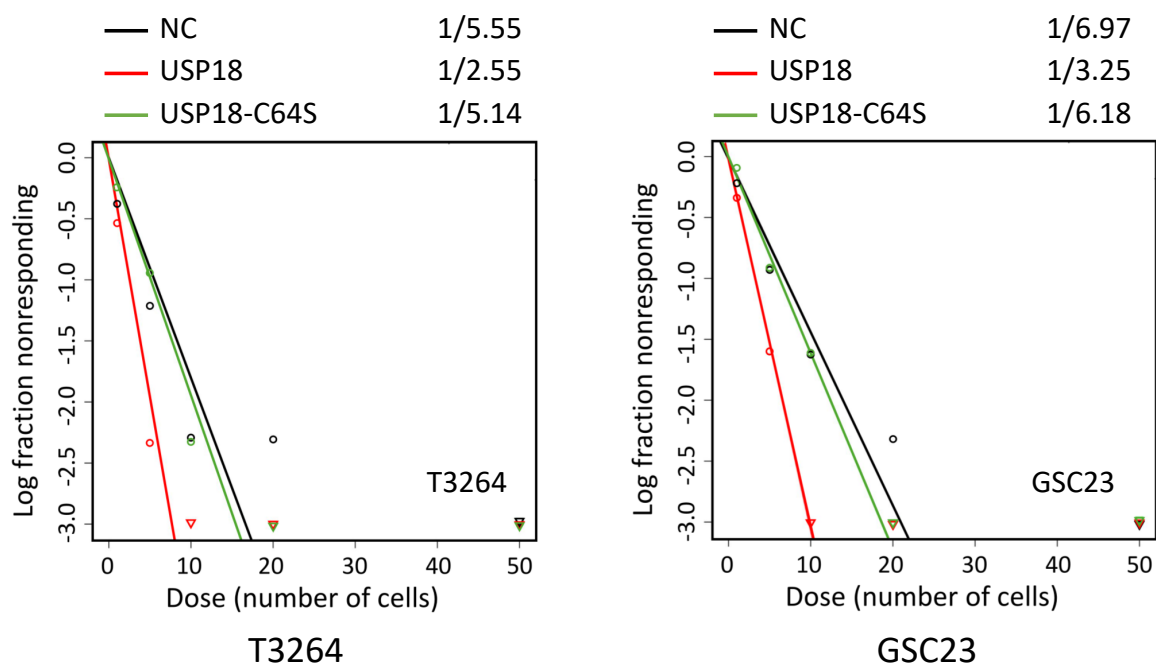

**A**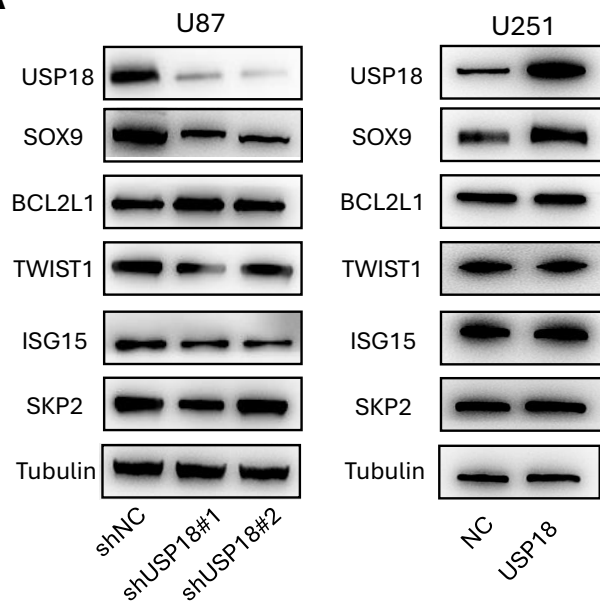**B**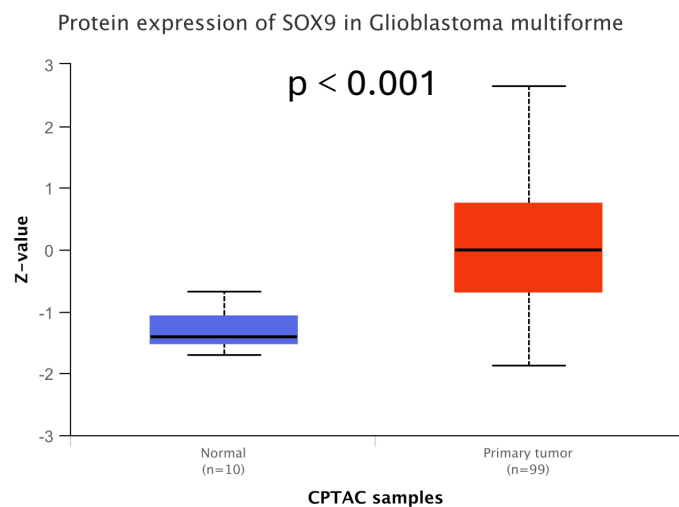**C**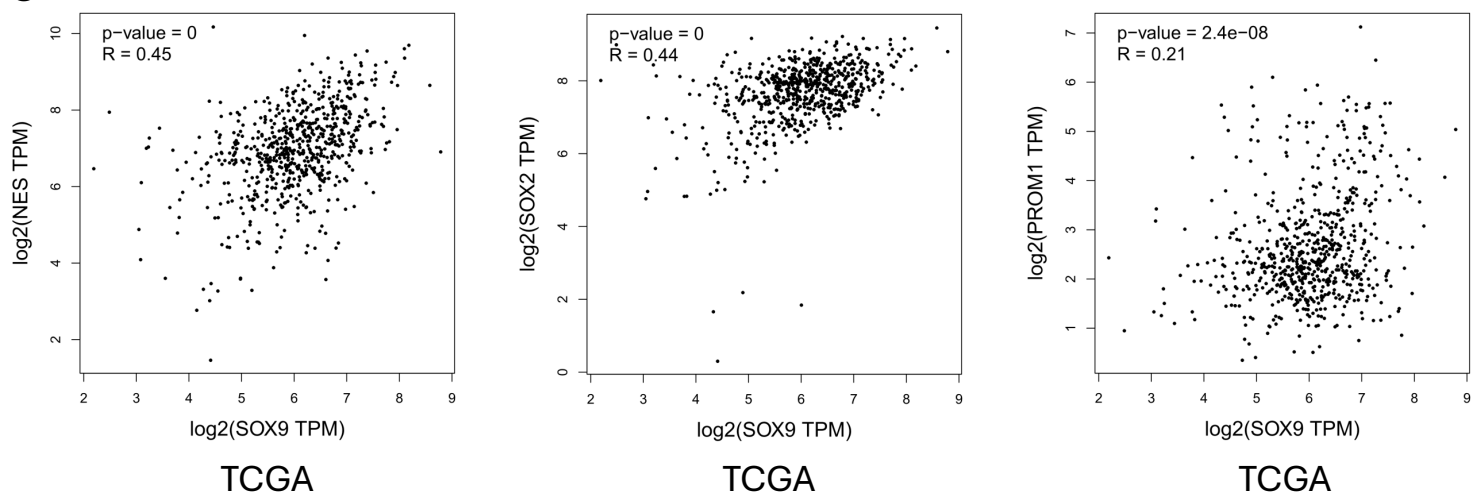

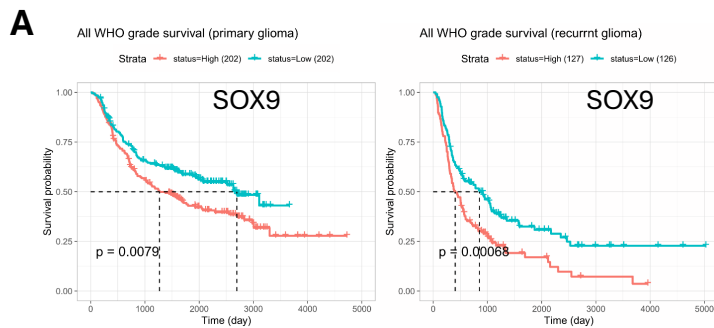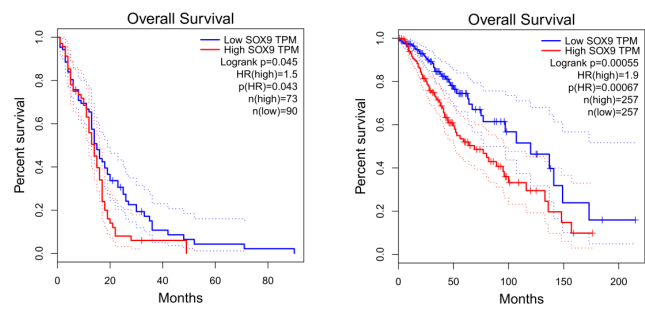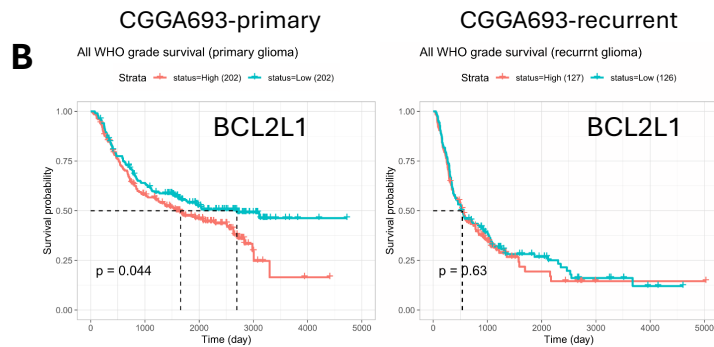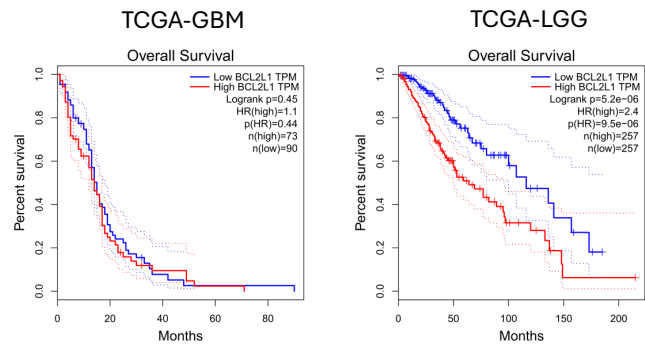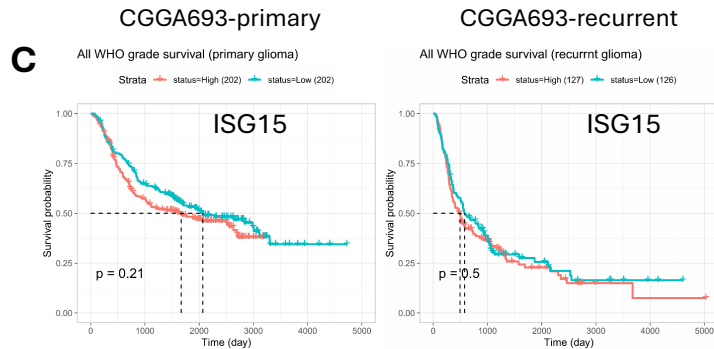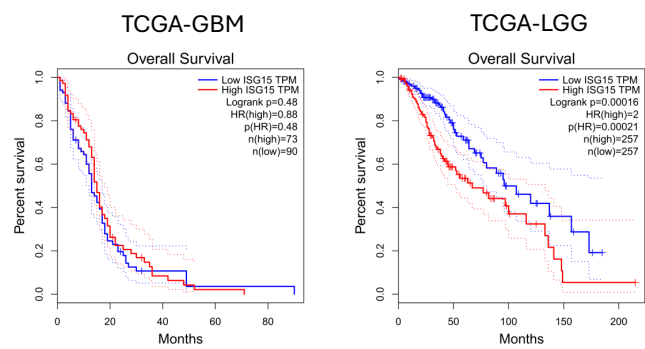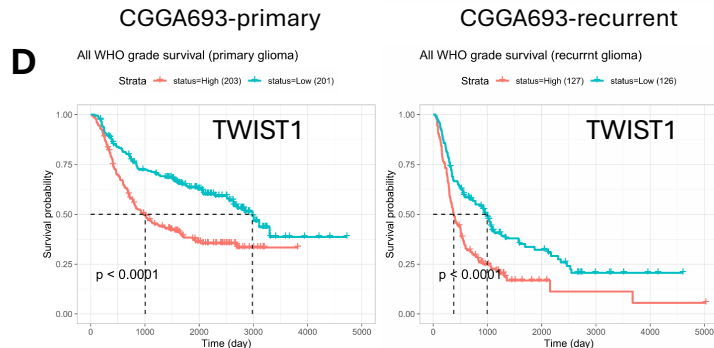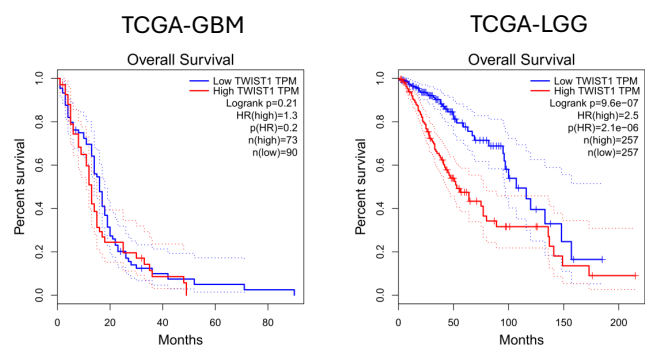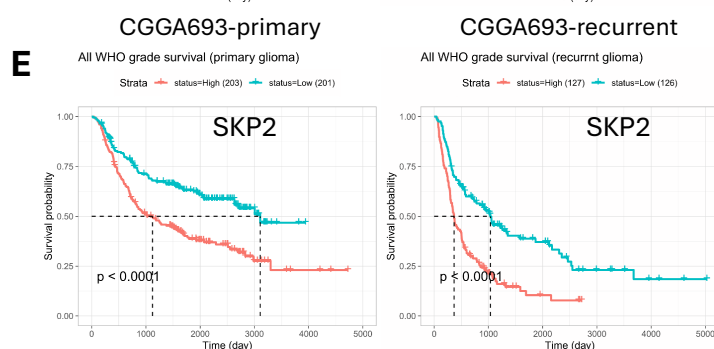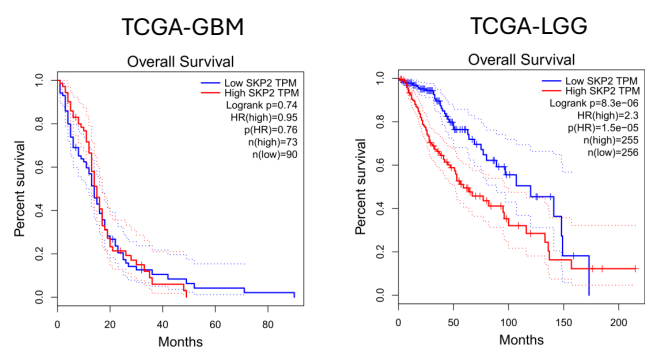

**A**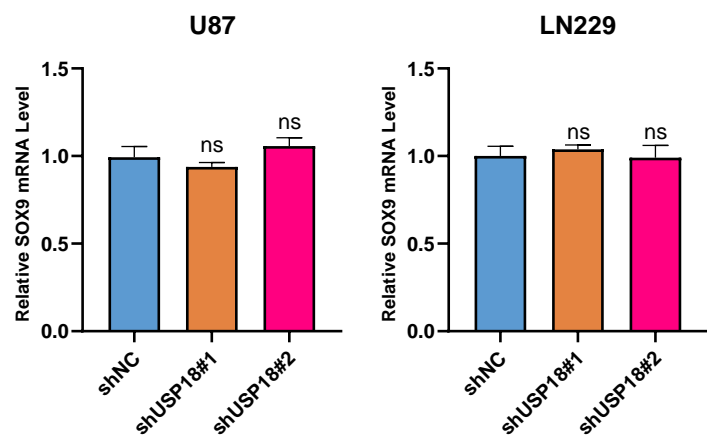**B**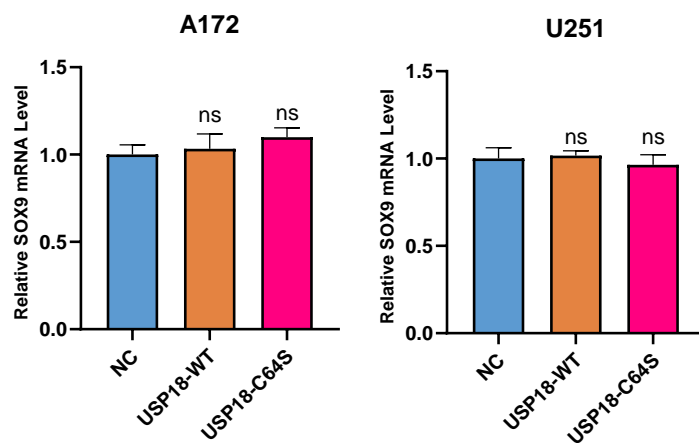**C**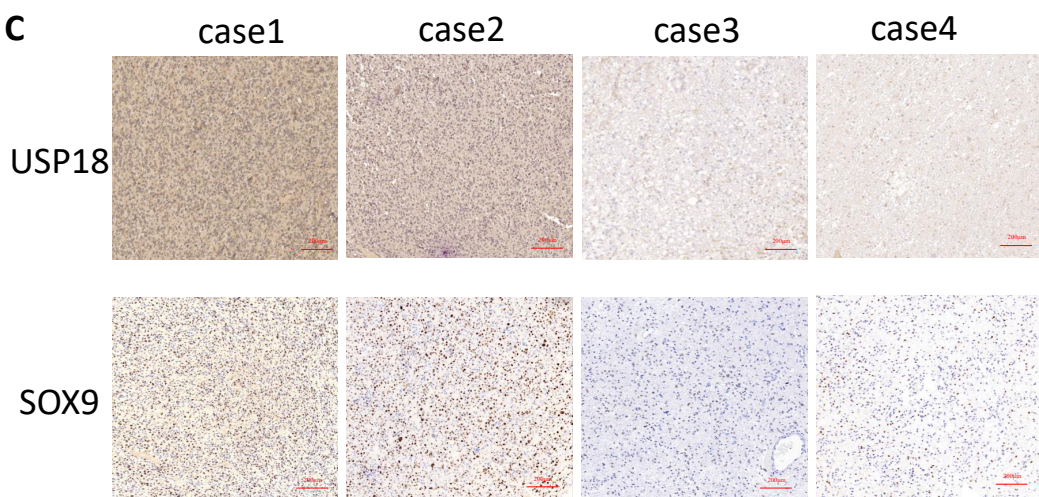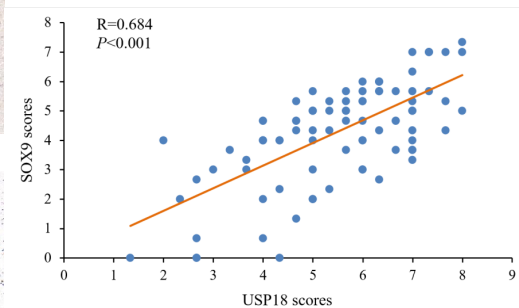**D**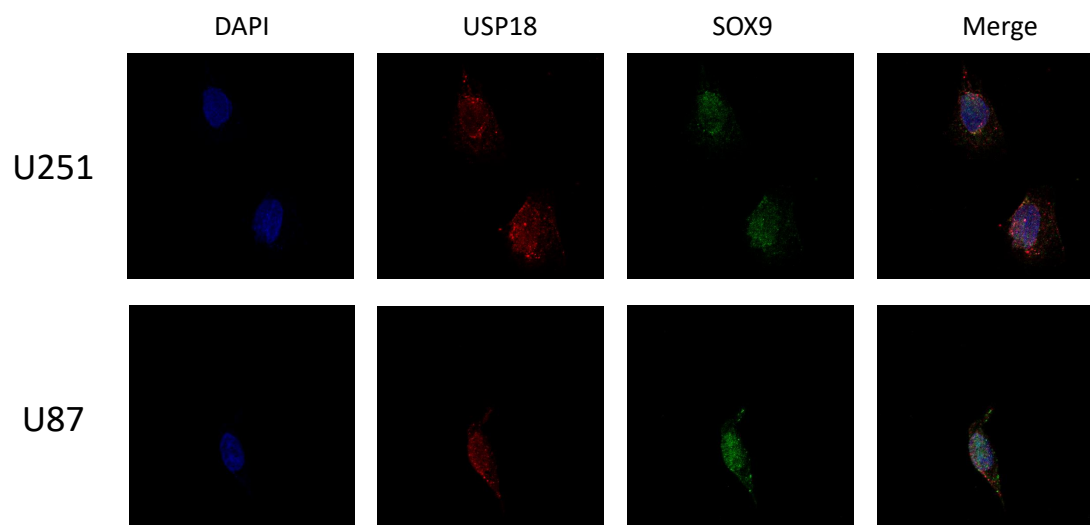

**A**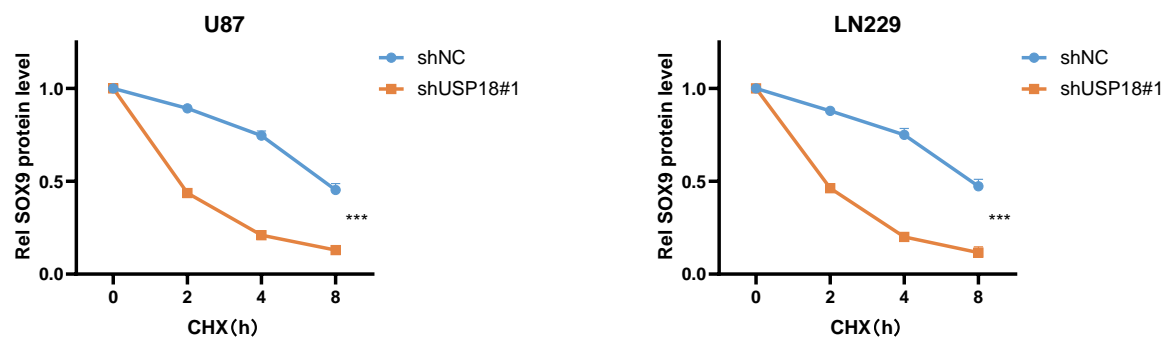**B**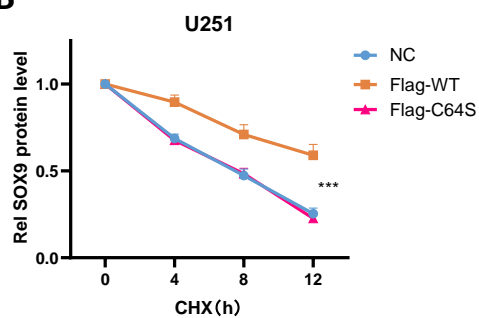**C**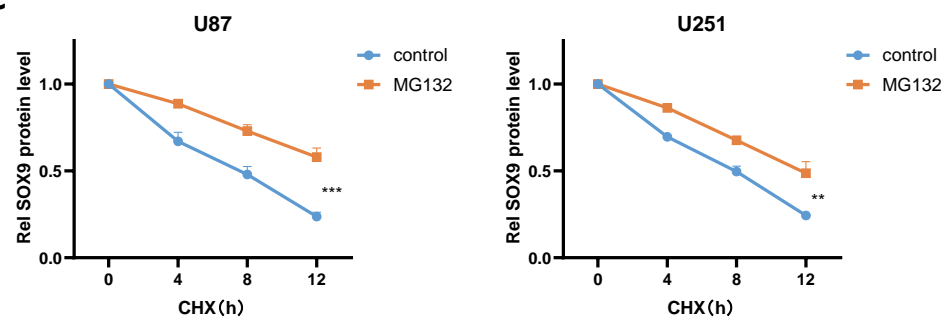**D**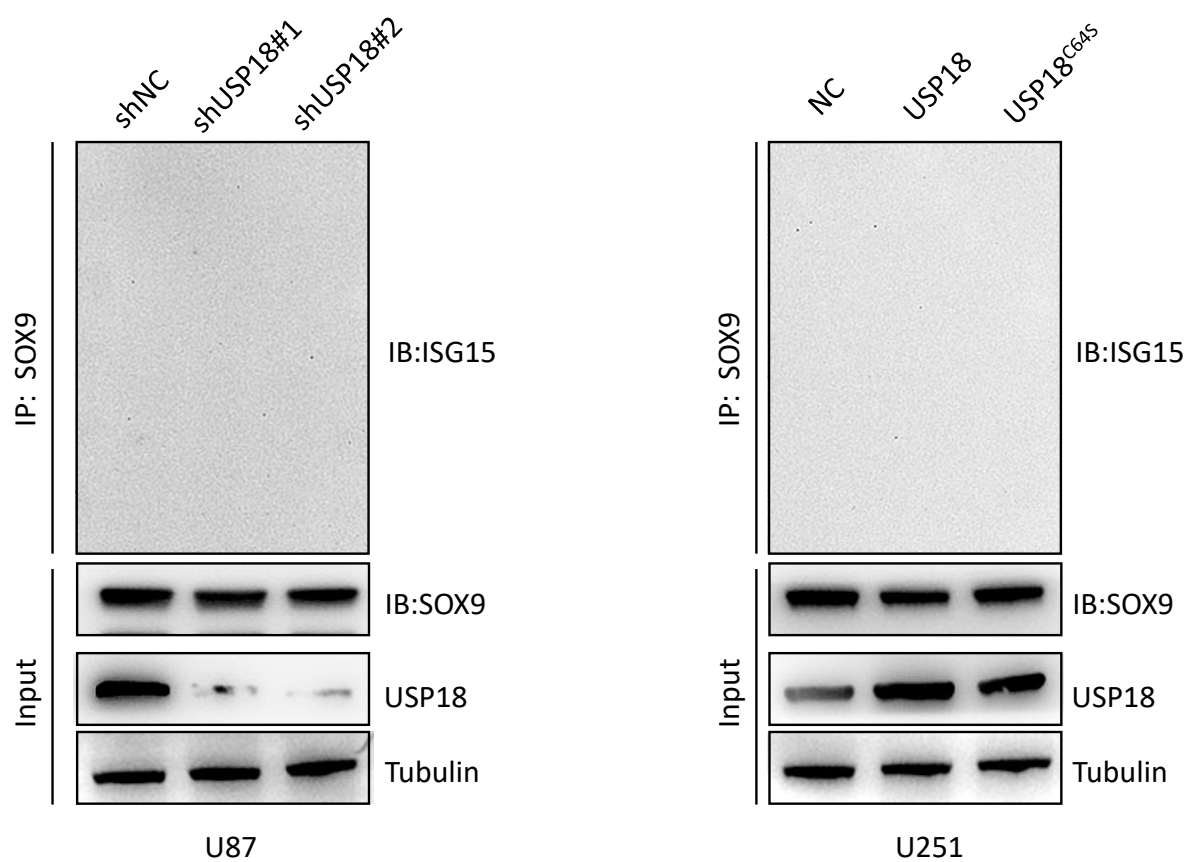

**A**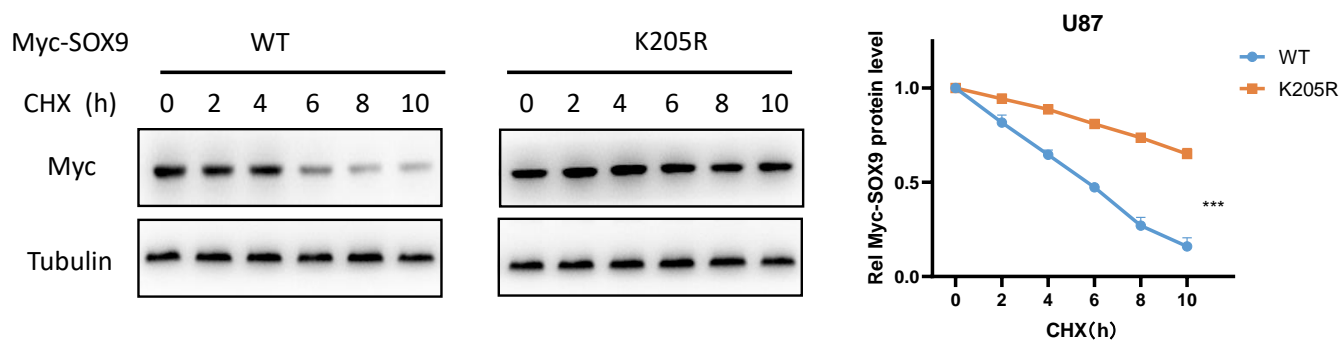**B**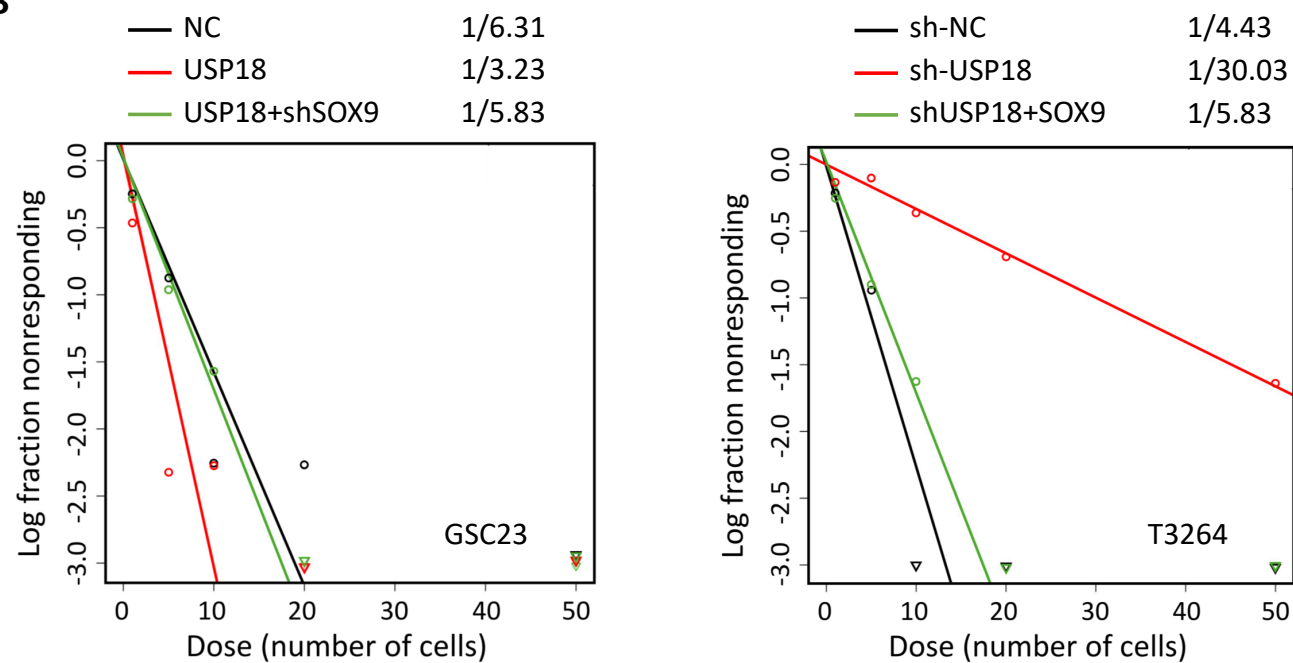**C**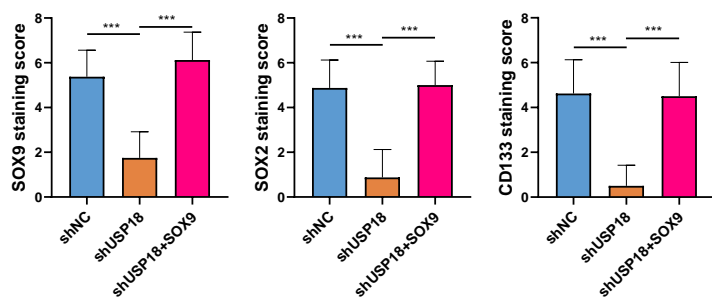**D**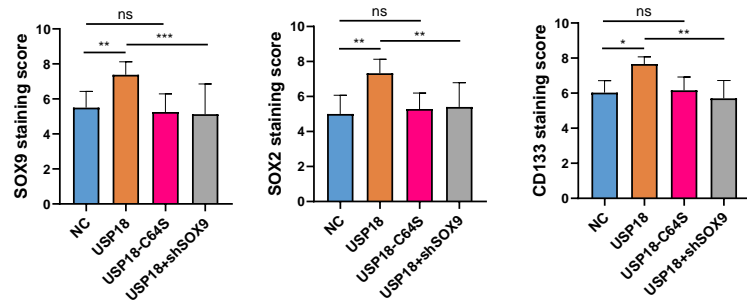

**A**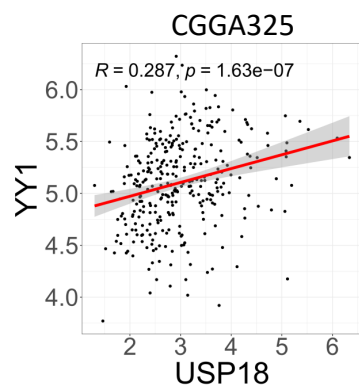**B**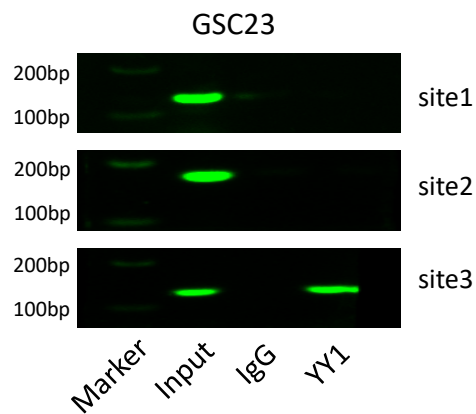**C**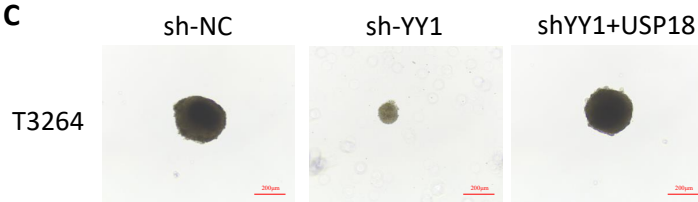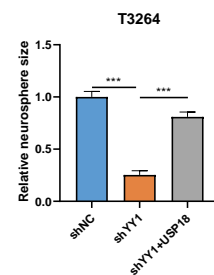**D**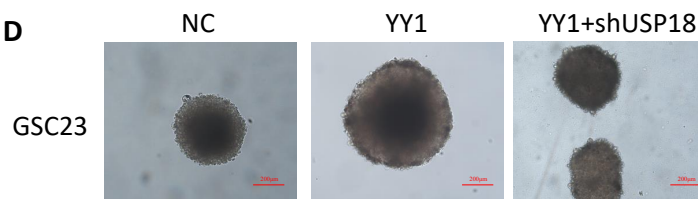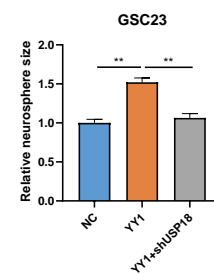**E**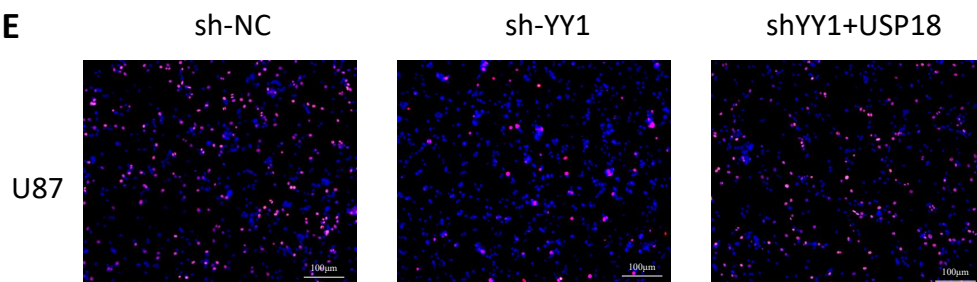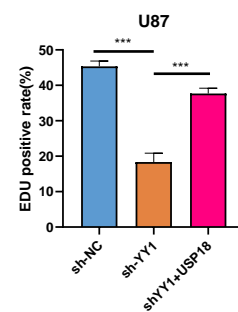**F**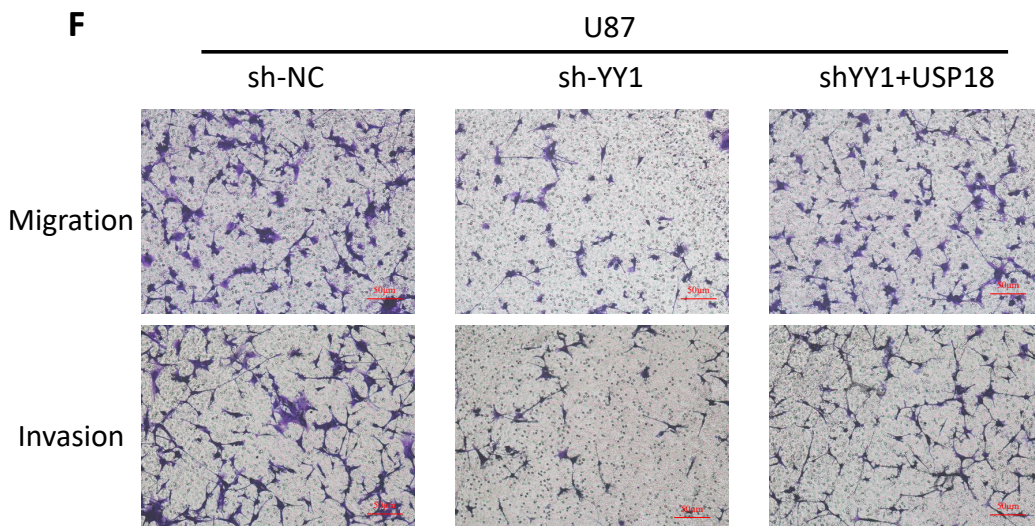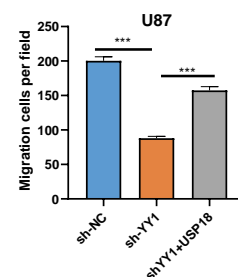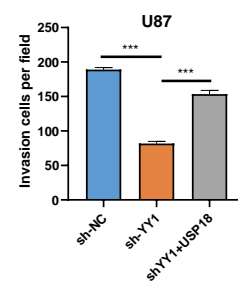

Supplement: Supplementary file 2 — Figure S1-S9 [file 41420_2025_2522_MOESM2_ESM.pdf]
