# Supplementary material for: USP18 deubiquitinates and stabilizes SOX9 to promote the stemness and malignant progression of glioblastoma
Source: Cell Death Discov. 2025 May 15;11:237. doi: 10.1038/s41420-025-02522-9 (PMC12081856; doi:10.1038/s41420-025-02522-9)
Supplement: Supplementary file 3 — Supplementary Figure S1-S9 legends [file 41420_2025_2522_MOESM3_ESM.docx]

**The legends of the supplementary figure**

**Figure S1**

A: USP18 expression in gliomas is significantly higher than in normal brain tissues across multiple glioma datasets.

B: In TCGA, CGGA325, and CGGA693 datasets, the expression of USP18 increases with higher WHO grades of gliomas.

C: Expression levels of USP18 in IDH-wildtype and IDH-mutant gliomas across TCGA, CGGA325, and CGGA693 datasets.

D: Higher USP18 expression in gliomas with 1p/19q co-deletion compared to those without, across TCGA, CGGA693, and CGGA325 datasets.

E: Differential survival outcomes based on USP18 expression levels in the CGGA datasets.

**Figure S2**

A: USP18 protein levels in U87 and LN229 cells following shRNA transfection.

B: USP18 mRNA levels in U87 and LN229 cells following shRNA transfection.

C: Quantitative analysis of colony formation experiments in Figure 2C.

D: USP18 mRNA levels in T3264 and GSC23 cells following shRNA transfection.

E: The limiting dilution experiments demonstrated that USP18 knockdown reduces the tumor sphere-forming frequency of T3264 and GSC23 cells.

All results are presented as the mean ± SD (three independent experiments). *p < 0.05, **p < 0.01, ***p < 0.001.

**Figure S3**

A-B: The protein and mRNA expression levels of USP18 after transfection with USP18 WT or USP18-C64S.

C: Quantitative analysis of colony formation experiments in Figure 3C.

D: USP18 mRNA levels in T3264 and GSC23 cells after transfection with USP18 WT or USP18-C64S.

E: The limiting dilution experiments demonstrated that USP18, but not USP18-C64S, enhanced the tumor sphere-forming frequency of U87-GSC and GSC23.

All results are presented as the mean ± SD (three independent experiments). *p < 0.05, **p < 0.01, ***p < 0.001.

**Figure S4**

A: Western blot analysis of candidate GSC-related proteins expression following USP18 overexpression or knockdown.

B: CPTAC data showing significantly higher SOX9 protein expression in glioma tissue compared to normal brain tissue.

C: Analysis of TCGA glioma dataset showing a significant positive correlation between SOX9 expression and NESTIN, SOX2, and CD133 (PROM1) expression.

**Figure S5**

A-E: Kaplan-Meier survival analysis based on the expression levels of potential USP18 interacting proteins.

**Figure S6**

A-B: Modulation of USP18 expression levels did not significantly affect SOX9 mRNA expression levels.

C: Representative immunohistochemistry images of USP18 and SOX9 expression in human glioma tissues (scale bar: 200 μm). Scatter plot showing the positive correlation between USP18 and SOX9 IHC scores in 90 glioma specimens. Note that the scores of
some samples overlap.

D: Immunofluorescence experiments show co-localization of USP18 protein and SOX9 protein within glioma cells.

All results are presented as the mean ± SD (three independent experiments). *p < 0.05, **p < 0.01, ***p < 0.001.

**Figure S7**

A: The quantitative analysis of the protein stability assays in Figure 4F showed that USP18 knockdown decreases the stability of SOX9 protein.

B: The quantitative analysis of the protein stability assay in Figure 4G showed that USP18 WT, but not USP18-C64S, significantly extended the half-life of endogenous SOX9 protein.

C: The quantitative analysis of the protein stability assay in Figure 4I showed that MG132 treatment alleviated the degradation rate of SOX9 protein under CHX treatment.

D: U87 cells with USP18 knockdown (shUSP18) or control (shCtrl) were subjected to co-immunoprecipitation (co-IP) using an anti-SOX9 antibody, followed by Western blotting with anti-ISG15 and anti-SOX9 antibodies. No ISG15-conjugated SOX9 was observed. U251 cells overexpressing wild-type USP18 (USP18-WT), catalytically inactive mutant (USP18-C64S), or empty vector (Vector) were analyzed for SOX9 ISGylation using the same co-IP/Western blotting approach. No ISGylation signal was detected in any condition. The cells were treated with 20 μM MG132 for 8 hours before being harvested.

All results are presented as the mean ± SD (three independent experiments). *p < 0.05, **p < 0.01, ***p < 0.001.

**Figure S8**

A: U251 cells transfected with Myc-SOX9 WT or Myc-SOX9 K205R mutant were treated with 20 µg/ml CHX for indicted time points (0, 2, 4, 6, 8, 10h) and were analyzed by western blot. Quantification of the expression levels of SOX9 was shown in the right panel.

B: The limiting dilution experiments shown the tumor sphere formation frequency of GSCs under different treatments.

C-D: Statistical analysis of IHC staining scores for SOX9, SOX2 and CD133 expression in tumor tissues.

All results are presented as the mean ± SD (three independent experiments). *p < 0.05, **p < 0.01, ***p < 0.001.

**Figure S9**

A: In CGGA325 datasets, the expression levels of YY1 and USP18 show a significantly positive correlation.

B: Design primers targeting three potential binding sites, perform ChIP-qPCR experiments using GSC23 cells lysates, and analyze the resulting products by agarose gel electrophoresis.

C: Neurosphere formation assays confirmed that YY1 knockdown impaired the neurosphere-forming capacity of GSCs, whereas overexpression of USP18 largely rescued this ability.

D: Neurosphere formation assays confirmed that YY1 overexpression enhanced the neurosphere-forming capacity of GSCs, which can be blocked by shUSP18.

E: The EdU experiments showed that knockdown of YY1 reduces the proliferation capability of glioma cells, while overexpression of USP18 significantly restores the proliferation capability of glioma cells.

F: Transwell migration and invasion assays indicated that depletion of YY1 decreases the invasion and migration capabilities of glioma cells, which can be restored by overexpression of USP18.

All results are presented as the mean ± SD (three independent experiments). *p < 0.05, **p < 0.01, ***p < 0.001.
