# Supplementary material for: USP18 deubiquitinates and stabilizes SOX9 to promote the stemness and malignant progression of glioblastoma
Source: Cell Death Discov. 2025 May 15;11:237. doi: 10.1038/s41420-025-02522-9 (PMC12081856; doi:10.1038/s41420-025-02522-9)
Supplement: Supplementary file 5 — Table S2-S3 [file 41420_2025_2522_MOESM5_ESM.docx]

| **Gene** | **Primer sequences(5'-3')** | |
| --- | --- | --- |
|  | **Forward** | **Reverse** |
| USP18 | CATGGCGCTTGAGAGATTCC | CAACCAGGCCATGAGGGTAG |
| SOX9 | CCAGGTGCTCAAAGGCTACG | GTTGGGGGAGATGTGCGT |
| YY1 | CATCTTTGGGGCGAGTGAGT | ATGGCATGGGGGTCTGAAAG |
| GAPDH | AAGGTCGGAGTCAACGGATTTG | CCATGGGTGGAATCATATTGGAA |
| Promoter 1 | CCATGAAGGACGGAAGGAAGAGC | TCTGAATCTTGGTAGGTCGGTGGAG |
| Promoter 2 | TCCATCACTTACCGTGGTTTCACTTAC | AAGAGGCTGAGGCAGGAGAATAACT |
| Promoter 3 | CCACACCTGTAATCCCAGCACTTT | GTTCAAGCGATTCTCCTGCCTCA |

**Table S2. The primer sequences used in this article.**

**Table S3. The shRNA sequences used in this article.**

| **NO.** | **sequence** |
| --- | --- |
| **shUSP18#1** | **ACTGCATATCTTCTGGTTTAC** |
| **shUSP18#2** | **CCTCATGGCCTGGTTGGTTTA** |
| **shSOX9** | **ACCTTCGATGTCAACGAGTTT** |
| **shYY1** | **GACGACGACTACATTGAACAA** |
